# Structural Metamorphoses of d-Xylose Oxetane- and Carbonyl Sulfide-Based Polymers *In Situ* during Ring-Opening Copolymerizations

**DOI:** 10.1021/jacs.3c05529

**Published:** 2023-08-14

**Authors:** David K. Tran, Ashley N. Braaksma, Autumn M. Andras, Senthil K. Boopathi, Donald J. Darensbourg, Karen L. Wooley

**Affiliations:** † Departments of Chemistry, Texas A&M University, College Station, Texas 77842, United States; ‡ Materials Science & Engineering, Texas A&M University, College Station, Texas 77842, United States; § Chemical Engineering, Texas A&M University, College Station, Texas 77842, United States

## Abstract

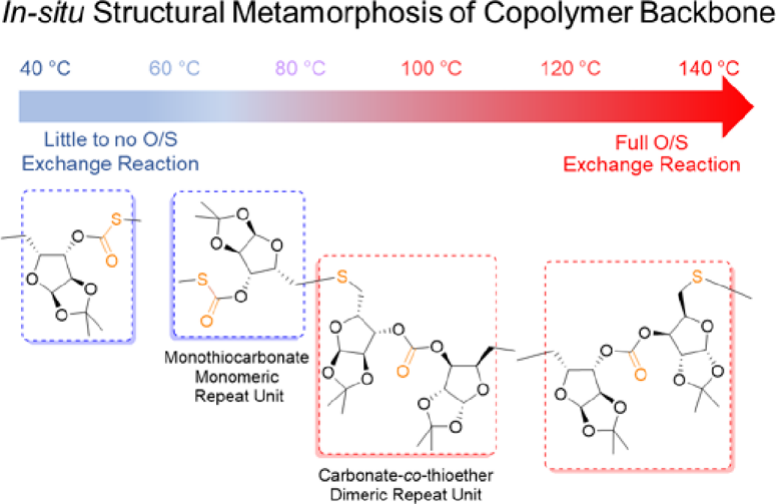

Polymers constructed from copolymerizations of carbohydrates with C1 feedstocks are promising targets that provide transformation of sustainably sourced building blocks into next-generation, environmentally degradable plastic materials. In this work, the initial intention was to expand beyond polycarbonates prepared by the copolymerization of oxetanes derived from d-xylose with CO_2_ and incorporate sulfur atoms through the establishment of monothiocarbonates that would provide the ability to modulate the backbone compositions and result in unique effects upon the chemical, physical, and mechanical properties. Therefore, the syntheses of poly(1,2-*O*-isopropylidene-α-d-xylofuranose monothiocarbonate)s were investigated by ring-opening copolymerizations of 3,5-anhydro-1,2-*O*-isopropylidene-α-d-xylofuranose with carbonyl sulfide (COS) facilitated by (salen)CrCl/cocatalyst systems. Unexpectedly, when copolymerization temperatures exceeded 40 °C, oxygen/sulfur exchange reactions occurred, causing *in situ* dynamic backbone restructuring through a series of inter-related and complex mechanistic pathways that transformed monothiocarbonate monomeric repeating units into carbonate and thioether dimeric repeating units. These backbone structural compositional transformations were investigated through a combination of Fourier transform infrared and nuclear magnetic resonance spectroscopic techniques and were demonstrated to be easily tuned *via* temperature and catalyst/cocatalyst stoichiometries. Furthermore, the regiochemistries of these d-xylose-based sulfur-containing polymers revealed that monothiocarbonate monomeric repeating units had a head-to-tail connectivity, while the carbonate and thioether dimeric repeating units had dual head-to-head and tail-to-tail connectivities. These sulfur-containing polymers exhibited enhanced thermal stabilities compared to their oxygen-containing polycarbonate analogues and revealed variations in the effects upon glass transition temperatures, demonstrating the effect of sulfur incorporation in the polymer backbone. These findings contribute to the advancement of sustainable polymer production by using feedstocks of natural origin coupled with COS.

## Introduction

The quest for degradable, sustainable polymers from renewable resources is becoming an increased priority, along with and in efforts to address coincident events of plastic pollution, climate change, and the energy transition away from petroleum-based feedstocks. There has been an emphasis on developing chemical transformations involving molecular or macromolecular materials of natural origin to afford functional polymers, thereby advancing efforts toward achieving sustainability for the growing plastic demand.^1−5^ Relevant to this aim, copolymerizations of small molecule natural products with carbon dioxide or other complementary feedstocks, some of which originate as waste from fossil fuel or human-made supplies, are of particular interest for the production of a broad range of types of sustainable polymeric materials.^1,6−12^ For instance, the general class of ring-opening copolymerizations (ROCOPs)^1,6−8,13−19^ of cyclic ethers with comonomers, such as carbon dioxide (CO_2_),^17,20−27^ carbonyl sulfide (COS),^28,29^ carbon disulfide (CS_2_),^28,30^ aryl isocyanates,^31,32^ and cyclic anhydrides,^6,7,14,19,33−38^ has provided access to diverse backbone compositions, including polycarbonates, poly(monothiocarbonate)s, poly(thiocarbonate)s, polyurethanes or polyallophanates, and polyesters, respectively. However, a common limitation to this copolymerization strategy is the use of cyclic ethers from non-renewable sources, which adversely affects overall sustainability. There is great interest, therefore, in transforming natural products into cyclic ethers as monomers for ROCOP.

Recently, we synthesized poly(1,2-*O-*isopropylidene-d-xylofuranose carbonate)s by the ROCOP of an oxetane derived from d-xylose and CO_2_.^20^ The dual copolymerization pathways that were involved served as processes for polycarbonate syntheses with enhanced sustainability by incorporating a feedstock of natural origin while avoiding phosgene or phosgene derivatives. However, the direct ring-opening copolymerization of 3,5-anhydro-1,2-*O-*isopropylidene-d-xylofuranose with CO_2_ required long reaction times that produced a six-membered cyclic carbonate in competition with the copolymer and formed a regioirregular polymer backbone with tail-to-tail, head-to-tail, and head-to-head regiochemistries. While this specific oxetane monomer has been used for a wide range of ROCOP with other monomers, including CS_2_^30^ and cyclic anhydrides,^30,33^ the ROCOP of COS with this oxetane derived from d-xylose had yet to be investigated.

ROCOP of COS has been widely demonstrated and studied with aliphatic, alicyclic, and aromatic substituted epoxides^29,39−42^ or simply oxetane^43^ to afford sulfur-containing polymers, where regioregular poly(monothiocarbonate)s have been formed due to high regioselectivity during the copolymerization. In addition, the incorporation of sulfur as an element within polymer backbones has resulted in different thermal, mechanical, and optical properties compared to their non-sulfur analogues. For example, semicrystalline poly(trimethylene monothiocarbonate) has been synthesized from oxetane and COS, which displayed a melting transition (*T*_m_ = 128 °C). In contrast, its non-sulfur poly(trimethylene carbonate) counterpart was amorphous and displayed only a glass transition temperature (*T*_g_ = −20 °C).^43^ One main challenge in this process is controlling the composition and structure of the polymer backbone because of oxygen/sulfur exchange reactions (O/S ERs) that compete with ROCOP. The prevalence of O/S ERs during copolymerization controls the extent to which ether, thioether, carbonate, monothiocarbonate, and dithiocarbonate linkages develop, which may also produce regioirregular polymeric products with poor selectivity.^28,44−46^ Although the mechanism for this phenomenon is not fully understood, these O/S ERs can be suppressed by using low reaction temperatures, dry reaction systems, and/or Lewis pairs as catalysts.^44^

**Scheme 1:**
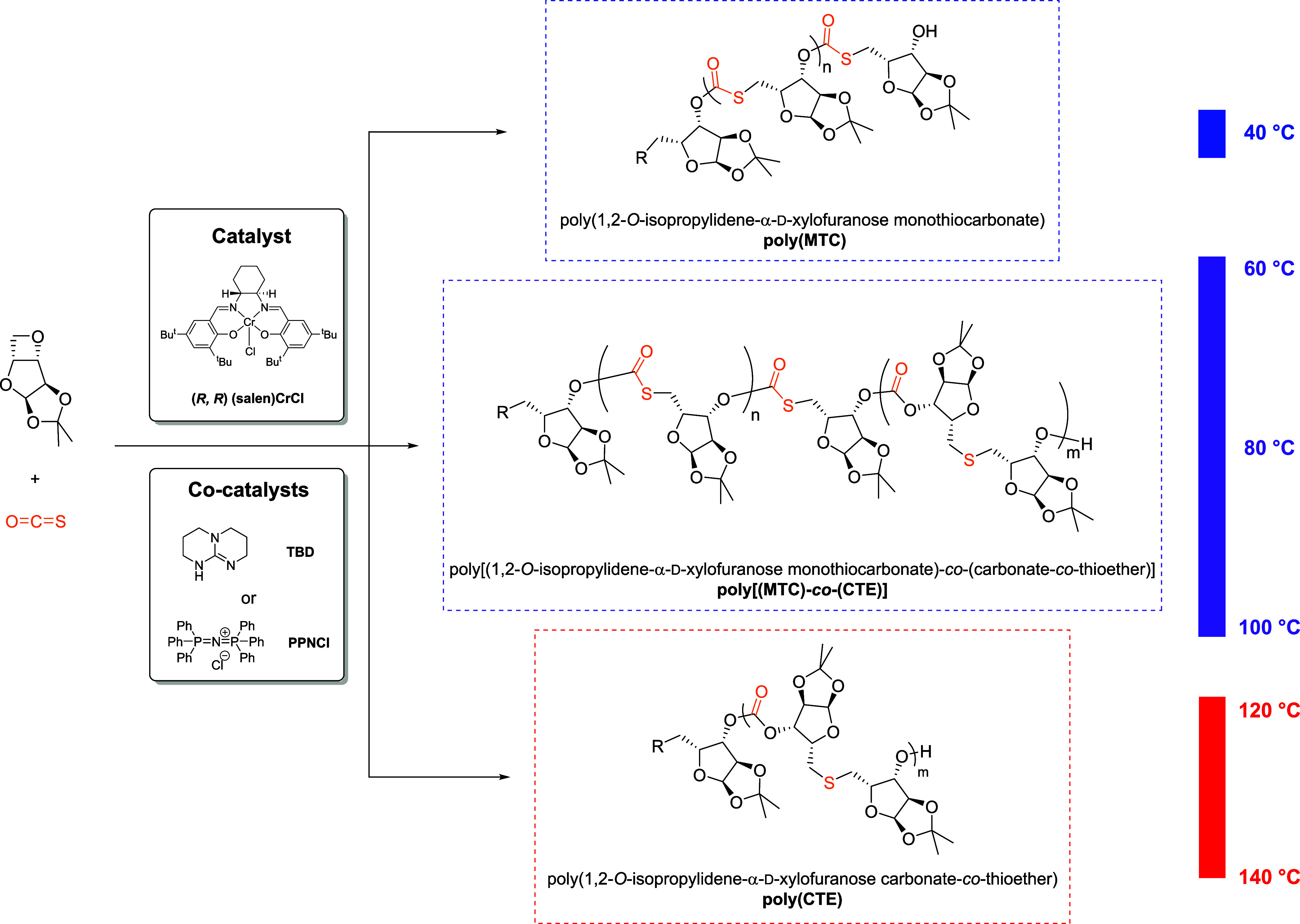
Synthesis of Poly(1,2-*O*-isopropylidene-α-d-xylofuranose monothiocarbonate), Poly[(1,2-*O*-isopropylidene-α-d-xylofuranose monothiocarbonate)-*co*-(carbonate-*co*-thioether)], and Poly(1,2-*O*-isopropylidene-α-d-xylofuranose carbonate-*co*-thioether) through Ring-Opening Copolymerization Using 3,5-Anhydro-1,2-*O*-isopropylidene-α-d-xylofuranose and COS at 40, 60–120, and 120–140 °C, Respectively

To address challenges with our d-xylose-based oxetane/CO_2_ system and expand the scope of polymer backbone composition to include S, we investigated the ROCOP of d-xylose oxetane with COS, anticipating the formation of poly(1,2-*O*-isopropylidene-α-d-xylofuranose monothiocarbonate) (poly(MTC)). Although each challenge mentioned above was addressed, new surprises were revealed due to tunable extents to which O/S ERs occurred. By variation of the temperature and the nature and stoichiometry of the catalyst/cocatalyst, ROCOPs were conducted from extreme cases of essentially no O/S ER to extensive O/S ER, affording copolymers having compositions ranging from poly(MTC) with monomeric monothiocarbonate repeating units to poly(1,2-*O*-isopropylidene-α-d-xylofuranose carbonate-*co*-thioether) (poly(CTE)) with dimeric repeating units. Intermediate poly[(1,2-*O*-isopropylidene-α-d-xylofuranose monothiocarbonate)-*co*-(carbonate-*co*-thioether)] (poly[(MTC)-*co*-(CTE)]) copolymers were also accessed, having combinations of monomeric and dimeric repeating units connected *via* monothiocarbonate, carbonate, and thioether linkages. Herein, we describe the ROCOP of xylose oxetane with COS to form sugar-based sulfur-containing polymers with structural metamorphoses occurring *in situ* during the copolymerizations, thereby offering opportunities to tune the backbone compositions and regiochemistries (Scheme 1).

## Results and Discussion

Based on previous investigations with oxetane and COS,^43^ copolymerizations of 3,5-anhydro-1,2-*O*-isopropylidene-α-d-xylofuranose (xylose oxetane) and COS were initially performed in stainless steel autoclave reactors in a well-ventilated chemical fume hood using (salen)CrCl as the metal catalyst and bis(triphenylphosphine)iminium chloride (PPNCl) as the cocatalyst with a [catalyst]:[cocatalyst]:[xylose oxetane]:[COS] ratio of 1:1:150:300 at 40 °C for 4 h (entry 1 in Table 1). The reaction mixture was then allowed to cool to room temperature, and the reactor was depressurized resulting in a crude solid amber product. An aliquot was taken for ^1^H NMR analysis, which determined that 27% monomer conversion had occurred with a >99% selectivity for the copolymer over the cyclic monothiocarbonate small molecule. Given that this high selectivity was observed at this low value of monomer conversion suggested that the copolymer resulted from the direct copolymerization of xylose oxetane and COS, with little to no formation and ring-opening of a cyclic monothiocarbonate (often produced by a back-biting reaction during the copolymerization). Purification was performed by dissolution in tetrahydrofuran (THF) followed by precipitation in methanol thrice to afford a white solid (polymer ***1***) with a number-average molar mass (*M*_n_) of 3.4 kDa and a dispersity (*Đ*) of 1.17. The absence of ether ^1^H NMR signals confirmed that the degrees of xylose oxetane and COS alternation were >99%. ^13^C NMR spectroscopy of ***1*** indicated that no O/S ER occurred as there was a single resonance observed at 169.9 ppm, the characteristic resonance frequency for a monothiocarbonate linkage (Figure S1). Although poly(MTC) was afforded at 40 °C, its molar mass was relatively low.

**Table 1:** Alternating COS/Xylose Oxetane Copolymerization Catalyzed by the (Salen)CrCl/Cocatalyst Catalytic System^a^

entry	name	cocatalyst	Cat:Cocat	*T* (°C)	% conversion	copolymer selectivity^b^	(MTC)/(CTE)^c^	*M*_n_ (kDa)^d^	*Đ*^d^
1	polymer **1**	PPNCl	1:1	40	27	>99/0	>99/0	3.4	1.17
2	polymer **2**	PPNCl	1:1	60	71	96/4	63/37	13.1	1.06
3	polymer **3**	PPNCl	1:1	80	80	96/4	62/38	13.8	1.09
4	polymer **4**	PPNCl	1:1	100	83	98/2	32/68	21.9	1.17
5	polymer **5**	PPNCl	1:1	120	93	97/3	32/68	23.2	1.08
6	polymer **6**	PPNCl	1:1	140	90	98/2	25/75	24.6	1.21
7	polymer **7**	PPNCl	1:2	40	29	>99/0	>99/0	3.8	1.10
8	polymer **8**	PPNCl	1:2	60	77	94/6	68/32	11.7	1.10
9	polymer **9**	PPNCl	1:2	80	83	92/8	60/40	12.6	1.06
10	polymer **10**	PPNCl	1:2	100	88	95/5	38/62	15.5	1.14
11	polymer **11**	PPNCl	1:2	120	78	97/3	0/>99	23.5	1.16
12	polymer **12**	PPNCl	1:2	140	93	97/3	0/>99	27.9	1.17
13	polymer **13**	TBD	1:2	100	98	99/1	30/70	13.1	1.28
14	polymer **14**	TBD	1:2	110	83	99/1	25/75	14.3	1.28
15	polymer **15**	TBD	1:2	120	98	99/1	22/78	14.3	1.35
16	polymer **16**	TBD	1:2	130	85	99/1	0/>99	13.2	1.30
17	polymer **17**	TBD	1:2	140	99	99/1	0/>99	14.3	1.30

a Reactions were performed in a 10 mL stainless steel autoclave reactor at molar ratios of [d-xylose oxetane]:[COS] = 150:300 for 4 h.

b Copolymer selectivity is reported as molar ratios of the copolymer *vs* cyclic monothiocarbonate, as determined by ^1^H NMR spectroscopy upon aliquots collected from crude reaction mixtures.

c Ratios of monothiocarbonate monomeric repeating units to carbonate and thioether dimeric repeating units were determined by ^1^H NMR spectroscopy of purified samples.

d Number-average molar masses and dispersities of polymers isolated by precipitation into methanol were determined by size exclusion chromatography using THF as the eluent, calibrated with polystyrene standards.

To attempt to produce polymers having higher molar mass, ROCOP reactions were then conducted at elevated temperatures, *i.e.*, 60–140 °C (entries 2–6 in Table 1). Upon increasing the temperature, high selectivity (>90%) for copolymer over cyclic monothiocarbonate was retained, as observed in the ^1^H NMR spectra of the crude mixtures. Furthermore, higher molar masses were achieved, with polymer ***6*** having the highest *M*_n_ value of 24.6 kDa while maintaining a low dispersity (*Đ* = 1.21). The degree of xylose oxetane and COS alternation remained at >99% with a lack of ether formation; however, O/S ERs occurred, resulting in poly[(MTC)-*co*-(CTE)] having combinations of monothiocarbonate, carbonate, and thioether linkages along the polymer backbone. The formation of monothiocarbonate and carbonate functionalities was determined by the presence of two signals in the ^13^C NMR spectra resonating at 169.9 and 153.5 ppm, respectively (Figures S2–S6). To our surprise, dithiocarbonates, another possible linkage during O/S ERs, were not observed, as confirmed by the absence of a characteristic resonance signal at *ca*. 190 ppm. It should be noted that when simple oxetane and COS were copolymerized, O/S ERs occurred at higher temperatures >140 °C.^43^ However, the ROCOP of xylose oxetane behaves similarly to the copolymerization of propylene oxide and COS with (salen)CrCl and PPNCl as the catalyst and cocatalyst, where O/S ERs occurred at temperatures ≥60 °C.^40^

Fourier transform infrared (FT-IR) and ^1^H NMR spectroscopic analyses of polymers ***1*–*6*** allowed for further confirmation of the backbone composition, including with quantification of the extent of monothiocarbonate monomeric repeating units *vs* O/S ERs leading to the transformation to carbonate and thioether dimeric repeating units. Qualitatively, FT-IR spectra revealed a correlation between two stretching bands at 1712 and 1751 cm^–1^ corresponding to monothiocarbonate *vs* carbonate carbonyls, respectively. For ***1*** prepared at 40 °C, a single carbonyl stretching band at 1712 cm^–1^ was observed. As the copolymerization temperature increased from 60 to 140 °C, the intensities of the carbonyl signals at 1712 cm^–1^ decreased while the intensities of the carbonyl peaks at 1751 cm^–1^ increased (Figure S20). This trend was also parallelly observed in the ^13^C NMR spectra of the samples, where the intensity of the resonance at 169.9 ppm corresponding to monothiocarbonate decreased, while the intensity of the carbonate carbon signal at 153.5 ppm increased with increasing copolymerization temperature (Figure S18). ^1^H NMR analysis of polymers ***2*–*6*** was used to quantify the ratio between the monothiocarbonate monomeric repeating units to the carbonate and thioether dimeric repeating units of these samples by integration of their resonances at 3.24–2.94 and 2.89–2.60 ppm, corresponding to the methylene protons adjacent to the monothiocarbonate and the methylene protons adjacent to the thioether, respectively (Figures S2–S6 and Table 1).

Intrigued by the above results, copolymerization reaction conditions were reconsidered to determine whether O/S ERs could be advantageously used to fully convert monothiocarbonate monomeric repeating units and afford polymers selectively containing carbonate and thioether dimeric repeating units, poly(CTE). Accordingly, the [catalyst]:[cocatalyst] ratio was increased from 1:1 to 1:2 while maintaining a [xylose oxetane]:[COS] ratio of 150:300 and ROCOP temperatures between 40 and 140 °C (entries 7–12 in Table 1). As for the 1:1 conditions, the molar mass increased with increasing temperature, and polymers ***7*–*12*** were obtained with similar molar masses as had been obtained at the lower cocatalyst loading. FT-IR analysis of ***7*–*12*** displayed a trend similar to polymers ***1*–*6***, where the intensity of the monothiocarbonate carbonyl stretch at 1712 cm^–1^ decreased, while the intensity of the carbonate carbonyl band at 1751 cm^–1^ increased with increasing copolymerization temperature (Figure S21). Interestingly, FT-IR analysis of polymers ***11*** and ***12***, which were synthesized at ≥120 °C, indicated that ROCOP of xylose oxetane with COS underwent extensive O/S ER to afford copolymers composed approximately exclusively of carbonate and thioether dimeric repeating units (>99%). This observation was further confirmed by their ^1^H NMR spectra, where polymers ***11*** and ***12*** displayed signals at 2.89–2.60 ppm, corresponding to the methylene protons of the thioether linkages, yet had an absence of methylene protons resonating at 3.17–3.07 ppm, characteristic of the methylene protons adjacent to monothiocarbonates (Figures S11 and S12). Further, the ^13^C NMR spectra of ***11*** and ***12*** displayed a single peak at 153.2 ppm, suggesting regioregularity along the polymer backbone. Copolymers ***7*–*10***, which were synthesized at 60 °C ≤ *T* < 120 °C, contained combinations of monothiocarbonate, carbonate, and thioether linkages, as demonstrated by their ^1^H NMR, ^13^C NMR (Figure S19), and FT-IR (Figure S22) spectra.

The effect of the nature of the cocatalyst was investigated by using 1,5,7-triazabicyclo[4.4.0]dec-5-ene (TBD) since oxetane and COS ROCOPs involving TBD have been shown to not undergo O/S ERs, even at higher temperatures.^43^ In this work, however, it was observed that O/S ERs did occur during the ROCOP of xylose oxetane and COS involving TBD at temperatures of 100–140 °C (Table 1, entries 13–17), as indicated by ^13^C NMR spectroscopy (Figure S20). The copolymer selectivity was maintained and perhaps improved slightly *vs****1*–*12*** (>99%). In addition, it is worth mentioning that higher selectivity for poly(CTE) (>99%) *vs* poly[(MTC)-*co*-(CTE)] was obtained only at ≥130 °C, which differs slightly from the result obtained for polymer ***11*** using PPNCl as the cocatalyst, which achieved >99% selectivity containing only carbonate and thioether dimeric repeating units at ≥120 °C.

**Figure 1:**
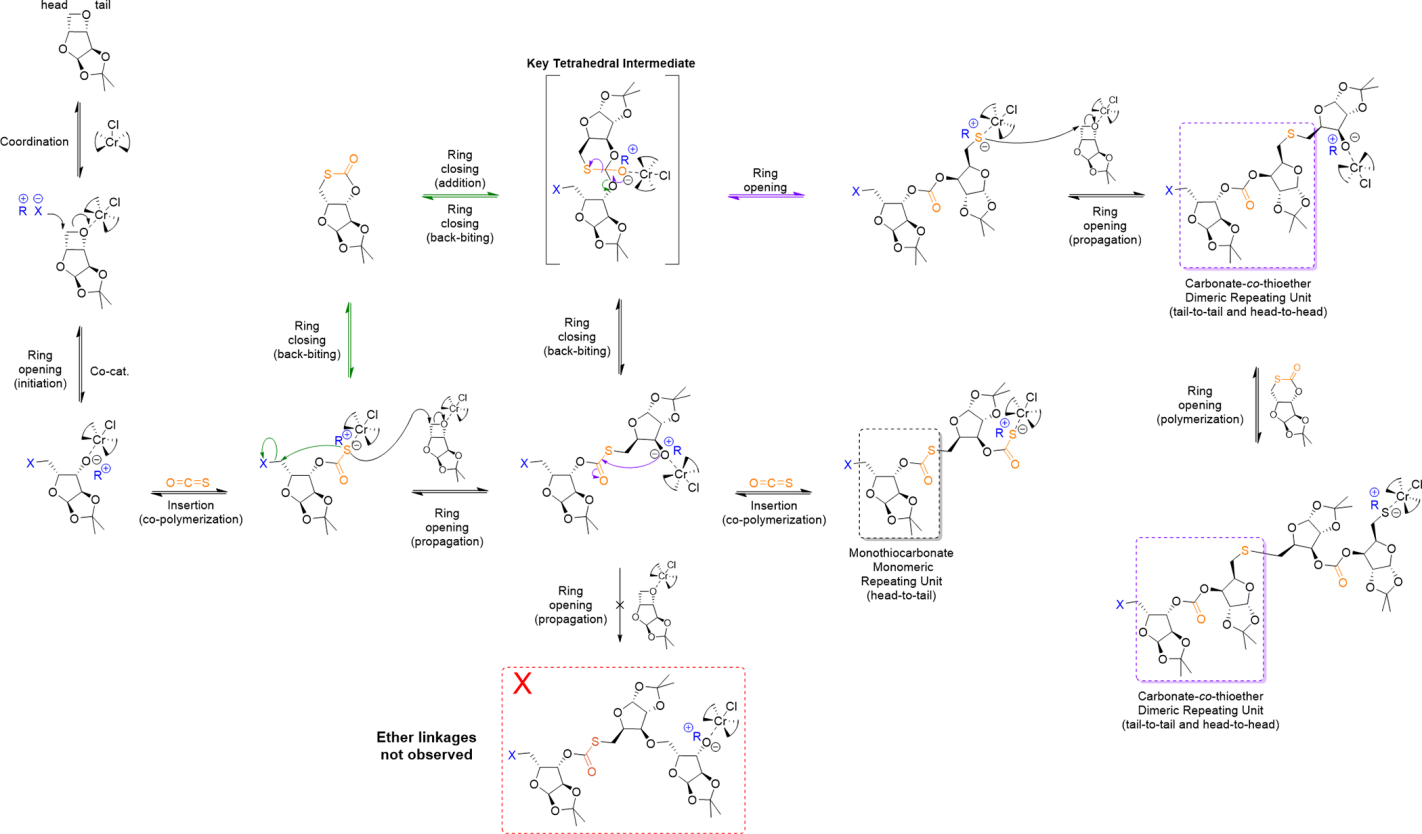
Proposed mechanism of the O/S ER during ROCOP with COS.

Figure 1 represents our proposed mechanism for the copolymerization of 3,5-anhydro-1,2-*O*-isopropylidene-α-d-xylofuranose with COS. At low temperatures,*i.e.*, 40 °C, xylose oxetane coordinates with (salen)CrCl, allowing the anion of the cocatalyst to facilitate the ring-opening initiation step, generating an alkoxide-Cr complex chain end. Next, a COS insertion process occurs to form a monothiocarbonate resulting in a sulfide-Cr complex chain end, which undergoes a S_N_2-like reaction by attack on the methylene carbon of xylose oxetane to generate an alkoxide-Cr complex. Although not observed at 40 °C, the monothiocarbonate chain end could undergo an intramolecular ring-closing elimination reaction to generate a six-membered cyclic monothiocarbonate. This alternating process, ring-opening of a xylose oxetane unit followed by COS insertion, continues to afford the poly(MTC) composed of monothiocarbonate monomeric repeating units. Matrix-assisted laser desorption ionization time-of-flight (MALDI-ToF) analysis of polymer ***7*** revealed several series of peaks (Figure S35), demonstrating the complexity and limited control during the ROCOP. Moreover, the two most abundant peak series in the MALDI-ToF spectrum indicated an alternate initiation step that involved attack upon COS followed by the oxetane to result in methylmonothiocarbonate alpha chain ends after precipitation into methanol, rather than the chloroalkyl chain end from initiation by attack on the oxetane. At higher temperatures, the compositional profile was further complicated by the COS insertion reaction being in competition with a back-biting reaction of the alkoxide at the growing chain end to the carbonyl of a monothiocarbonate center, forming a tetrahedral intermediate. Elimination from this tetrahedral intermediate has two possible pathways to proceed, which includes a favorable C–S bond cleavage to give a carbonate connection and a sulfur anion chain end *vs* a less favorable C–O bond cleavage producing a cyclic monothiocarbonate small molecule and an alkoxide chain end, respectively. With a sulfur anion at the growing chain end, preference for subsequent attack upon and ring opening of xylose oxetane locks the carbonate-*co*-thioether dimeric repeating unit into the backbone and regenerates an alkoxide chain end from which propagation continues. Addition of the alkoxide chain end to any six-membered cyclic monothiocarbonate that is produced during the reaction followed by ring-opening elimination would result in consumption of the cyclic small molecule intermediate with its conversion into carbonate-*co-*thioether dimeric repeating units.

**Figure 2:**
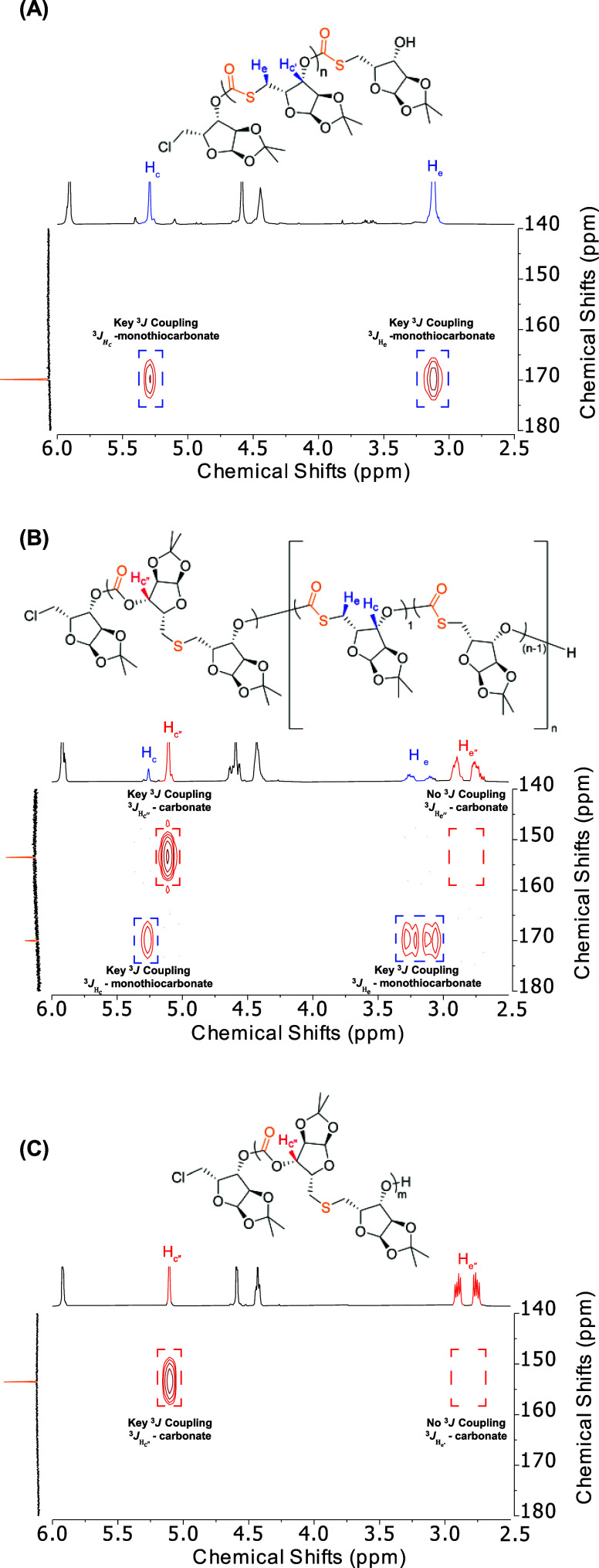
HMBC spectra of (A) poly(MTC), (B) poly[(MTC)-*co-*(CTE)], and (C) poly(CTE).

^1^H-^13^C heteronuclear multiple-bond (HMBC) 2D NMR analyses were employed to examine the structural details and the connectivity of poly(MTC), poly[(MTC)-*co*-(CTE)], and poly(CTE) to investigate our proposed mechanism. The HMBC spectrum of poly(MTC) displayed two cross peaks at (169.5, 5.29 ppm) and (169.5, 3.12 ppm) resulting from ^3^*J*_CH_ couplings between each methine proton (H_c_) and the methylene proton (H_e_) with the monothiocarbonate carbonyl carbon, respectively, indicating that the monothiocarbonate monomeric repeating units were connected regioregularly *via* head-to-tail linkages (Figure 2A). Additionally, the HMBC spectrum of poly(CTE) showed only one cross peak at (153.2, 5.11 ppm) resulting from a ^3^*J*_CH_ coupling between the methine proton (H_c′_) and the carbonate carbonyl carbon, suggesting that the carbonate and thioether dimeric repeating units were regioregularly connected tail-to-tail for the carbonate linkage and head-to-head for the thioether linkage (Figure 2C). Each poly[(MTC)-*co*-(CTE)] copolymer exhibited three cross peaks observed at (169.5, 5.29 ppm), (169.5, 3.12 ppm), and (153.2, 5.11 ppm), due to the presence of both monothiocarbonate monomeric repeating units and carbonate-*co*-thioether dimeric repeating units (Figure 2B).

Differential scanning calorimetry (DSC) and thermogravimetric analysis (TGA) were conducted to evaluate the effects of sulfur inclusion in the polymer backbone, relative to polycarbonate analogues, and also to probe effects of the nature of inclusion as monothiocarbonate *vs* combinations of monothiocarbonate and thioether functionalities (Figures S27–S34). Generally, it is established that the replacement of oxygen atoms by sulfur elevates the thermal transition temperatures of polymers compared to their non-sulfur analogues.^29,43,47−49^ Previously, we reported the *T*_g_ of poly(1,2-*O*-isopropylidene-α-d-xylofuranose carbonate)s having a molar mass of *ca*. 6–9 kDa to be 125 °C, and those polymers underwent thermal decomposition (*T*_d_) with 100% mass loss over a relatively low and narrow temperature range (200–280 °C).^20^ In this current study, the *T*_g_ values of poly(MTC) (***1*** and ***7***) were determined to be 113–115 °C, slightly lower than their polycarbonate counterpart; however, they were also *ca*. 50% lower molar mass. The elevated ROCOP temperatures that led to O/S exchange to afford the various structurally metamorphosed poly[(MTC)-*co*-(CTE)] and poly(CTE) copolymers also allowed for higher molar masses to be achieved. Each of those copolymers exhibited a *T*_g_ within the range of 133–141 °C. It is uncertain to what extent the higher *T*_g_ for those materials was due to the inclusion and nature of inclusion of sulfur *vs* the higher degrees of polymerization. However, it was confirmed that the inclusion of sulfur produced enhancements to the thermal stability, as observed by elevated decomposition temperatures for each poly(MTC), poly[(MTC)-*co*-(CTE)], and poly(CTE) *vs* the polycarbonate. Poly(MTC) underwent *ca.*70% mass loss over a temperature range of 290–320 °C. The profiles for thermal decomposition occurred at increasing temperatures as the extent of O/S exchange had occurred across the series of poly[(MTC)-*co*-(CTE)] and poly(CTE) samples, corresponding to increasing extents of dimeric carbonate and thioether repeating units (Figure S30). There were distinct differences between the poly[(MTC)-*co*-CTE)] TGA profiles that seemed to be based on effects of both temperature and [catalyst]:[cocatalyst] ratio. For a [catalyst]:[cocatalyst] ratio of 1:1, two thermal decompositions were displayed until a temperature of ≥120 °C, whereas one thermal decomposition was observed for samples prepared using a [catalyst]:[cocatalyst] ratio of 1:2 at ROCOP temperatures ≥80 °C.

## Conclusions

Through efforts to expand the scope of C1 feedstocks from commonly used CO_2_ to COS in ROCOP reactions with cyclic ethers derived from carbohydrates, we identified an exciting structural metamorphosis that occurs *in situ* during the neat ROCOP, specifically with a d-xylose-based oxetane. This process was optimized to afford a series of polymers composed of d-xylose repeating units connected *via* combinations of monothiocarbonate, carbonate, and/or thioether linkages along the backbones. Mechanistically, the dynamic *in situ* variations led to the ability to produce polymers composed primarily of monothiocarbonate monomeric repeating units (poly(MTC)), mixtures of monothiocarbonate monomeric repeating units with carbonate and thioether dimeric repeating units (poly[(MTC)-*co*-(CTE)]), or predominantly carbonate and thioether dimeric repeating units (poly(CTE)) by simply tuning the reaction conditions. In addition to introducing sulfur into the polymer backbone with variable chemical constitutions, this copolymerization addresses several challenges that had been experienced for an analogous CO_2_-based polycarbonate system,^13^ including achieving higher molar masses and reduced reaction times, while obtaining regioregularity.

Application of several spectroscopic techniques allowed for determination of the polymer compositions and structures, while thermal analyses provided information regarding the unique properties that originated from the incorporation of S, as a function of the proportions of monothiocarbonate *vs* carbonate and thioether functionalities. To investigate the effects of reaction temperature on the extent of metamorphic reorganization *in situ* during ROCOP, polymer backbone composition was studied by FT-IR, ^1^H, and ^13^C NMR spectroscopic analyses. It was determined that with increasing temperatures, monothiocarbonate monomeric repeating units were transformed to carbonate and thioether dimeric repeating units. At temperatures ≥120 °C, a doubling of cocatalyst loading also played a role in the backbone composition, leading to *ca.* quantitative formation of poly(CTE) with high regioregularity. Further investigation of these polymer samples using 2D ^1^H-^13^C HMBC indicated that the polymers synthesized at 40 °C had a head-to-tail regiochemistry, while the polymers synthesized at ≥120 °C had a tail-to-tail and head-to-head regiochemistry. These findings allowed for the proposed mechanism of the O/S exchange reaction *via* a series of inter-related pathways involving addition–elimination reactions, which accounted for each repeating unit reported. Thermal analyses of these sulfur-containing polymers highlighted the effect of sulfur inclusion in the polymer backbone.

This work provides a feasible method for synthesizing sulfur-containing sustainable polymers, with the opportunity to tune reaction conditions to access polymers composed of different backbone linkages. We expect that the fundamental study of this *in situ* structural metamorphosis and its ability to tune the polymer backbone structure will be of significant interest, especially as the field of skeletal editing of polymer backbones is growing.^50−52^ Future work for this d-xylose-based oxetane will involve ROCOP reactions with C1 feedstocks and additional four- or three-membered cyclic ethers to increase the complexity of the materials structures and properties while maintaining a simple straightforward synthetic pathway. In addition, the six-membered cyclic monothiocarbonate intermediate derived from this d-xylose-based oxetane is a synthetic target of interest. Methodologies for production, isolation, and utilization of this six-membered cyclic monothiocarbonate are being pursued.

## Supporting Information Available

Materials, experimental procedures, ^1^H and ^13^C NMR spectra, FT-IR spectra, SEC chromatograms, TGA curves, DSC curves, and MALDI-MS (PDF)
