# Supplementary material for: Structural Metamorphoses of d-Xylose Oxetane- and Carbonyl Sulfide-Based Polymers In Situ during Ring-Opening Copolymerizations
Source: J Am Chem Soc. 2023 Aug 14;145(33):18560–7. doi: 10.1021/jacs.3c05529 (PMC10863053; doi:10.1021/jacs.3c05529)
Supplement: Supplementary file 1 — ja3c05529_si_001.pdf [file ja3c05529_si_001.pdf]

Supporting Information

Structural metamorphoses of D-xylose oxetane- and carbonyl sulfide-based polymers *in-situ* during ring-opening copolymerizations

David K. Tran,[†] Ashley N. Braaksma,[†] Autumn M. Andras,[†]

Senthil K. Boopathi,[†] Donald J. Darensbourg,*[†] and Karen L. Wooley*[†,‡,§]

[†] Department of Chemistry, Texas A&M University, College Station, Texas 77843, United States

[‡] Department of Chemical Engineering, Texas A&M University, College Station, Texas 77843, United States

[§] Department of Materials Science & Engineering, Texas A&M University, College Station, Texas 77843, United States

*Corresponding author e-mail: djdarens@chem.tamu.edu

*Corresponding author e-mail: wooley@chem.tamu.edu

Materials:
Reactions were performed under a N_2 or argon atmosphere, using Schlenk and glovebox techniques. Glassware and stainless steel autoclave reactors were oven-dried at 110 °C for 24 hours prior to use. 3,5-Anhydro-1,2-*O*-isopropylidene-α-D-xylofuranose was prepared according to procedures reported from our previous work.[1] 1,5,7-Triazabicyclo[4.4.0]dec-5-ene (TBD) was purchased from TCI America and was degassed and stored in a glovebox under Ar atmosphere. (*R*,*R*)-*N,N′*-Bis(3,5-di-*tert*-butylsalicylidene)-1,2-cyclohexanediaminochromium(III) chloride was purchased from Strem Chemicals and was degassed and stored in a glovebox under Ar atmosphere. Carbonyl sulfide (COS) was purchased from Praxair. Bis(triphenylphosphine)iminium chloride (PPNCl) was purchased from Sigma-Aldrich, Co. and was recrystallized from DCM/diethyl ether. Tetrahydrofuran (THF), dichloromethane (DCM), and toluene were purified by passage through a solvent purification system (J. C. Meyer Solvent Systems, Inc., Laguna Beach, CA).

Instrumentation, Methods, and Analysis:
^1H NMR and ^{13}C NMR spectra were recorded on a Bruker Avance III™ HD 500 MHz console with an Oxford magnet, an automated tuning 5 mm $^1H/^{13}C/^{15}N$ cold probe, and a 24 position SampleCase sample changer interfaced to an UNIX computer using the VnmrJ software. Chemical shifts for ^1H NMR and ^{13}C NMR signals were referenced to the solvent resonance frequencies. The integration values for the ^1H NMR data are reported as the relative values based upon protons present within each repeat unit rather than the total protons per polymer chain, as there were no unique chain end signals to be able to calculate degree of polymerization *via* end group analysis. The ratio between monothiocarbonate monomeric repeat units to carbonate-*co*-thioether dimeric repeat units was calculated as follows:

$$\frac{\text{Monothiocarbonate monomeric repeat unit}}{\text{carbonate-}co\text{-thioether repeat unit}} = \frac{(H_e + H_{e'})}{0.50 * (H_{e''})}$$

[1] Tran, D. K.; Rashad, A. Z.; Darensbourg, D. J.; Wooley, K. L. "Sustainable Synthesis of CO_2-derived Polycarbonates from D-Xylose", *Polym. Chem.,* **2021**, *12,* 5271-5278, DOI: 10.1039/D1PY00784J.

For polymers composed of monothiocarbonate monomeric repeat units and carbonate and thioether dimeric repeat units, the summation of the integration values of H_c, $H_{c'}$, and $H_{c''}$ is equal to one proton while the summation of integration values of H_e, $H_{e'}$, and $H_{e''}$ is equal to two protons.

Fourier transform-infrared (FT-IR) spectra were recorded on an IR Prestige 21 system, equipped with a diamond attenuated total reflection (ATR) lens (Shimadzu Corp., Japan), and analyzed using IRsolution *v*. 1.40 software.

MALDI-ToF mass spectrometry analysis was performed using a Bruker Microflex MALDI-ToF mass spectrometer (Bruker Daltonics) operated using FlexControl software version 3.4. Stock solutions of the matrix *trans*-2-[3-(4-*tert*-butylphenyl)-2-methyl-2-propenylidene]malononitrile (DCTB), polymer analyte, and alkali cation sodium trifluoroacetate (NaTFA) were prepared by dissolving DCTB (25.6 mg) in chloroform ($CHCl_3$) (1 mL), polymer analyte (1.0 mg) in $CHCl_3$ (1 mL), and NaTFA (1.0 mg) in acetone (1 mL) in glass vials. In an Eppendorf tube, DCTB (2 μL), polymer analyte (1 μL), and NaTFA (1 μL) were pipette mixed, and 1 μL of the mixture was spotted onto the target section of a 384 well ground steel MALDI-ToF plate. The spot was left to evaporate to dryness before inserting the plate into the instrument. Once loaded, spectra were acquired in linear positive ion mode.

Size exclusion chromatography (SEC) eluting with THF was conducted on a Waters chromatography, Inc. (Milford, MA) system equipped with an isocratic pump model 1515, a differential refractometer model 2414, and a three-column set including a guard column (PLgel 5 μm, 50 × 7.5 mm) and two Styragel columns (PLgel 5 μm Mixed C, 500 Å, and 104 Å, 300 × 7.5 mm columns). The system was operated at 40 °C with a flow rate of 1 mL/min. Data were analyzed using Breeze *v*. 6.20 software from Waters Chromatography, Inc. (Milford, MA). Molar masses were determined relative to polystyrene standards (300–467,000 Da) purchased from Polymer Laboratories, Inc. (Amherst, MA). Polymer solutions were prepared at a concentration of *ca*. 3 mg/mL with 0.05 vol% toluene as flow rate marker and an injection volume of 200 μL was used.

Glass transition temperatures (T_g) were measured by differential scanning calorimetry (DSC) on a Mettler-Toledo DSC3/700/1190 (Mettler-Toledo, Inc., Columbus, OH) under a nitrogen gas atmosphere. Measurements were performed with heating and cooling rates of 10 °C/min, and three heating and cooling cycles were conducted. Measurements were analyzed using Mettler-Toledo STAR^e *v*. 15.00a software. The T_g was taken as the midpoint of the inflection tangent of the third heating scan.

Thermogravimetric analysis (TGA) was performed under N_2 atmosphere using a Mettler-Toledo TGA2/1100/464, with a heating rate of 10 °C/min. Data were analyzed using Mettler-Toledo STAR^e *v*. 15.00a software.

Synthetic Protocol:

General synthetic procedure of copolymerization of 3,5-anhydro-1,2-*O*-isopropylidene-α-D-xylofuranose (xylose oxetane) and COS:

Under an argon atmosphere in a glovebox, (salen)CrCl, cocatalyst, and 3,5-anhydro-1,2-*O*-isopropylidene-α-D-xylofuranose were added to a 10 mL stainless steel Parr autoclave reactor that had previously dried overnight at 110 °C and was then sealed for transfer to a chemical fume hood. **CAUTION!!!: COS is a highly toxic gas and should always be handled by experienced personnel in a well-ventilated hood.** The reactor was pressurized with COS and placed in a preheated oil bath and stirred for 4 hours. The reactor was cooled in an ice bath for 10 min and pressure was slowly released. A small aliquot was taken for ^{1}H NMR analysis, and the resulting solid was dissolved with a minimal amount of THF, precipitated into an acidic methanol solution, and purified by precipitation from THF into methanol thrice to afford a white solid.

Polymers ***1*** – ***6*** were synthesized using xylose oxetane on a scale of 625 mg (3.63 mmol) and a ratio of 1 : 1 : 150 : 300 for (salen)CrCl : PPNCl cocatalyst : xylose oxetane : COS, at varying temperatures from 40 to 140 °C. Polymers ***7*** – ***12*** were synthesized using xylose oxetane on a scale of 625 mg (3.63 mmol) and a ratio of 1 : 2 : 150 : 300 for (salen)CrCl : PPNCl cocatalyst : xylose oxetane : COS, at varying temperatures from 40 to 140 °C. Polymers ***13*** – ***17*** were synthesized using xylose oxetane on a scale of 625 mg (3.63 mmol) and a ratio of 1 : 2 : 150 : 300 for (salen)CrCl : TBD cocatalyst : xylose oxetane : COS, at varying temperatures from 100 to 140 °C.

Polymer ***1***: The copolymerization was conducted at 40 °C and afforded ***1*** with a monothiocarbonate backbone. After precipitation in methanol thrice, a white solid was obtained (120 mg, 19.2% yield).

$M_{n\ SEC,THF}$ = 3.4 kDa, $Đ$ = 1.17. ^{1}H NMR (500 MHz, $CDCl_3$, ppm) δ 5.91 (H_a, m, 1H), 5.41 – 5.19 (H_c, m, 1H), 4.59 (H_b, m, 1H), 4.44 (H_d, m, 1H), 3.17 – 3.07 (H_e, m, 2H), 1.51 (m, 3H), 1.31 (m, 3H). ^{13}C NMR (126 MHz, $CDCl_3$, ppm) δ 169.9 – 169.8, 112.7 – 112.5, 105.2 – 104.8, 83.3 – 83.0, 80.1, 79.4, 39.3, 29.0, 28.6, 26.8 – 26.7. FT-IR(ATR): 3070

– 2785, 1712, 1442, 1381, 1219, 1134, 1080, 1018, 871, 810, 763, 725, 663 cm^{-1}. T_g = 115 °C. TGA in N_2: 25–253 ºC, 5% mass loss; 291–320 ºC, 68% mass loss; 320-500, 12%; 15% mass remaining above 500 ºC.

Polymer ***2***: The copolymerization was conducted at 60 °C and afforded ***2*** with a monothiocarbonate, carbonate, and thioether backbone. After precipitation in methanol thrice, a white solid was obtained (173 mg, 27.7% yield).

$M_{n\ SEC,THF}$ = 13.1 kDa, $Đ$ = 1.06. ^{1}H NMR (500 MHz, $CDCl_3$, ppm) δ 5.97 – 5.82 ($H_{a,a',a''}$, m, 1H), 5.36 – 5.28 (H_c, m, 0.20H), 5.28 – 5.22 ($H_{c'}$, m, 0.14H), 5.16 – 5.06 ($H_{c''}$, m, 0.62H) 4.72 – 4.52 ($H_{b,b',b''}$, m, 1H), 4.48 – 4.35 ($H_{d,d',d''}$, m, 1H), 3.35 – 3.18 ($H_{e'}$, m, 0.3H), 3,18 – 3.05 (H_e, m, 0.6H), 2.94 – 2.66 ($H_{e''}$, m, 1.10H), 1.54 – 1.47 (m, 3H), 1.39 – 1.23 (m, 3H). ^{13}C NMR (126 MHz, $CDCl_3$, ppm) δ 169.9, 153.5 – 153.4, 112.5, 112.4, 104.9, 83.4, 83.1, 80.5, 79.4, 79.1, 77.9, 30.0, 29.0, 26.8, 26.3. FT-IR(ATR): 3070 – 2785, 1751, 1721, 1373, 1257, 1219, 1141, 1080, 1018, 856, 786, 671 cm^{-1}. T_g = 133 °C. TGA in N_2: 25-185 °C, 3% mass loss; 185 -258 °C, 19% mass loss; 258 – 327 °C, 47% mass loss; 327 – 500 °C, 16% mass loss; 15% mass remaining above 500 °C.

Polymer ***3***: The copolymerization was conducted at 80 °C and afforded ***3*** with a monothiocarbonate, carbonate, and thioether backbone. After precipitation in methanol thrice, a white solid was obtained (138 mg, 22.1% yield).

$M_{n\ SEC,THF}$ = 13.8 kDa, $Đ$ = 1.09. ^{1}H NMR (500 MHz, $CDCl_3$, ppm) δ 5.91 ($H_{a,a',a''}$, m, 1H), 5.32 – 5.28 (H_c, m, 0.13H), 5.28 – 5.24 ($H_{c'}$, m, 0.16H), 5.15 – 5.04 ($H_{c''}$, m, 0.66H) 4.69 – 4.54 ($H_{b,b',b''}$, m, 1H), 4.51 – 4.34 ($H_{d,d',d''}$, m, 1H), 3.32 – 3.19 ($H_{e'}$, m, 0.4H), 3.19 – 3.05 (H_e, m, 0.50H), 2.99 – 2.64 ($H_{e''}$, m, 1.1H), 1.55 – 1.45 (m, 3H), 1.38 – 1.26 (m, 3H). ^{13}C NMR (126 MHz, $CDCl_3$, ppm) δ 169.9, 153.5 – 153.4, 112.6 – 112.4, 104.9, 83.3, 83.1, 80.5, 79.5, 79.1,77.9, 30.0, 29.0, 26.8, 26.3. FT-IR(ATR): 3093 – 2823, 1751, 1721, 1373, 1249, 1219, 1141, 1080, 1010, 864, 786, 671 cm^{-1}. T_g = 134 °C. TGA in N_2: 25-176 °C, 4% mass loss; 176 -273 °C, 27% mass loss; 273 – 327 °C, 38% mass loss; 327 – 500 °C, 16% mass loss; 15% mass remaining above 500 °C.

Polymer ***4***: The copolymerization was conducted at 100 °C and afforded ***4*** with a monothiocarbonate, carbonate, and thioether backbone. After precipitation in methanol thrice, a white solid was obtained (179 mg, 28.6% yield).

$M_{n\ SEC,THF}$ = 23.2 kDa, *Đ* = 1.17. ^{1}H NMR (500 MHz, $CDCl_3$, ppm) δ 5.92 ($H_{a,a',a''}$, m, 1H), 5.31 – 5.28 (H_c, m, 0.02H), 5.28 – 5.24 ($H_{c'}$, m, 0.10H), 5.13 – 5.05 ($H_{c''}$, m, 0.84H), 4.66 – 4.54 ($H_{b,b',b''}$, m, 1H), 4.43 ($H_{d,d',d''}$, m, 1H), 3.33 – 3.18($H_{e'}$, m, 0.22H), 3.18 – 3.04 (H_e, m, 0.15H), 2.96 – 2.67 ($H_{e''}$, m, 1.6H), 1.55 – 1.47 (m, 3H), 1.31 (m, 3H). ^{13}C NMR (126 MHz, $CDCl_3$, ppm) δ 170.0, 153.5, 112.6 – 112.3, 104.9, 83.1, 80.5, 79.4, 79.1, 68.1, 30.0, 26.8, 26.7, 26.3. FT-IR(ATR): 3055 – 2831, 1751, 1712, 1381, 1257, 1211, 1157, 1080, 1010, 918 – 810, 786 cm^{-1}. T_g = 134 °C. TGA in N_2: 25-222 °C, 2% mass loss; 220 -328 °C, 66% mass loss; 328 – 500 °C, 12% mass loss; 20% mass remaining above 500 °C.

Polymer ***5***: The copolymerization was conducted at 120 °C and afforded ***5*** with a monothiocarbonate, carbonate, and thioether backbone. After precipitation in methanol thrice, a white solid was obtained (235 mg, 37.6% yield).

$M_{n\ SEC,THF}$ = 23.5 kDa, *Đ* = 1.16. ^{1}H NMR (500 MHz, $CDCl_3$, ppm) δ 5.92 ($H_{a,a',a''}$, m, 1H), 5.33 – 5.28 (H_c, m, 0.02H), 5.28 – 5.24 ($H_{c'}$, m, 0.10H), 5.15 – 5.05 ($H_{c''}$, m, 0.85H), 4.68 – 4.53 ($H_{b,b',b''}$, m, 1H), 4.43 ($H_{d,d',d''}$, m, 1H),3.31 – 3.18 ($H_{e'}$, m, 0.22H), 3.18 – 3.06 (H_e, m, 0.16H), 2.94 – 2.64 ($H_{e''}$, m, 1.60H), 1.51 (m, 3H), 1.31 (m, 3H). ^{13}C NMR (126 MHz, $CDCl_3$, ppm) δ 170.0, 153.5 – 153.4, 112.6 – 112.3, 104.9, 83.1, 83.0, 80.5, 80.0, 79.6, 79.4, 79.1, 68.1, 30.0, 26.8, 26.7, 26.3. FT-IR(ATR): 3100 – 2800, 1751, 1721, 1373, 1249, 1219, 1157, 1080, 1010, 933 – 802, 786 cm^{-1}. T_g = 134 °C. TGA in N_2: 25-222 °C, 3% mass loss; 222 – 335 °C, 63% mass loss; 328 – 500 °C, 14% mass loss; 20% mass remaining above 500 °C.

Polymer ***6***: The copolymerization was conducted at 140 °C and afforded ***6*** with a monothiocarbonate, carbonate, and thioether backbone. After precipitation in methanol thrice, a white solid was obtained (222 mg, 35.5% yield).

$M_{n\ SEC,THF}$ = 23.5 kDa, $Đ$ = 1.16. ^{1}H NMR (500 MHz, $CDCl_3$, ppm) δ 5.92 ($H_{a,a',a''}$, m, 1H), 5.31 – 5.28 (H_c, m, 0.01H), 5.28 – 5.24 ($H_{c'}$, m, 0.08H), 5.15 – 5.05 ($H_{c''}$, m, 0.9H), 4.67 – 4.54 ($H_{b,b',b''}$, m, 1H), 4.43 ($H_{d,d',d''}$, m, 1H), 3.32 – 3.18 ($H_{e'}$, m, 0.17H), 3.18 – 3.04 (H_e, m, 0.11H), 2.95 – 2.67 ($H_{e''}$, m, 1.71H), 1.51 (m, 3H), 1.31 (m, 3H). ^{13}C NMR (126 MHz, $CDCl_3$, ppm) δ 170.0, 153.5 – 153.4, 112.6, 112.4, 112.4, 112.3, 104.9, 83.1, 80.5, 80.0, 79.1, 68.1, 30.0, 26.8, 26.3, 25.7. FT-IR(ATR): 3100 – 2831, 1751, 1381, 1249, 1219, 1165, 1080, 1010, 933 – 802, 779 cm^{-1}. T_g = 134 °C. TGA in N_2: 25-185 °C, 4% mass loss; 185 – 274 °C, 22% mass loss; 274 – 330 °C, 43% mass loss; 330 – 500 °C, 20% mass loss; 11% mass remaining above 500 °C.

Polymer ***7***: The copolymerization was conducted at 40 °C and afforded ***7*** with a monothiocarbonate backbone. After precipitation in methanol thrice, a white solid was obtained (150 mg, 24.0% yield).

$M_{n\ SEC,THF}$ = 3.8 kDa, $Đ$ = 1.10. ^{1}H NMR (500 MHz, $CDCl_3$, ppm) δ 5.91 (H_a, m, 1H), 5.41 – 5.19 (H_c, m, 1H), 4.59 (H_b, m, 1H), 4.44 (H_d, m, 1H), 3.11 – 3.07 (H_e, m, 2H), 1.51 (m, 3H), 1.31 (m, 3H). ^{13}C NMR (126 MHz, $CDCl_3$, ppm) δ 169.9 – 169.8, 112.7 – 112.5, 105.2 – 104.8, 83.3 – 83.0, 80.1, 79.4, 39.3, 29.0, 28.6, 26.8 – 26.7. FT-IR(ATR): 3070 – 2785, 1712, 1442, 1381, 1219, 1134, 1080, 1018, 871, 810, 763, 725, 663 cm^{-1}. T_g = 113 °C. TGA in N_2: 25–253 ºC, 5% mass loss; 291–320 ºC, 68% mass loss; 320-500, 12%; 15% mass remaining above 500 ºC.

Polymer ***8***: The copolymerization was conducted at 60 °C and afforded ***8*** with a monothiocarbonate, carbonate, and thioether backbone. After precipitation in methanol thrice, a white solid was obtained (265 mg, 42.4% yield).

$M_{n\ SEC,THF}$ = 11.7 kDa, $Đ$ = 1.10. ^{1}H NMR (500 MHz, $CDCl_3$, ppm) δ 5.92 ($H_{a,a',a''}$, m, 1H), 5.33 – 5.28 (H_c, m, 0.20H), 5.28 – 5.22 ($H_{c'}$, m, 0.20H), 5.14 – 5.05 ($H_{c''}$, m, 0.57H), 4.67 – 4.52 ($H_{b,b',b''}$, m, 1H), 4.49 – 4.35 ($H_{d,d',d''}$, m, 1H), 3.33 – 3.18 ($H_{e'}$,m, 0.36H), 3.18 – 3.04 (H_e, m, 0.65H), 2.96 – 2.67 ($H_{e''}$, m, 0.94H), 1.51 (m, 3H), 1.31 (m, 3H). ^{13}C NMR (126 MHz, $CDCl_3$, ppm) δ 169.9, 153.5 – 153.4, 112.5 – 112.4, 104.8, 83.4, 83.1, 79.4, 79.2,

77.9, 30.1, 29.1, 26.8, 26.3. FT-IR(ATR): 3109 – 2816, 1751, 1381, 1257, 1219, 1134, 1080, 1018, 933 – 825, 663 cm^{-1}. T_g = 139 °C. TGA in N_2: 25 – 185 °C, 4% mass loss; 186 – 274 °C, 20% mass loss; 274 – 336 °C, 46% mass loss; 336 – 500 °C, 12% mass loss; 18% mass remaining above 500 °C.

Polymer ***9***: The copolymerization was conducted at 80 °C and afforded ***9*** with a monothiocarbonate, carbonate, and thioether backbone. After precipitation in methanol thrice, a white solid was obtained (246 mg, 39.4% yield).

$M_{n\ SEC,THF}$ = 12.6 kDa, *Đ* = 1.06. ^{1}H NMR (500 MHz, $CDCl_3$, ppm) δ 5.91 ($H_{a,a',a''}$, m, 1H), 5.32 – 5.28 (H_c, m, 0.07H), 5.28 – 5.20 ($H_{c'}$, m, 0.17H), 5.14 – 5.04 ($H_{c''}$, m, 0.72H), 4.70 – 4.54 ($H_{b,b',b''}$, m, 1H), 4.41 ($H_{d,d',d''}$, m, 1H), 3.33 – 3.17 ($H_{e'}$, m, 0.46H), 3.17 – 3.04 (H_e, m, 0.37H), 2.96 – 2.65 ($H_{e''}$, m, 1.13H), 1.54 – 1.47 (m, 3H), 1.35 – 1.28 (m, 3H). ^{13}C NMR (126 MHz, $CDCl_3$, ppm) δ 169.9, 153.5, 153.4, 112.6, 112.4, 112.3, 104.9, 83.3, 83.1, 80.5, 79.1, 30.1, 26.8, 26.3. FT-IR(ATR): 3086 – 2831, 1751, 1721, 1373, 1249, 1219, 1149, 1080, 1018, 864 cm^{-1}. T_g = 141 °C. TGA in N_2: 25 – 220 °C, 2% mass loss; 220 – 340 °C, 72% mass loss; 240 – 500 °C, 8% mass loss; 18% mass remaining above 500 °C.

Polymer ***10***: The copolymerization was conducted at 100 °C and afforded ***10*** with a monothiocarbonate, carbonate, and thioether backbone. After precipitation in methanol thrice, a white solid was obtained (266 mg, 42.6% yield).

$M_{n\ SEC,THF}$ = 15.5 kDa, *Đ* = 1.14. ^{1}H NMR (500 MHz, $CDCl_3$, ppm) δ 5.91 ($H_{a,a',a''}$, m, 1H), 5.31 – 5.28 (H_c, m, 0.02H), 5.28 – 5.22 ($H_{c'}$, m, 0.14H), 5.15 – 5.04 ($H_{c''}$, m, 0.81H), 4.67 – 4.53 ($H_{b,b',b''}$, m, 1H), 4.48 – 4.34 ($H_{d,d',d''}$, m, 1H), 3.32 – 3.17 ($H_{e'}$, m, 0.27H), 3.17 – 3.04 (H_e, m, 0.22H), 2.95 – 2.66 ($H_{e''}$, m, 1.51H), 1.51 (m, 3H), 1.31 (m, 3H). ^{13}C NMR (126 MHz, $CDCl_3$, ppm) δ 170.0 – 169.9, 153.5 – 153.4, 112.6, 112.4, 112.4, 112.3, 104.9, 83.3 – 83.0, 80.5, 80.0, 79.6, 79.4, 79.1, 78.2, 30.2 – 30.0, 28.6, 26.8, 26.3, 26.2. FT-IR(ATR): 3055 – 2846, 1751,1712, 1373, 1249, 1211, 1157, 1080, 1018, 864, 786 cm^{-1}.

T_g = 138 °C. TGA in N_2: 25 – 220 °C, 2% mass loss; 220 – 340 °C, 70% mass loss; 340 – 500 °C, 8% mass loss; 20% mass remaining above 500 °C.

Polymer ***11***: The copolymerization was conducted at 120 °C and afforded ***11*** with a carbonate and thioether backbone. After precipitation in methanol thrice, a white solid was obtained (235 mg, 37.6% yield).

$M_{n\ SEC,THF}$ = 23.5 kDa, *Đ* = 1.16. ^{1}H NMR (500 MHz, $CDCl_3$, ppm) δ 5.92 ($H_{a''}$, d, *J* = 3.8 Hz, 1H), 5.11 ($H_{c''}$, m, 1H), 4.59 ($H_{b''}$, m, 1H), 4.43 ($H_{d''}$, ddd, *J* = 7.1,7.1, 2.9 Hz, 1H), 2.95 – 2.85 ($H_{e''_1}$, dd, *J* = 13.7, 7.1 Hz, 1H), 2.80 – 2.71 ($H_{e''_2}$, dd, *J* = 13.7, 7.1 Hz, 1H), 1.52 (s, 3H), 1.32 (s, 3H). ^{13}C NMR (126 MHz, $CDCl_3$, ppm) δ 153.5, 112.6, 112.4, 104.9, 83.1, 80.5, 79.1, 30.0, 26.8, 26.3. FT-IR(ATR): 3100 – 2839, 1751, 1373, 1249, 1219, 1157, 1072, 1018, 933 – 810, 786 cm^{-1}. T_g = 135 °C. TGA in N_2: 25 – 220 °C, 2% mass loss; 220 – 340 °C, 68% mass loss; 340 – 500 °C, 8% mass loss; 22% mass remaining above 500 °C.

Polymer ***12***: The copolymerization was conducted at 140 °C and afforded ***12*** with a carbonate and thioether backbone. After precipitation in methanol thrice, a white solid was obtained (302 mg, 48.3% yield).

$M_{n\ SEC,THF}$ = 27.9 kDa, *Đ* = 1.17. ^{1}H NMR (500 MHz, $CDCl_3$, ppm) δ 5.92 ($H_{a''}$, d, *J* = 3.8 Hz, 1H), 5.11 ($H_{c''}$, m, 1H), 4.59 ($H_{b''}$, m, 1H), 4.43 ($H_{d''}$, ddd, *J* = 7.1,7.1, 2.9 Hz, 1H), 2.95 – 2.85 ($H_{e''_1}$, dd, *J* = 13.7, 7.1 Hz, 1H), 2.80 – 2.71 ($H_{e''_2}$, dd, *J* = 13.7, 7.1 Hz, 1H), 1.52 (s, 3H), 1.32 (s, 3H). ^{13}C NMR (126 MHz, $CDCl_3$, ppm) δ 153.5, 112.4, 104.9, 83.1, 80.5, 79.1, 68.1, 30.0, 26.8, 26.3, 25.7. FT-IR(ATR): 3086 – 2808, 1751, 1373, 1249, 1219, 1165, 1080, 1018, 933 – 802, 771 cm^{-1}. T_g = 137 °C. TGA in N_2: 25 – 210 °C, 2% mass loss; 210 – 340 °C, 68% mass loss; 340 – 500 °C, 8% mass loss; 22% mass remaining above 500 °C.

Polymer ***13***: The copolymerization was conducted at 100 °C and afforded ***13*** with a monothiocarbonate, carbonate, and thioether backbone. After precipitation in methanol thrice, a white solid was obtained (458 mg, 73.2% yield).

$M_{n\ SEC,THF}$ = 13.1 kDa, $Đ$ = 1.28. ^{1}H NMR (500 MHz, $CDCl_3$, ppm) δ 5.92 ($H_{a,a',a''}$, m, 1H), 5.31 – 5.28 (H_c, m, 0.02H), 5.28 – 5.22 ($H_{c'}$, m, 0.10H), 5.15 – 5.07 ($H_{c''}$, m, 0.84H), 4.67 – 4.55 ($H_{b,b',b''}$, m, 1H), 4.48 – 4.35 ($H_{d,d',d''}$, m, 1H), 3.32 – 3.17 ($H_{e'}$, m, 0.19H), 3.17 – 3.04 (H_e, m, 0.16H), 2.96 – 2.67 ($H_{e''}$, m, 1.64H), 1.51 (m, 3H), 1.31 (m, 3H). ^{13}C NMR (126 MHz, $CDCl_3$, ppm) δ 170.0, 153.5 – 153.4, 112.6 – 112.3, 104.9 – 104.8, 83.3 – 83.0, 80.5 – 80.0, 79.6 – 79.1, 78.2, 68.1, 30.2 – 30.0, 28.6, 26.8 – 26.7, 26.3 – 26.2, 25.7. FT-IR(ATR): 3100 – 2816, 1751,1712, 1373, 1249, 1219, 1165, 1080, 1018, 933 – 802, 779 cm^{-1}. T_g = 134 °C. TGA in N_2: 25 – 281 °C, 10% mass loss; 281 – 340 °C, 72% mass loss; 340 – 500 °C, 13% mass loss; 5% mass remaining above 500 °C.

Polymer ***14***: The copolymerization was conducted at 110 °C and afforded ***14*** with a monothiocarbonate, carbonate, and thioether backbone. After precipitation in methanol thrice, a white solid was obtained (372 mg, 59.5% yield).

$M_{n\ SEC,THF}$ = 14.3 kDa, $Đ$ = 1.28. ^{1}H NMR (500 MHz, $CDCl_3$, ppm) δ 5.91 ($H_{a,a',a''}$, m, 1H), 5.31 – 5.28 (H_c, m, 0.02H), 5.28 – 5.22 ($H_{c'}$, m, 0.10H), 5.15 – 5.07 ($H_{c''}$, m, 0.87H), 4.67 – 4.55 ($H_{b,b',b''}$, m, 1H), 4.48 – 4.35 ($H_{d,d',d''}$, m, 1H), 3.32 – 3.17 ($H_{e'}$, m, 0.14H), 3.17 – 3.04 (H_e, m, 0.14H), 2.97 – 2.68 ($H_{e''}$, m, 1.72H), 1.51 (m, 3H), 1.31 (m, 3H). ^{13}C NMR (126 MHz, $CDCl_3$, ppm) δ 170.0, 153.5 – 153.5, 112.6 – 112.3, 104.9, 104.8, 83.3 – 83.0, 80.5, 80.5, 80.0, 79.6 – 79.1, 78.2, 68.1, 30.2 – 30.0, 28.6, 26.8 – 26.8, 26.3 – 26.2. FT-IR(ATR): 3070 – 2823, 1751, 1712, 1373, 1249, 1219, 1157, 1080, 1018, 933 – 802, 779 cm^{-1}. T_g = 132 °C. TGA in N_2: 25 – 290 °C, 8% mass loss; 290 – 350 °C, 76% mass loss; 340 – 500 °C, 6% mass loss; 10% mass remaining above 500 °C.

Polymer ***15***: The copolymerization was conducted at 120 °C and afforded ***12*** with a monothiocarbonate, carbonate, and thioether backbone. After precipitation in methanol thrice, a white solid was obtained (348 mg, 55.7% yield).

$M_{n\ SEC,THF}$ = 14.3 kDa, $Đ$ = 1.35. ^{1}H NMR (500 MHz, $CDCl_3$, ppm) δ 5.92 ($H_{a,a',a''}$, m, 1H), 5.31 – 5.28 (H_c, m, 0.01H), 5.28 – 5.22 ($H_{c'}$, m, 0.09H), 5.15 – 5.07 ($H_{c''}$, m, 0.89H), 4.67 – 4.55 ($H_{b,b',b''}$, m, 1H), 4.48 – 4.35 ($H_{d,d',d''}$, m, 1H), 3.32 – 3.17 ($H_{e'}$, m, 0.12H), 3.17 – 3.04 (H_e, m, 0.12H),2.95 – 2.66 (m, 1.75H), 1.51 (m, 3H), 1.31 (m, 3H). ^{13}C NMR (126 MHz, $CDCl_3$, ppm) δ 169.8, 153.3 – 153.3, 112.4 – 112.2, 104.7, 83.2, 83.0, 82.8, 80.3, 79.9 – 79.0, 78.0, 67.9, 30.0, 29.9, 26.7 – 26.6, 26.2 – 26.1. FT-IR(ATR): 3078 – 2800, 1751, 1712, 1373, 1249, 1219, 1157, 1080, 1018, 933 – 802, 779 cm^{-1}. T_g = 134 °C. TGA in N_2: 25 – 300 °C, 6% mass loss; 300 – 350 °C, 76% mass loss; 350 – 500 °C, 5% mass loss; 13% mass remaining above 500 °C.

Polymer ***16***: The copolymerization was conducted at 130 °C and afforded ***16*** with a carbonate and thioether backbone. After precipitation in methanol thrice, a white solid was obtained (403 mg, 64.5% yield).

$M_{n\ SEC,THF}$ = 13.2 kDa, $Đ$ = 1.30. ^{1}H NMR (500 MHz, $CDCl_3$, ppm) δ 5.92 ($H_{a''}$, d, J = 3.8 Hz, 1H), 5.11 ($H_{c''}$, m, 1H), 4.59 ($H_{b''}$, m, 1H), 4.43 ($H_{d''}$, ddd, J = 7.1, 7.1, 2.9 Hz, 1H), 2.94 – 2.85 ($H_{e''_1}$, dd, J = 13.7, 7.1 Hz, 1H), 2.80 – 2.71 ($H_{e''_2}$, dd, J = 13.7, 7.1 Hz, 1H), 1.52 (s, 3H), 1.32 (s, 3H). ^{13}C NMR (126 MHz, $CDCl_3$, ppm) δ 153.5, 112.6, 112.4, 104.9, 83.1, 80.5, 79.1, 68.1, 30.0, 26.8, 26.3, 25.7. FT-IR(ATR): 3100 – 2785, 1751, 1381, 1249, 1219, 1165, 1080, 1010, 933 – 802, 779 cm^{-1}. T_g = 132 °C. TGA in N_2: 25 – 300 °C, 6% mass loss; 300 – 350 °C, 78% mass loss; 350 – 500 °C, 5% mass loss; 11% mass remaining above 500 °C.

Polymer ***17***: The copolymerization was conducted at 140 °C and afforded ***17*** with a carbonate and thioether backbone. After precipitation in methanol thrice, a white solid was obtained (458 mg, 73.3% yield).

$M_{n\ SEC,THF}$ = 14.3 kDa, $Đ$ = 1.30. ^{1}H NMR (500 MHz, $CDCl_3$, ppm) δ 5.92 ($H_{a''}$, d, J = 3.8 Hz, 1H), 5.11 ($H_{c''}$, m, 1H), 4.59 ($H_{b''}$, m, 1H), 4.43 ($H_{d''}$, ddd, J = 7.1, 7.1, 2.9 Hz, 1H), 2.94 – 2.85 ($H_{e''_1}$, dd, J = 13.7, 7.1 Hz, 1H), 2.80 – 2.71 ($H_{e''_2}$, dd, J = 13.7, 7.1 Hz, 1H), 1.52 (s, 3H), 1.32 (s, 3H). ^{13}C NMR (126 MHz, $CDCl_3$, ppm) δ 153.5, 112.6, 112.4, 104.9,

83.1, 80.5, 79.1, 68.1, 30.0, 26.8, 26.3, 25.7. FT-IR(ATR): 3100 – 2785, 1751, 1381, 1249, 1219, 1165, 1080, 1010, 933 – 802, 779 cm^{-1}. T_g = 132 °C. TGA in N_2: 25 – 300 °C, 6% mass loss; 300 – 360 °C, 79% mass loss; 360 – 500 °C, 5% mass loss; 10% mass remaining above 500 °C.

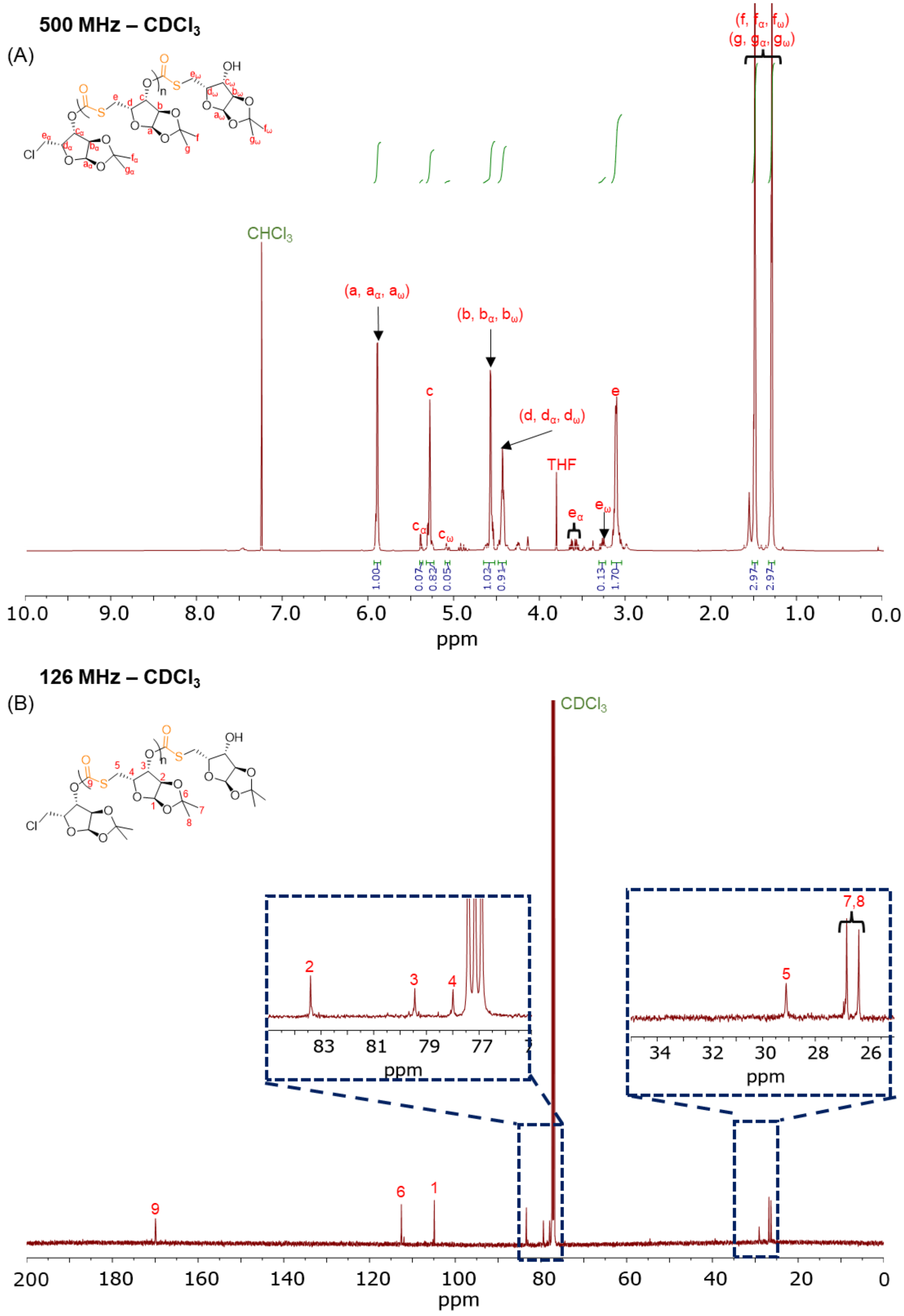

Figure S1: (A) ^{1}H NMR (500 MHz, $CDCl_3$) and (B) ^{13}C NMR (126 MHz, $CDCl_3$) spectrum of polymer ***1***.

500 MHz – $CDCl_3$

(A)

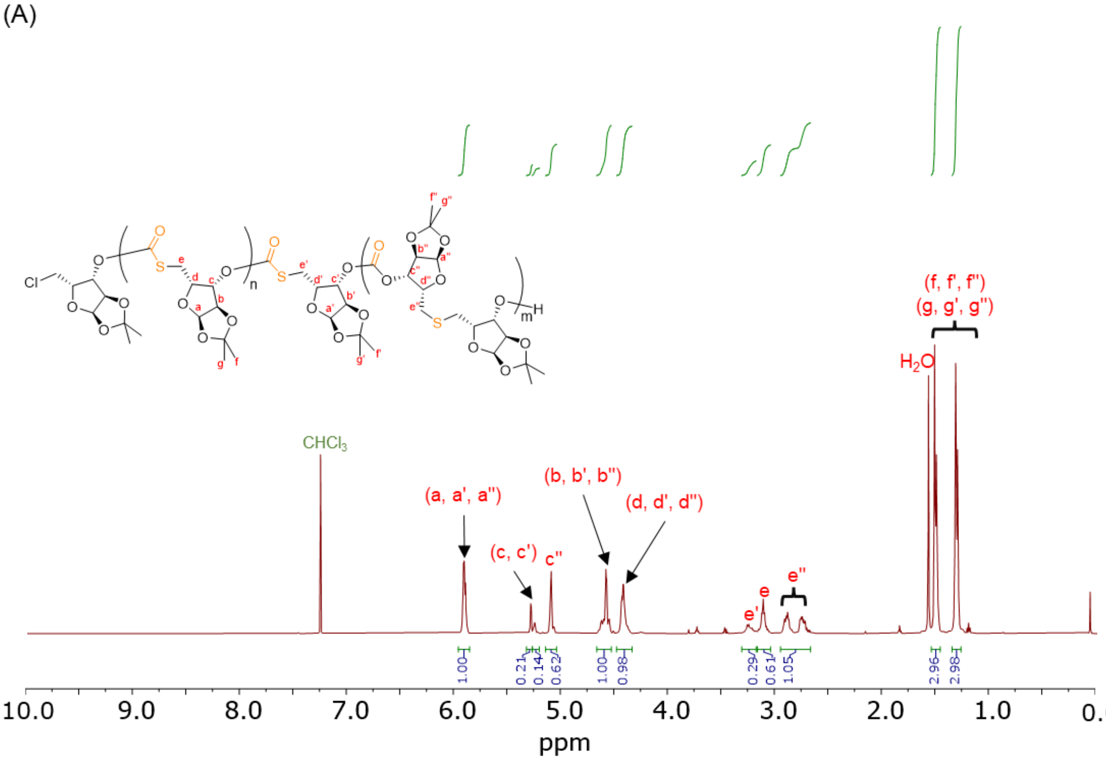

126 MHz – $CDCl_3$

(B)

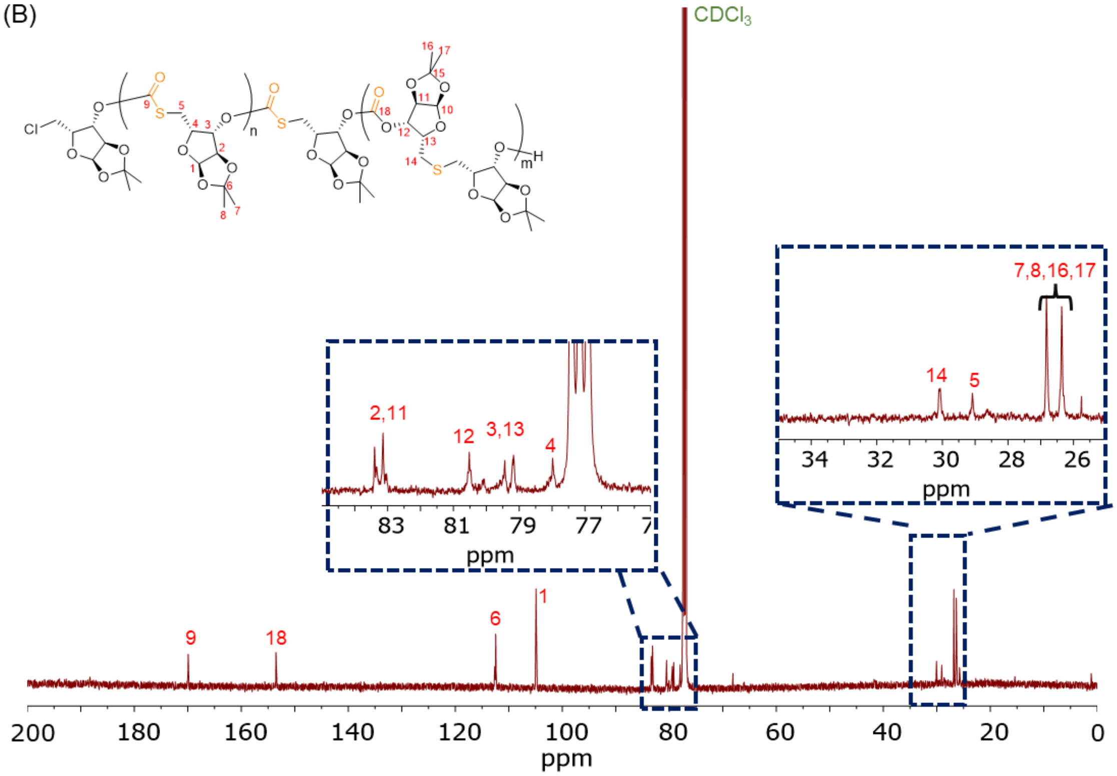

Figure S2: (A) ^{1}H NMR (500 MHz, $CDCl_3$) and (B) ^{13}C NMR (126 MHz, $CDCl_3$) spectrum of polymer ***2***.

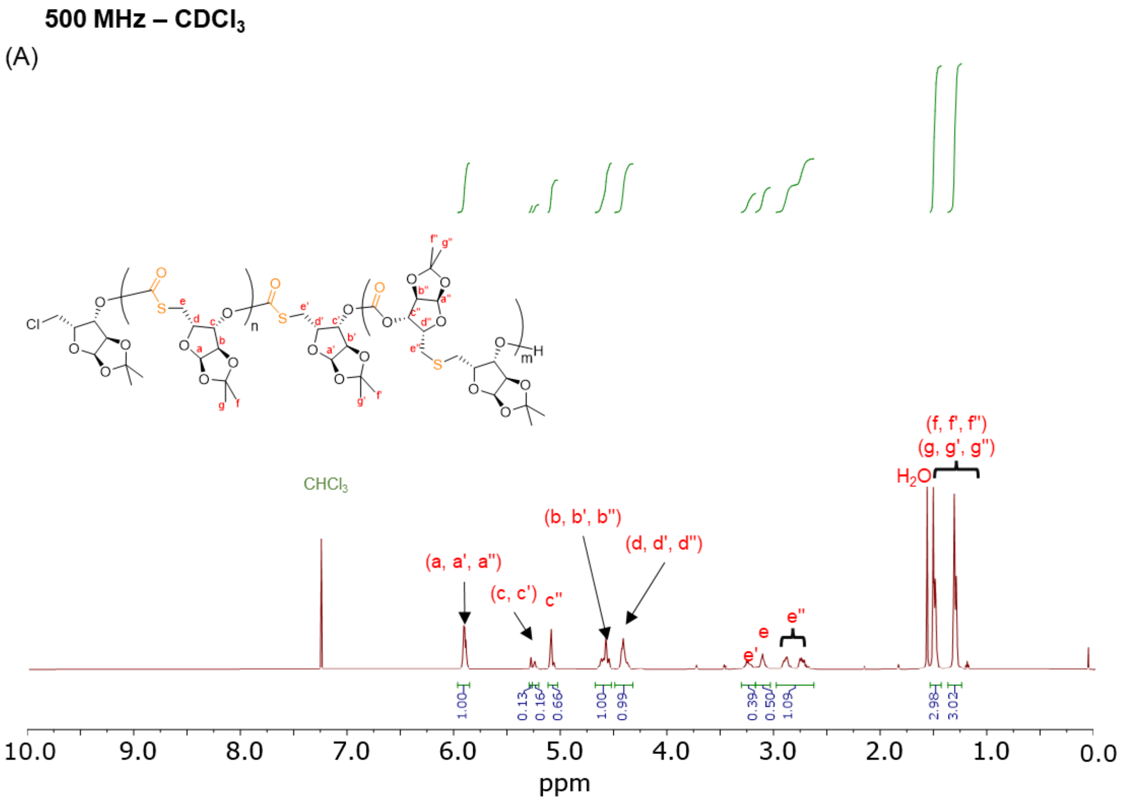

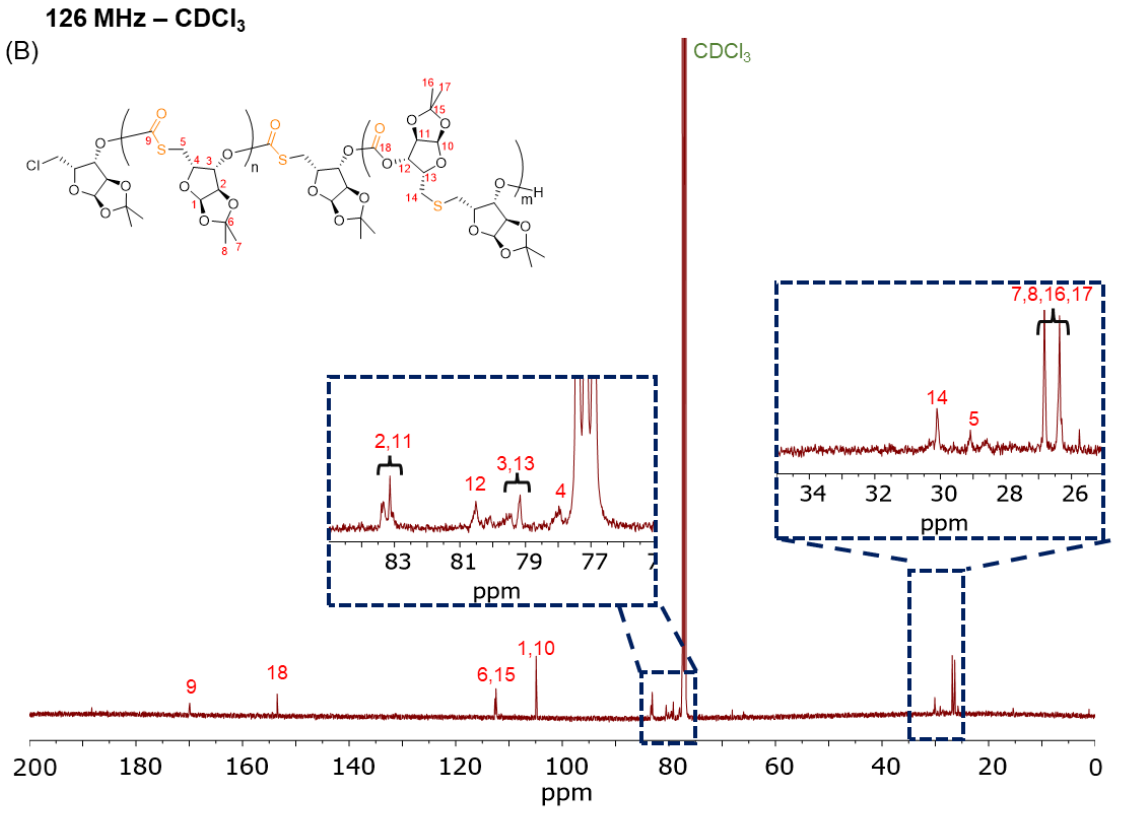

Figure S3: (A) ^{1}H NMR (500 MHz, $CDCl_3$) and (B) ^{13}C NMR (126 MHz, $CDCl_3$) spectrum of polymer ***3***.

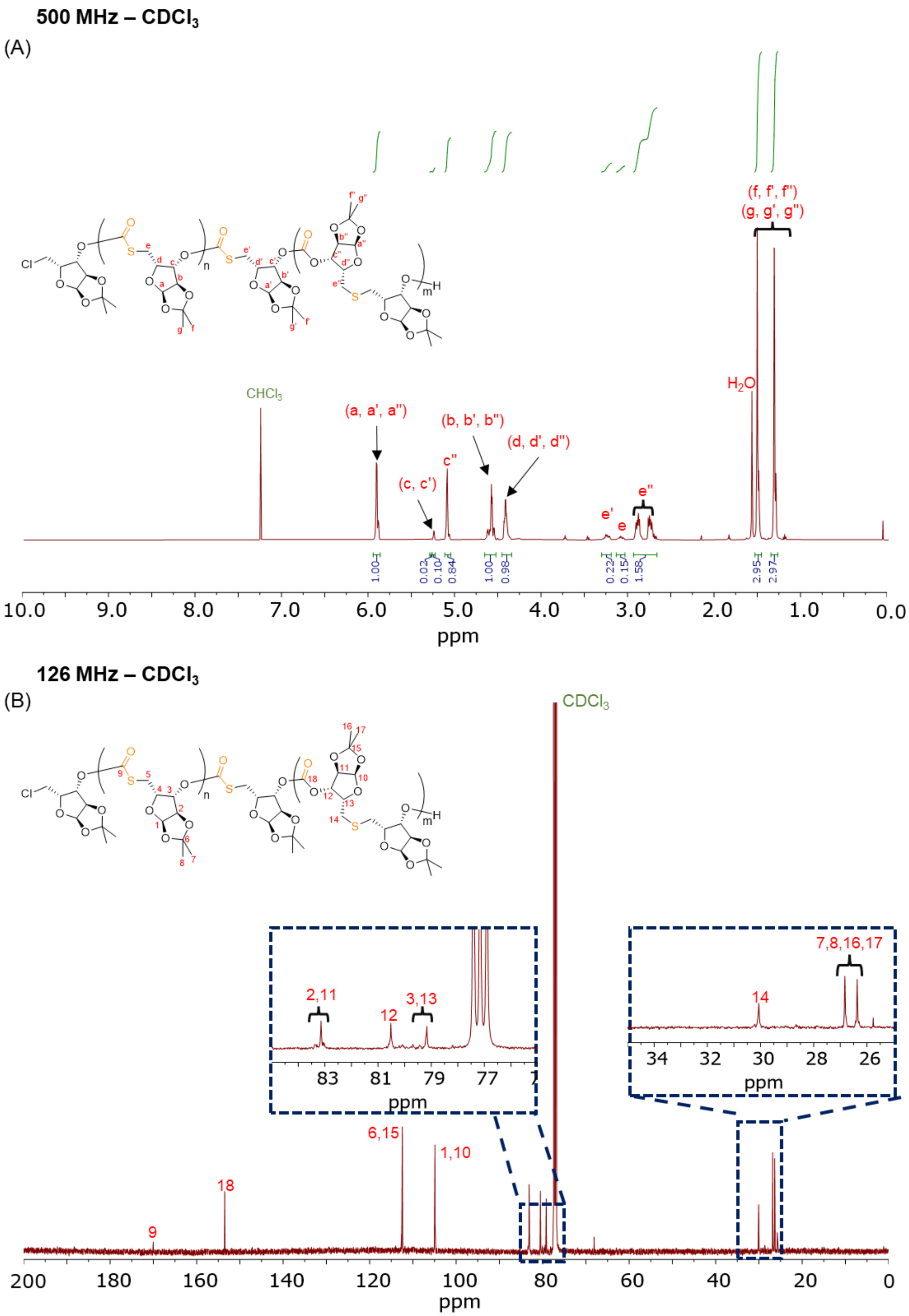

Figure S4: (A) ^{1}H NMR (500 MHz, $CDCl_3$) and (B) ^{13}C NMR (126 MHz, $CDCl_3$) spectrum of polymer ***4***.

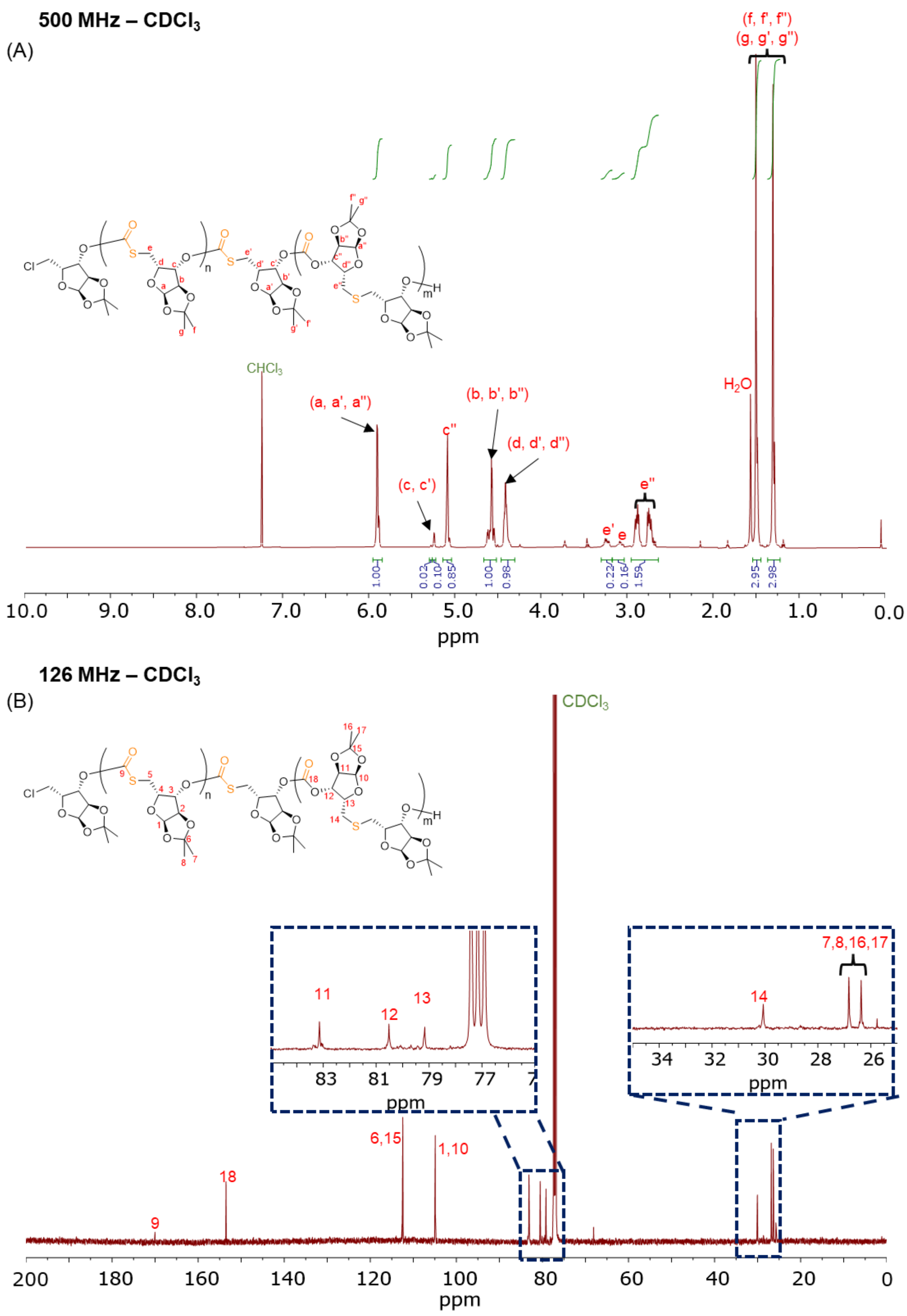

Figure S5: (A) ^{1}H NMR (500 MHz, $CDCl_3$) and (B) ^{13}C NMR (126 MHz, $CDCl_3$) spectrum of polymer ***5***.

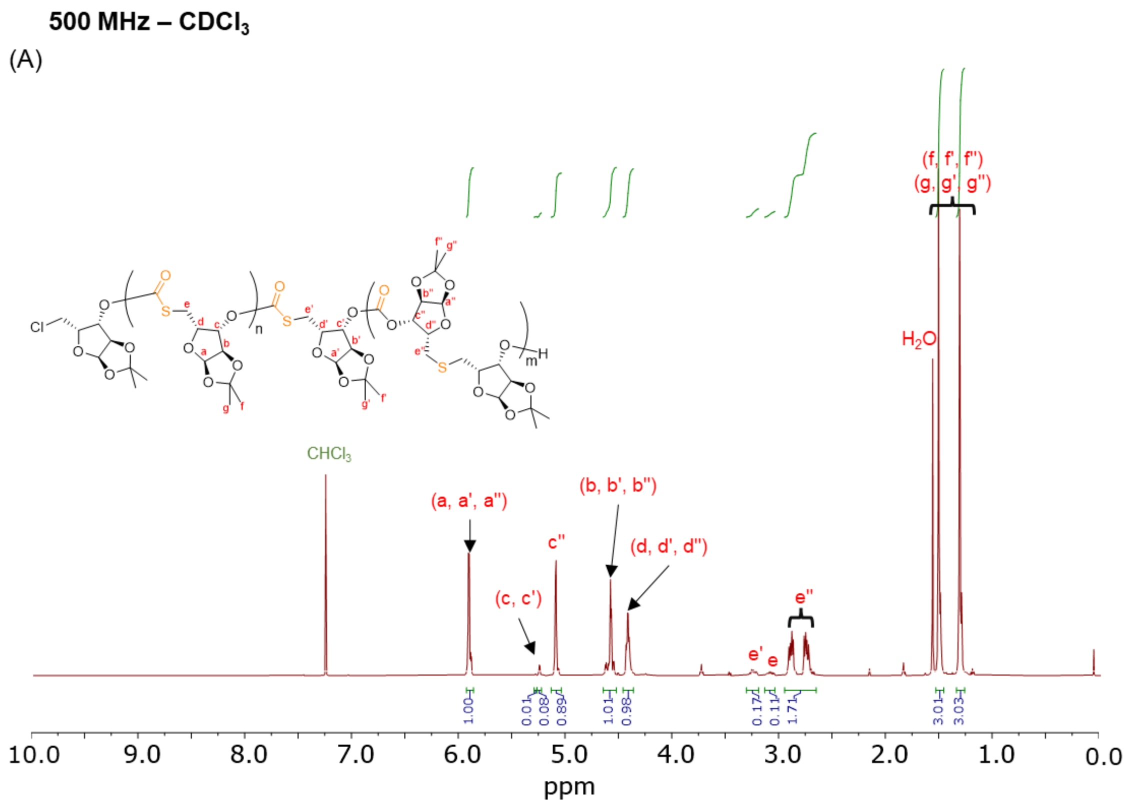

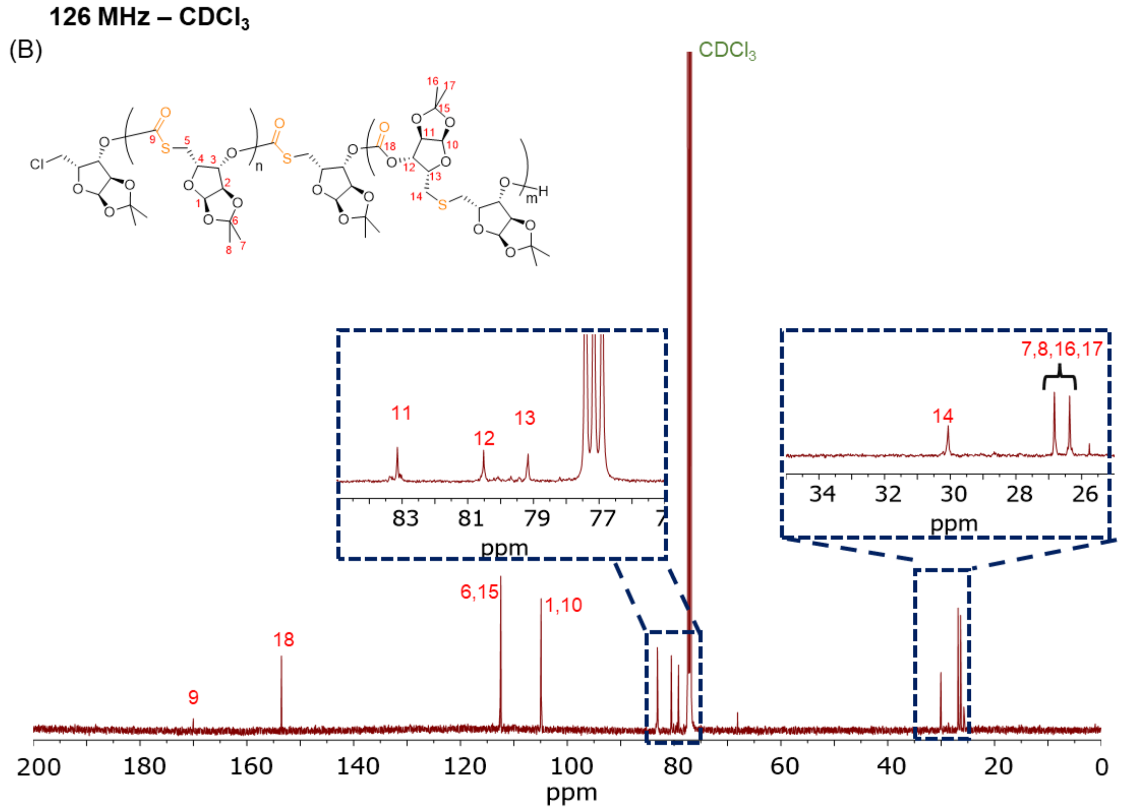

Figure S6: (A) ^{1}H NMR (500 MHz, $CDCl_3$) and (B) ^{13}C NMR (126 MHz, $CDCl_3$) spectrum of polymer ***6***.

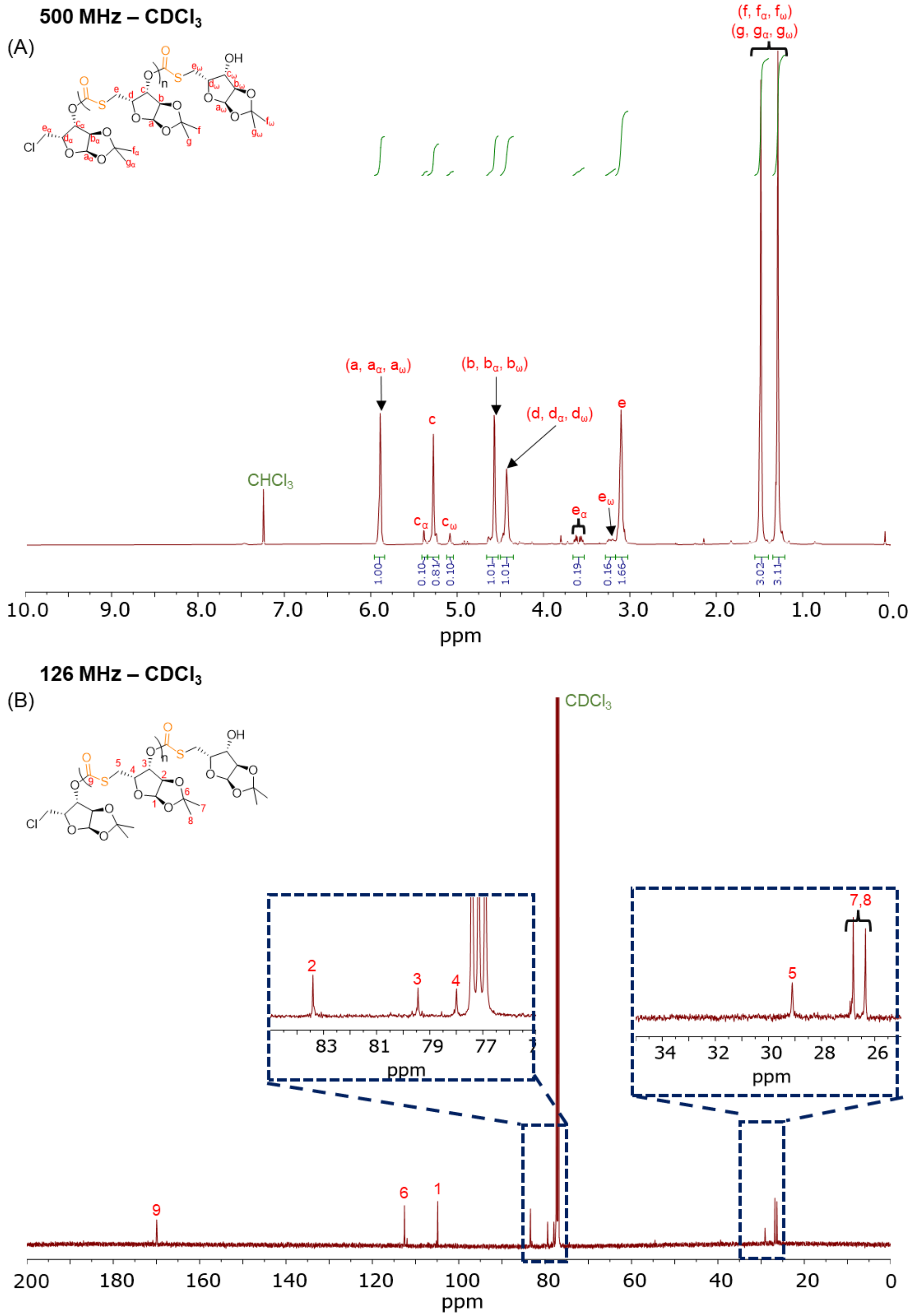

Figure S7: (A) ^{1}H NMR (500 MHz, $CDCl_3$) and (B) ^{13}C NMR (126 MHz, $CDCl_3$) spectrum of polymer ***7***.

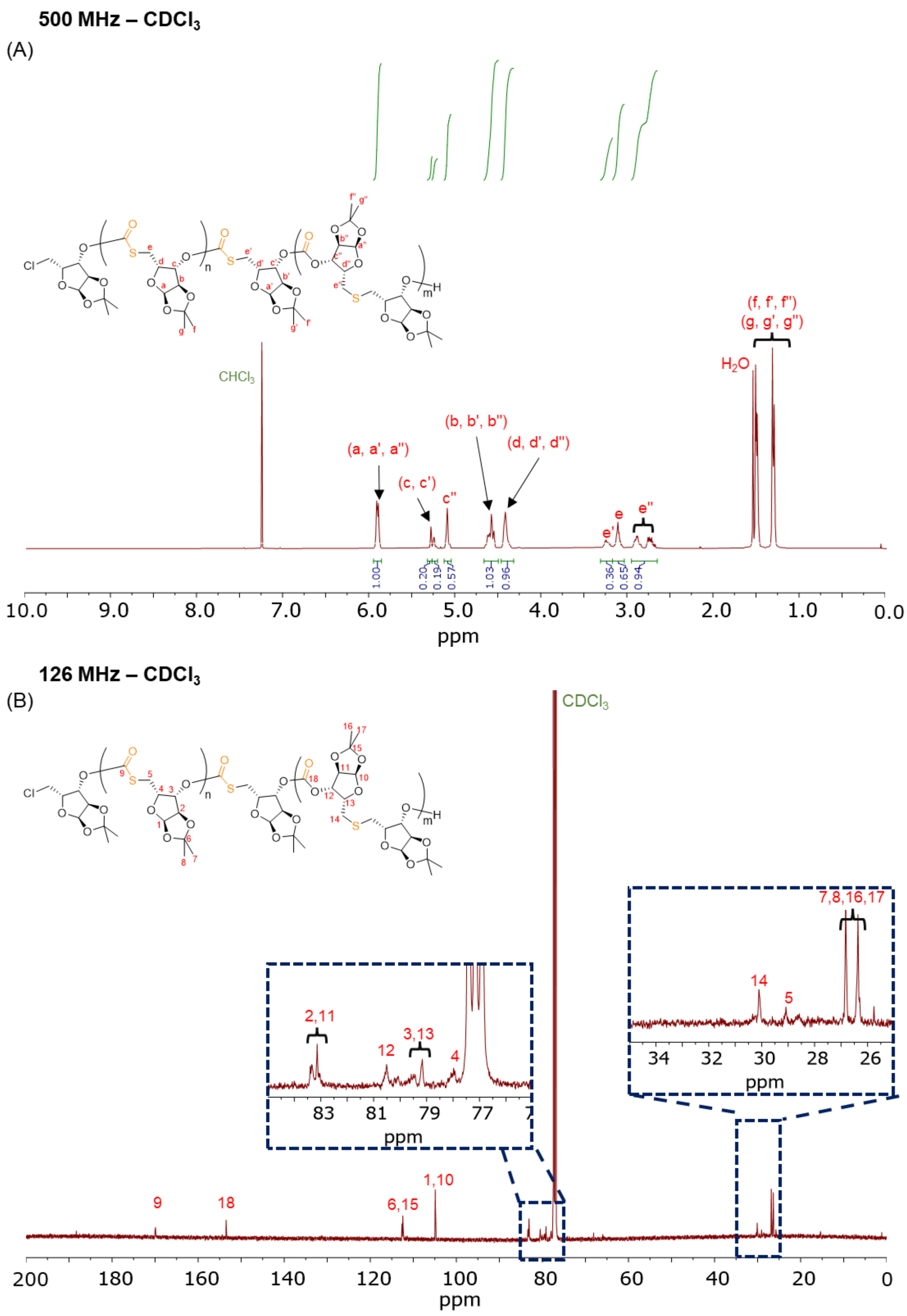

Figure S8: (A) ^{1}H NMR (500 MHz, $CDCl_3$) and (B) ^{13}C NMR (126 MHz, $CDCl_3$) spectrum of polymer ***8***.

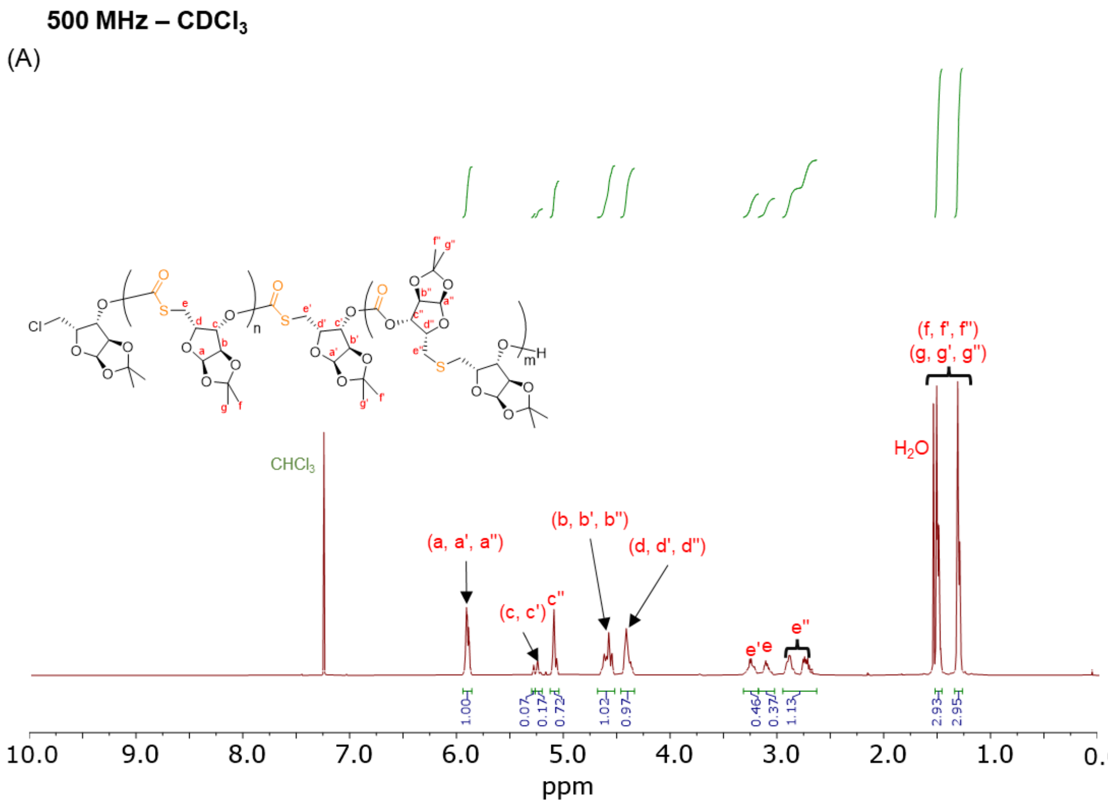

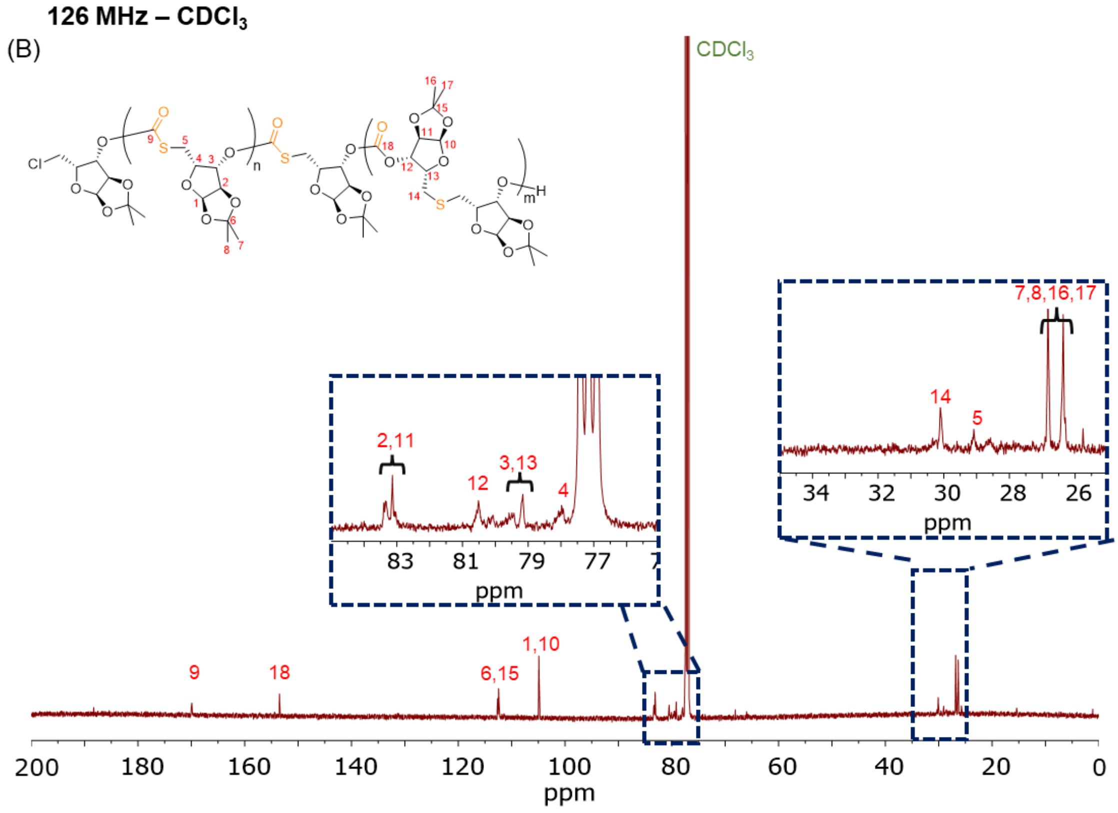

Figure S9: (A) ^{1}H NMR (500 MHz, $CDCl_3$) and (B) ^{13}C NMR (126 MHz, $CDCl_3$) spectrum of polymer ***9***.

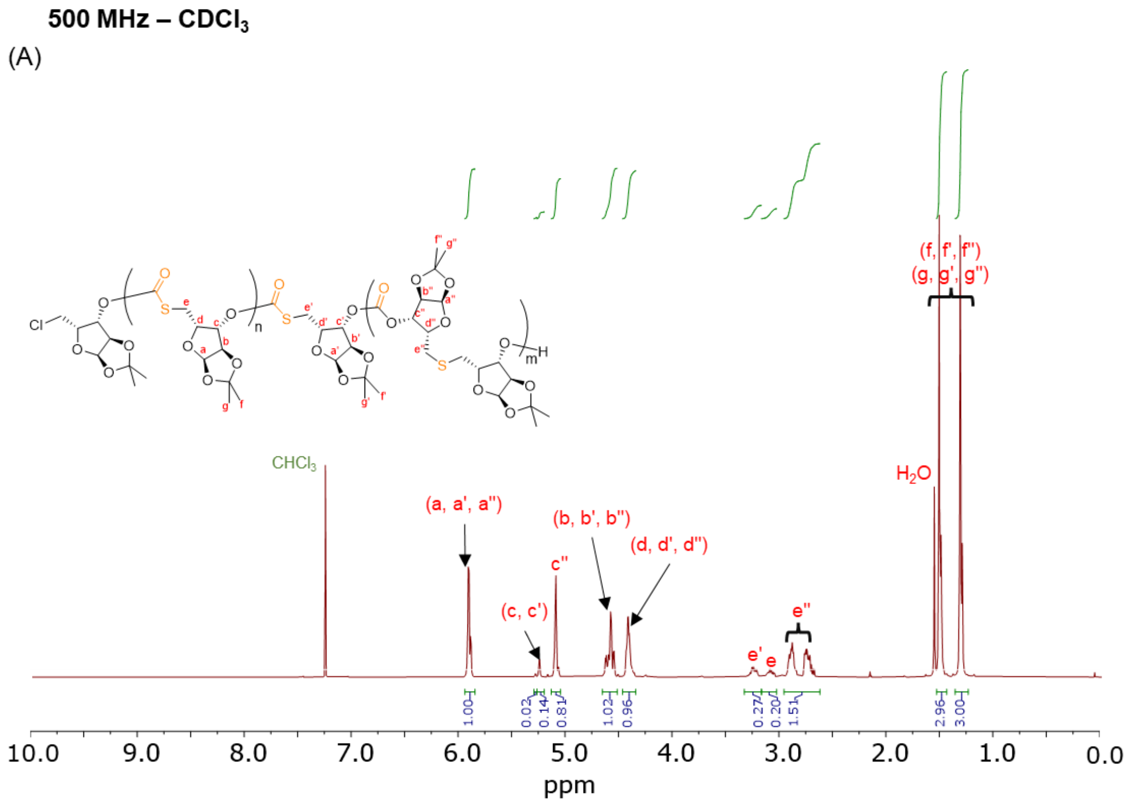

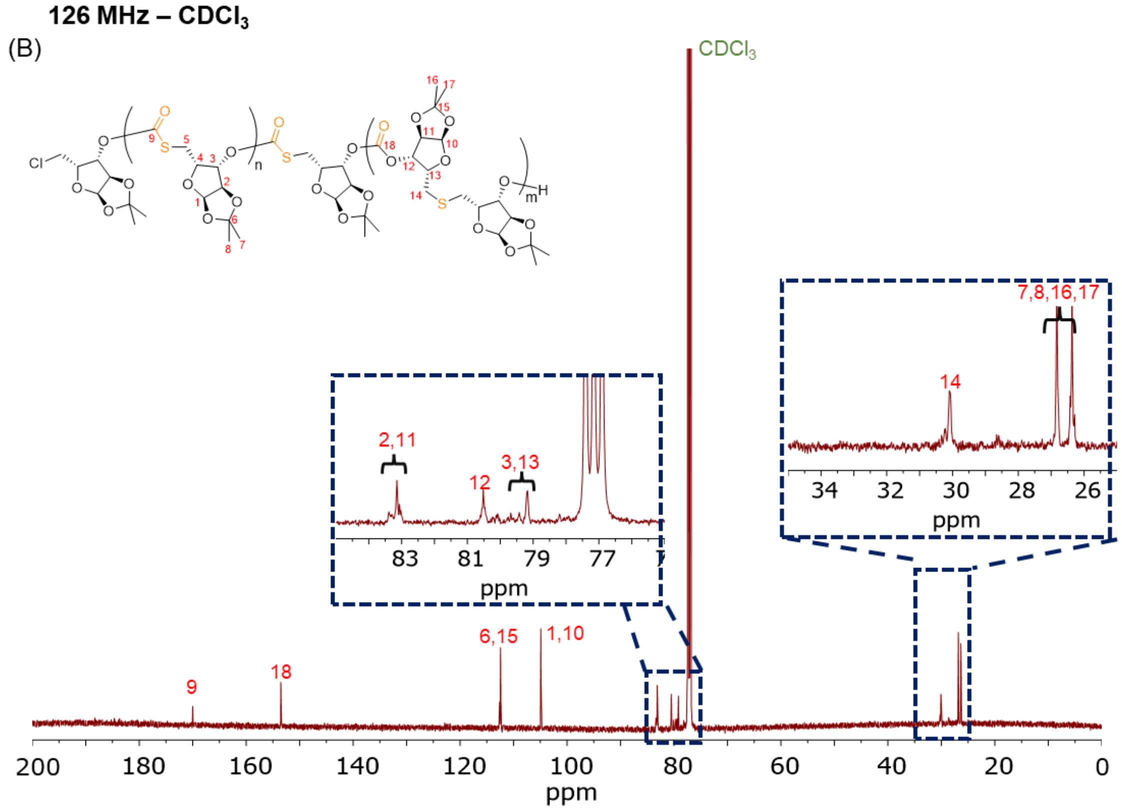

Figure S10: (A) ^{1}H NMR (500 MHz, $CDCl_3$) and (B) ^{13}C NMR (126 MHz, $CDCl_3$) spectrum of polymer ***10***.

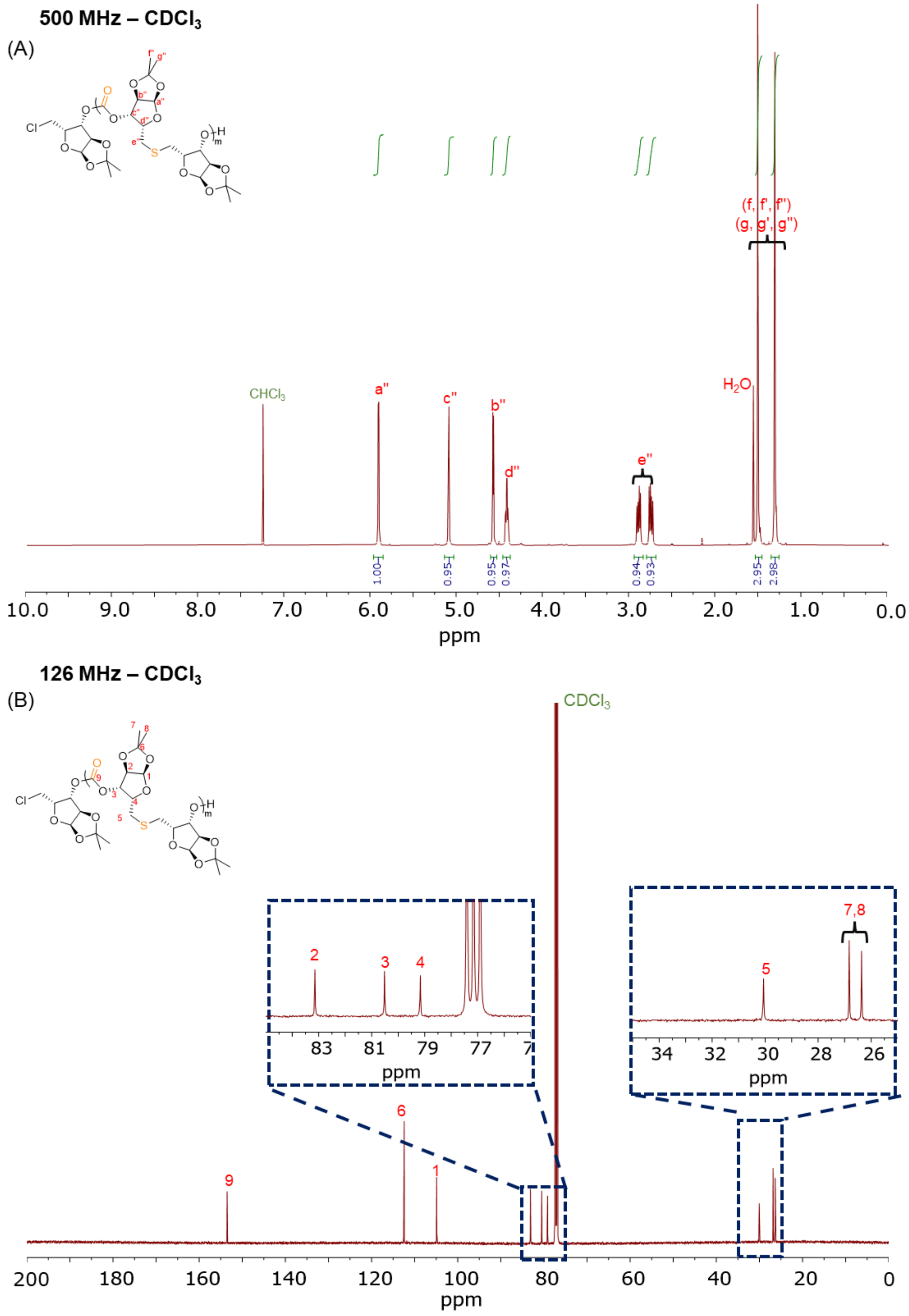

Figure S11: (A) ^{1}H NMR (500 MHz, $CDCl_3$) and (B) ^{13}C NMR (126 MHz, $CDCl_3$) spectrum of polymer ***11***.

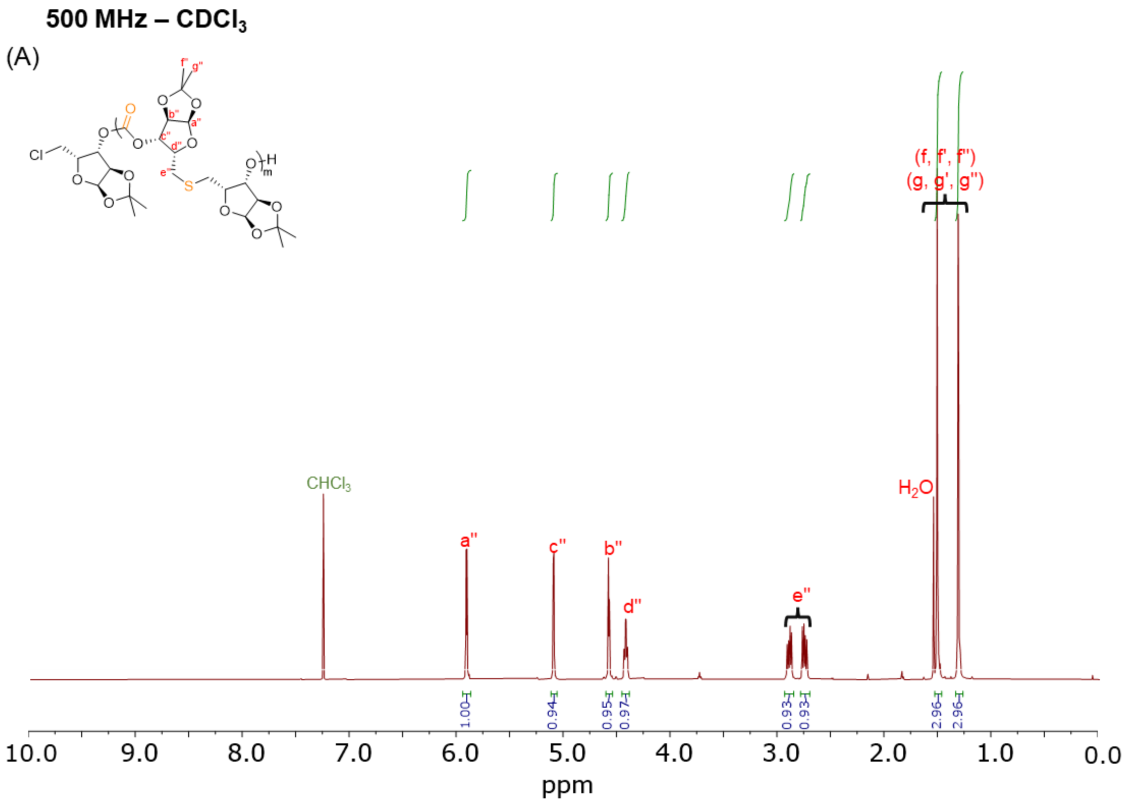

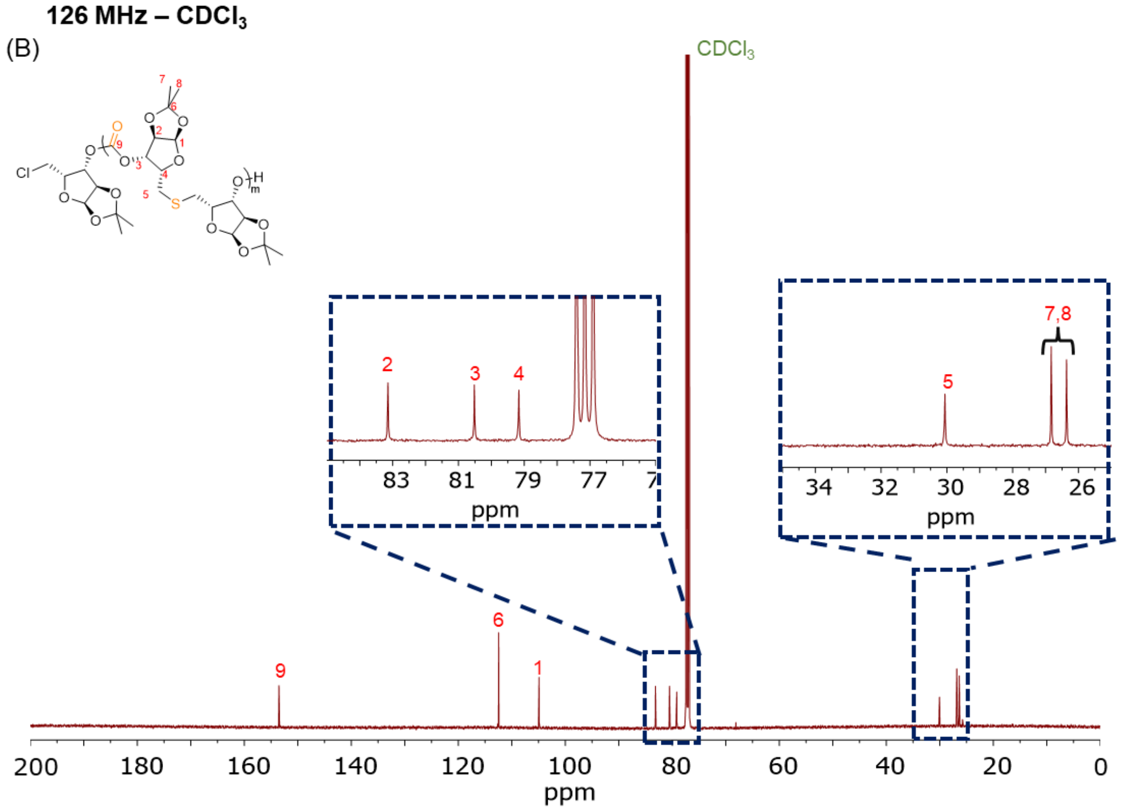

Figure S12: (A) ^{1}H NMR (500 MHz, $CDCl_3$) and (B) ^{13}C NMR (126 MHz, $CDCl_3$) spectrum of polymer ***12***.

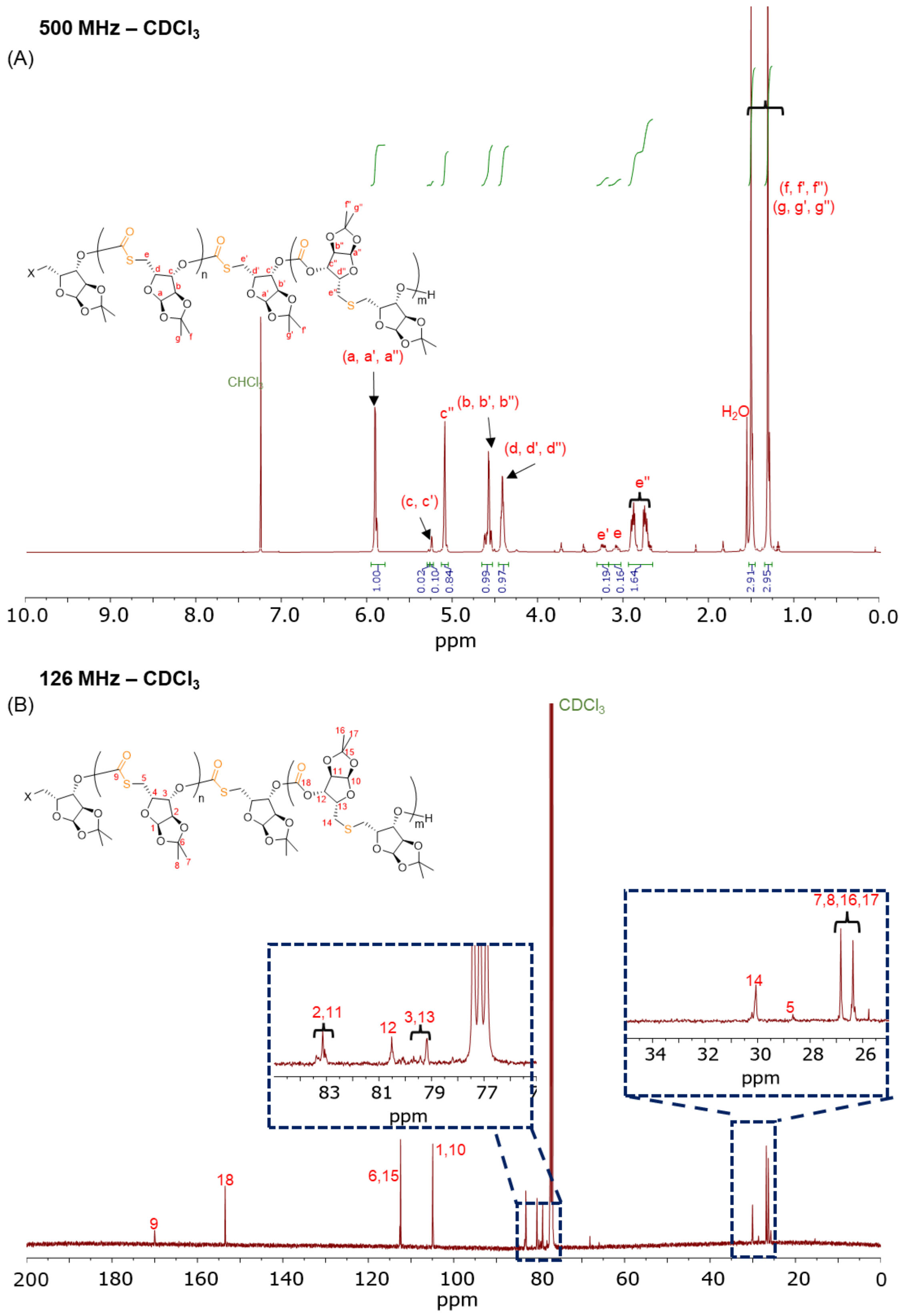

Figure S13: (A) ^{1}H NMR (500 MHz, $CDCl_3$) and (B) ^{13}C NMR (126 MHz, $CDCl_3$) spectrum of polymer ***13***.

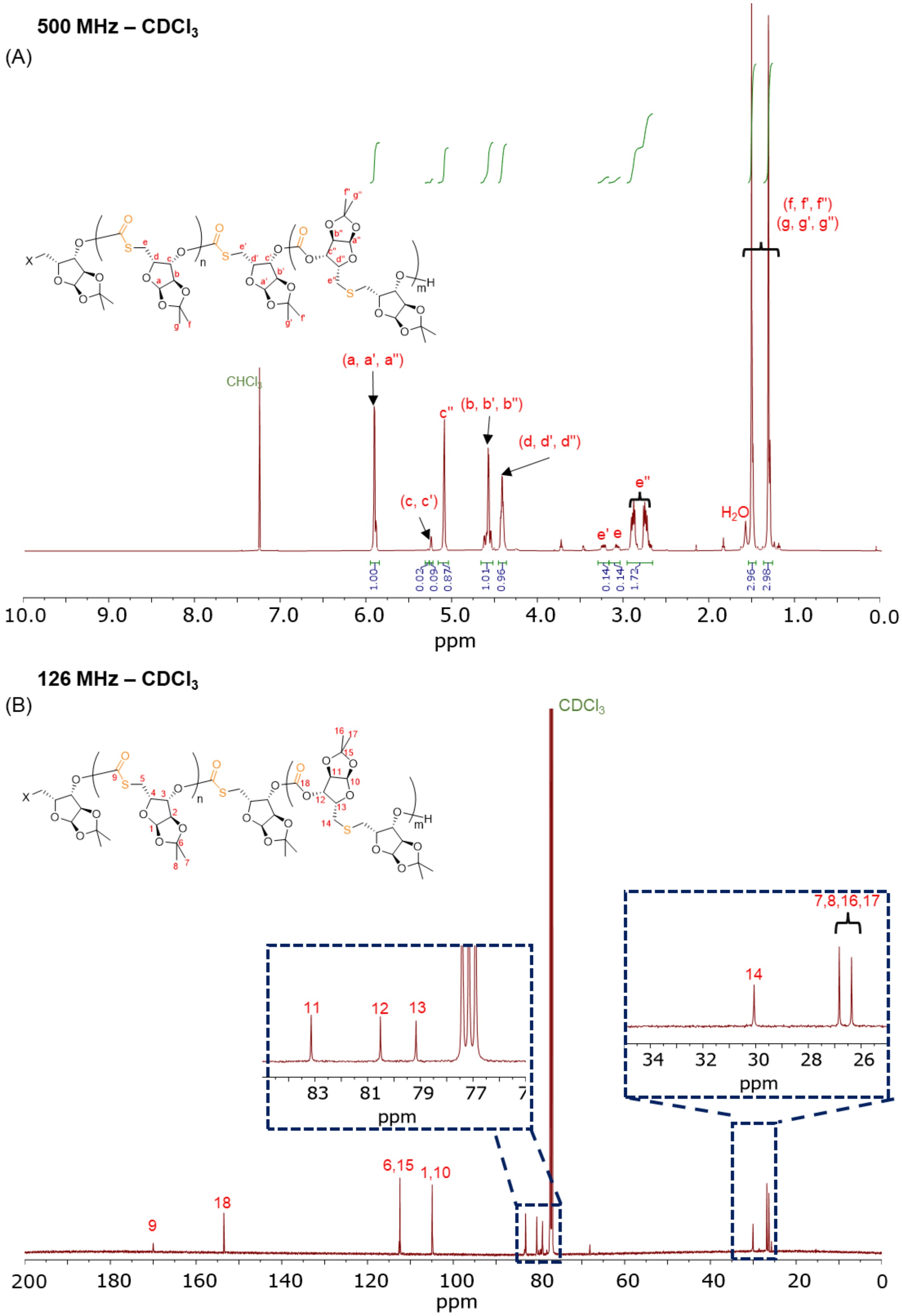

Figure S14: (A) ^{1}H NMR (500 MHz, $CDCl_3$) and (B) ^{13}C NMR (126 MHz, $CDCl_3$) spectrum of polymer ***14***.

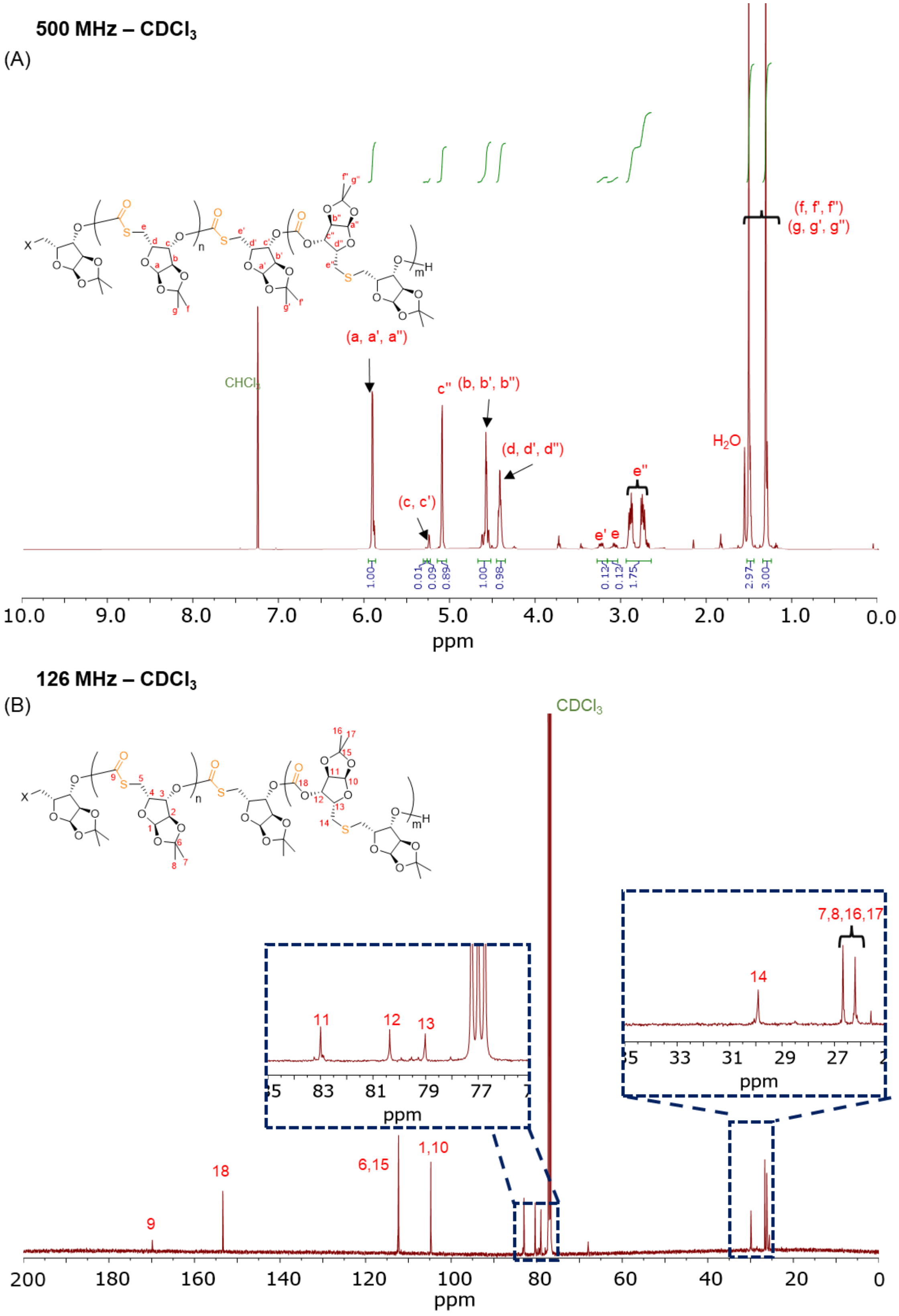

Figure S15: (A) ^{1}H NMR (500 MHz, $CDCl_3$) and (B) ^{13}C NMR (126 MHz, $CDCl_3$) spectrum of polymer ***15***.

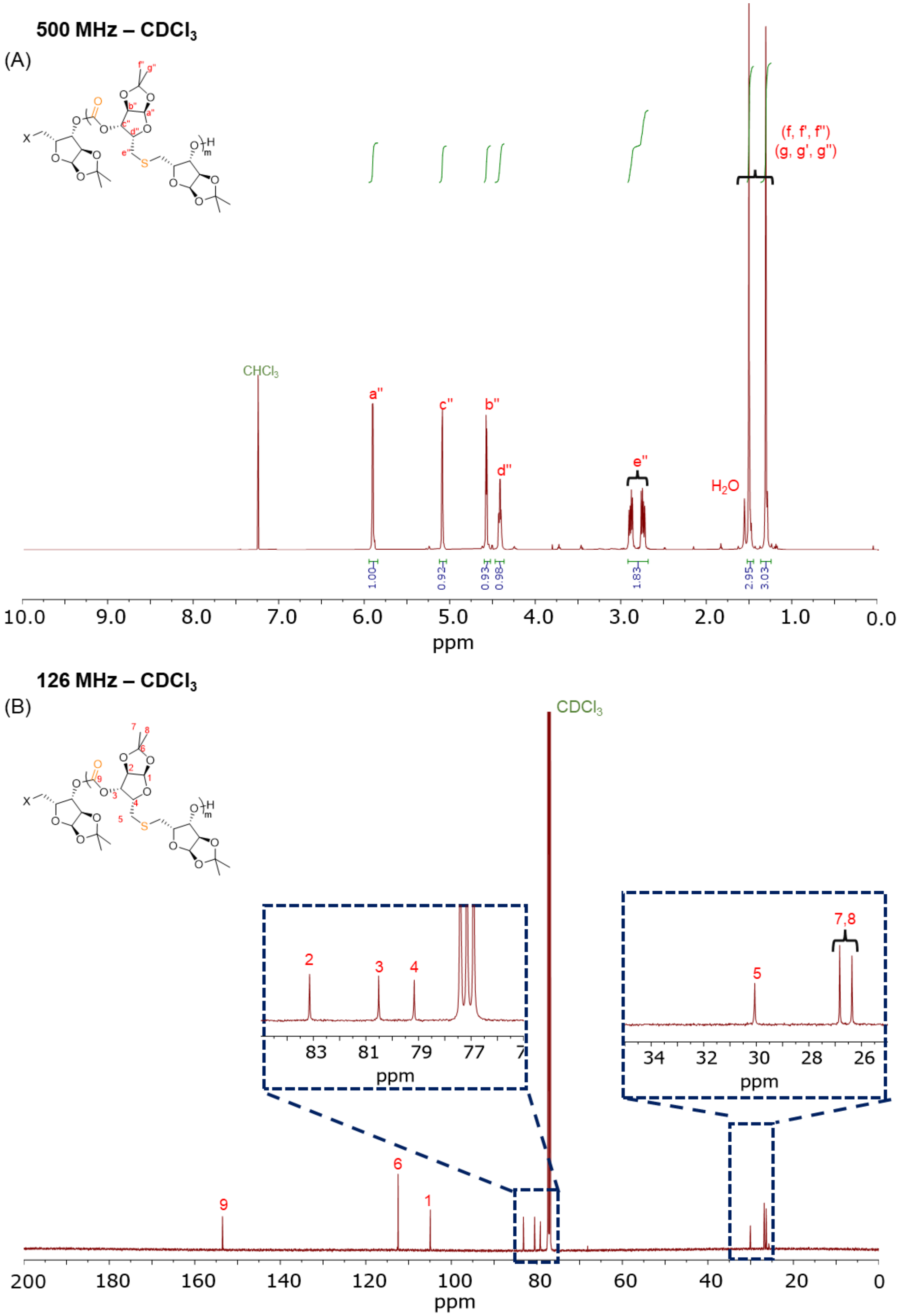

Figure S16: (A) ^{1}H NMR (500 MHz, $CDCl_3$) and (B) ^{13}C NMR (126 MHz, $CDCl_3$) spectrum of polymer ***16***.

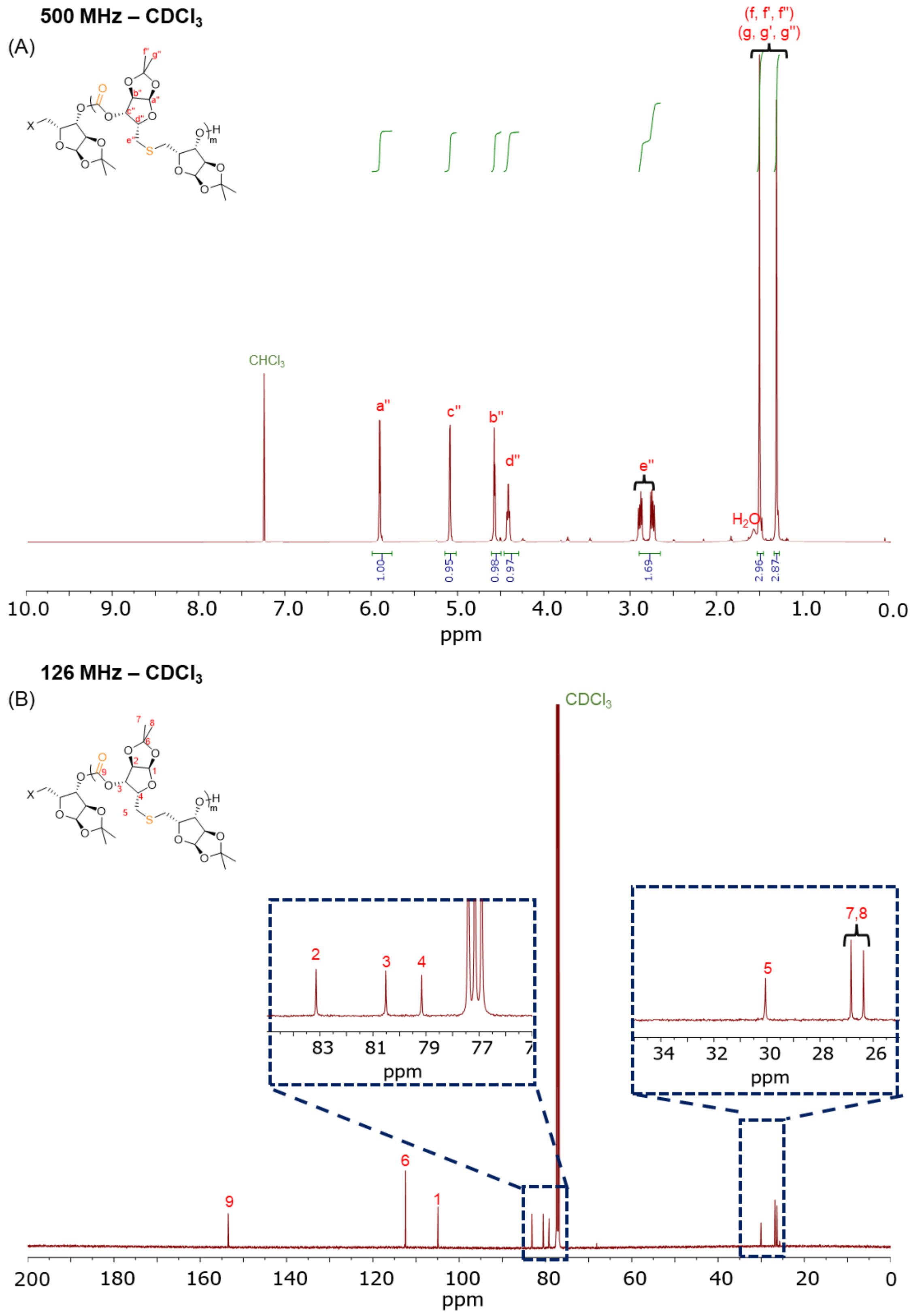

Figure S17: (A) ^{1}H NMR (500 MHz, $CDCl_3$) and (B) ^{13}C NMR (126 MHz, $CDCl_3$) spectrum of polymer ***17***.

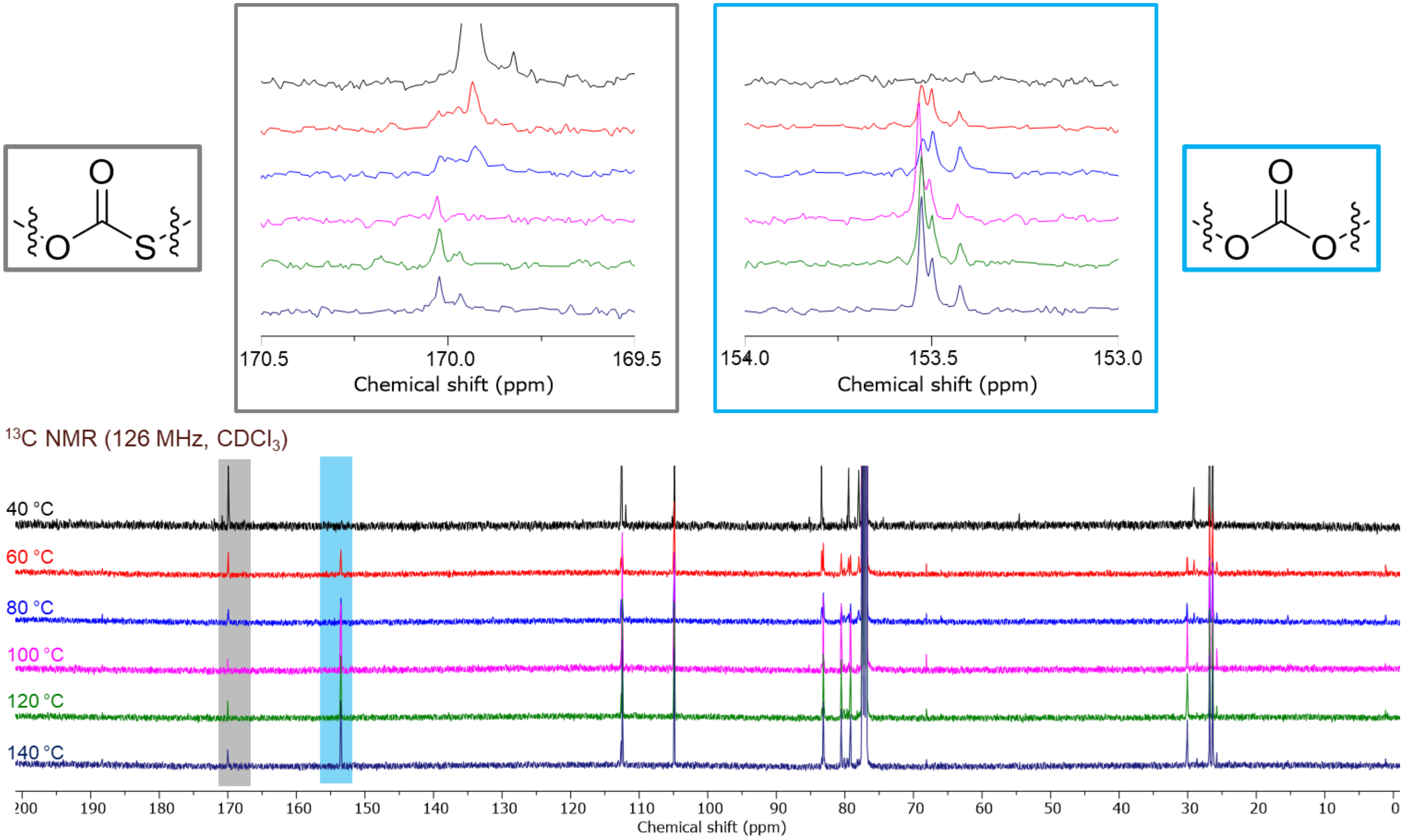

Figure S18: Stacked ^{13}C spectra of polymers ***1*** (black trace), ***2*** (red trace), ***3*** (blue trace), ***4*** (purple trace), ***5*** (green trace), and ***6*** (dark blue trace) with expanded regions at 170 ppm and 150 ppm corresponding to the monothiocarbonate and carbonate linkages.

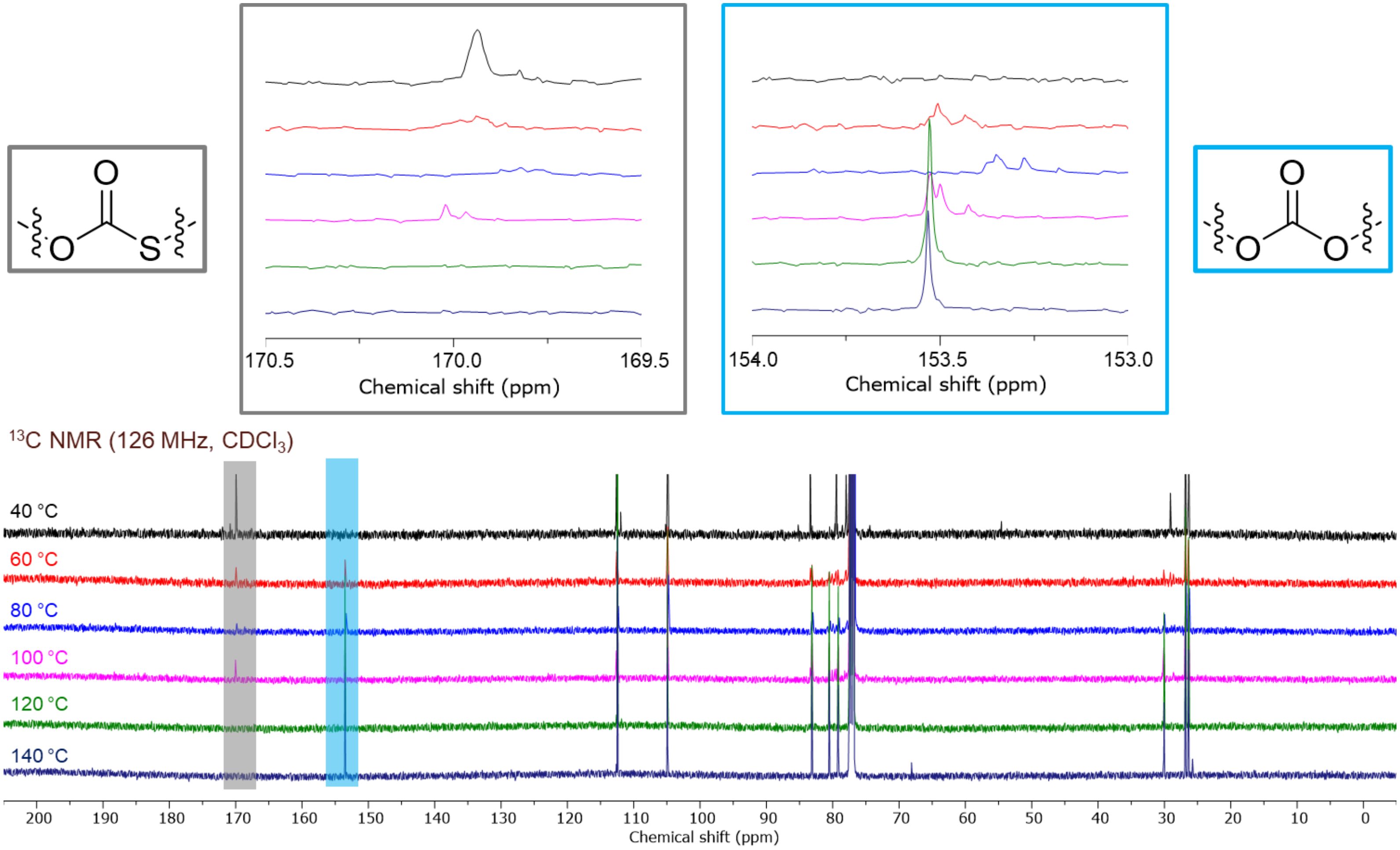

Figure S19: Stacked ^{13}C spectra of polymers ***7*** (black trace), ***8*** (red trace), ***9*** (blue trace), ***10*** (purple trace), ***11*** (green trace), and ***12*** (dark blue trace) with expanded regions at 170 ppm and 150 ppm corresponding to the monothiocarbonate and carbonate linkages.

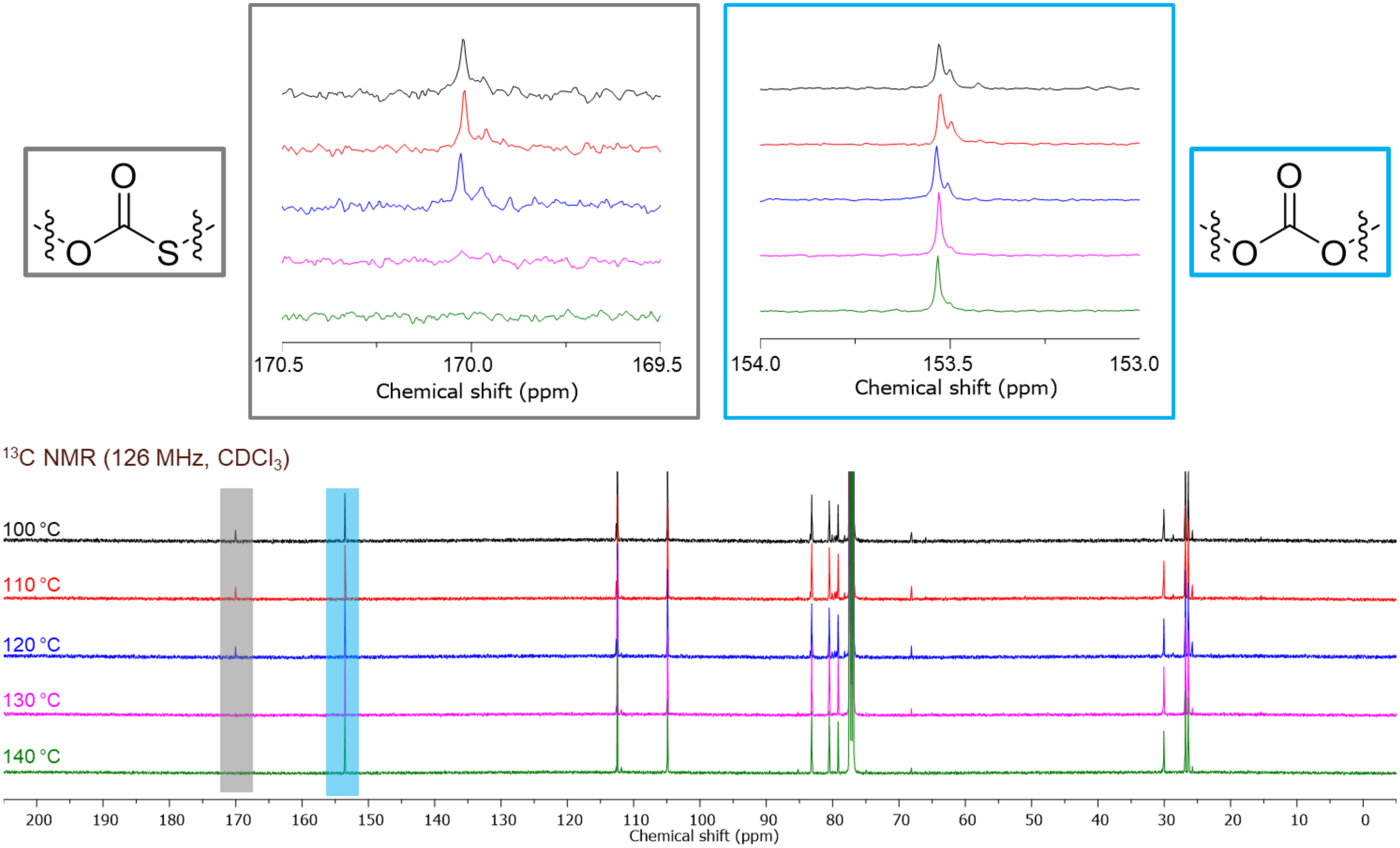

Figure S20: Stacked ^{13}C spectra of polymers ***13*** (black trace), ***14*** (red trace), ***15*** (blue trace), ***16*** (purple trace), and ***17*** (green trace) with expanded regions at 170 ppm and 150 ppm corresponding to the monothiocarbonate and carbonate linkages.

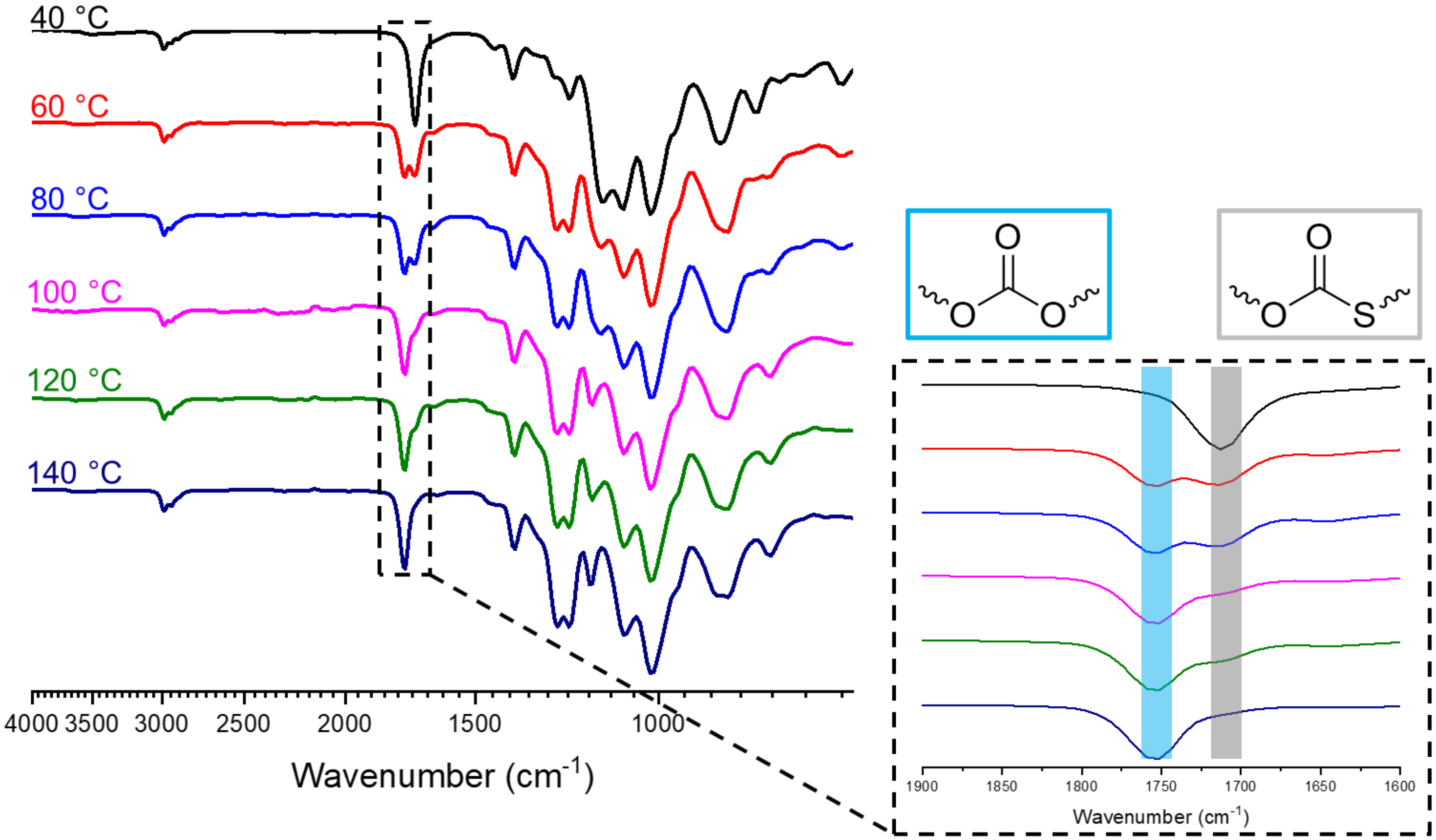

Figure S21: FT-IR spectra of polymers ***1*** (black trace), ***2*** (red trace), ***3*** (blue trace), ***4*** (purple trace), ***5*** (green trace), and ***6*** (dark blue trace).

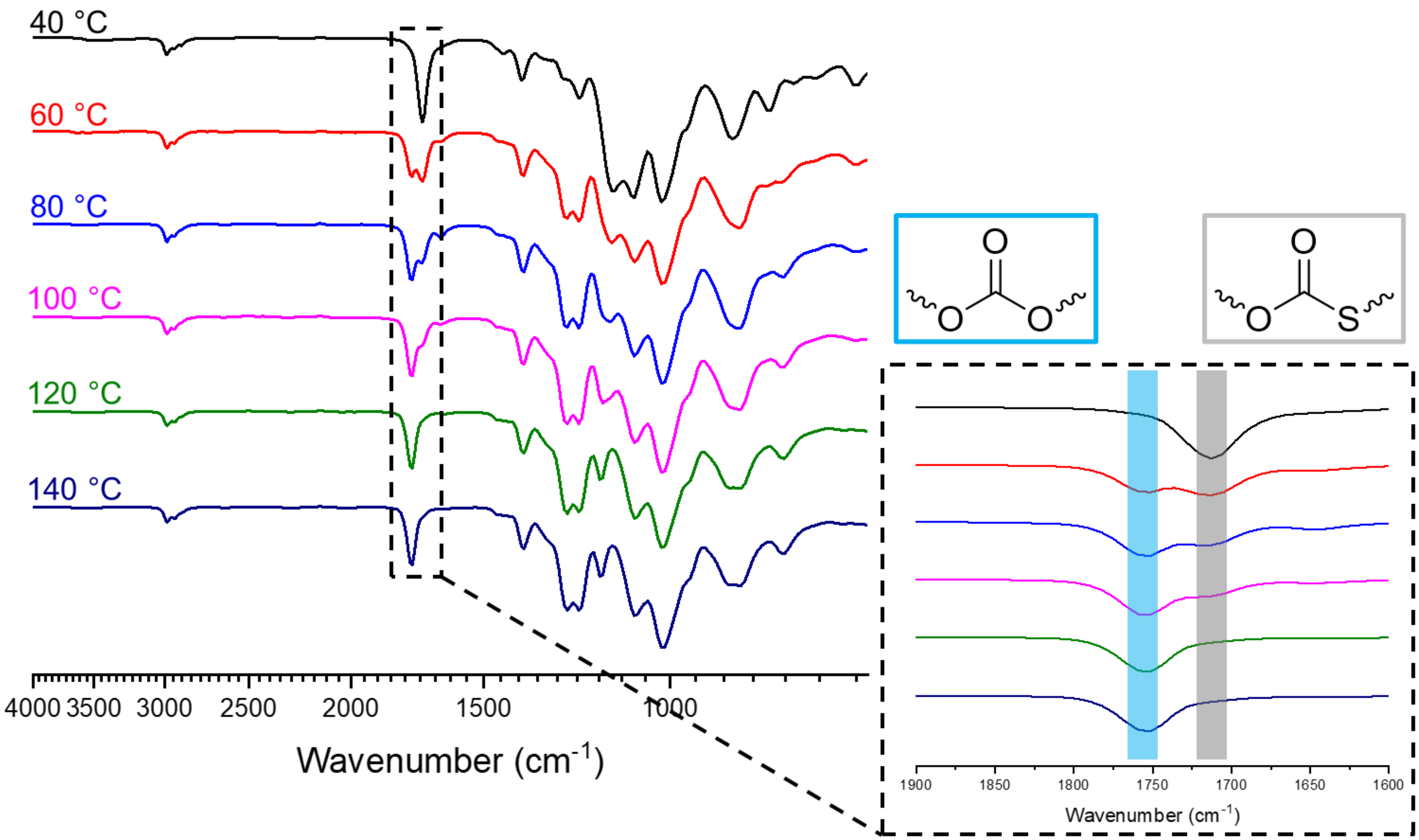

Figure S22: FT-IR spectra of polymers ***7*** (black trace), ***8*** (red trace), ***9*** (blue trace), ***10*** (purple trace), ***11*** (green trace), and ***12*** (dark blue trace).

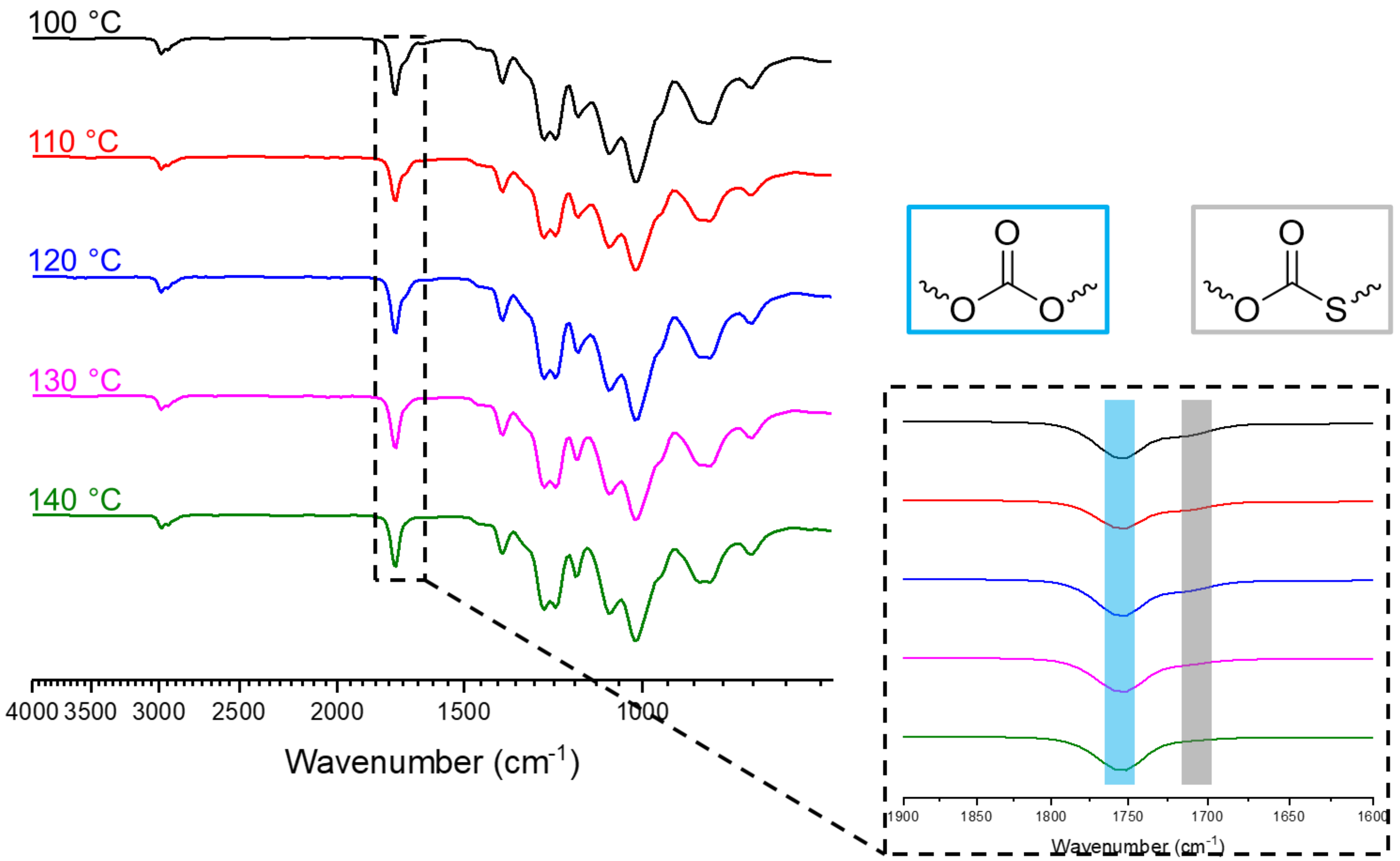

Figure S23: FT-IR spectra of polymers ***13*** (black trace), ***14*** (red trace), ***15*** (blue trace), ***16*** (purple trace), and ***17*** (green trace).

.

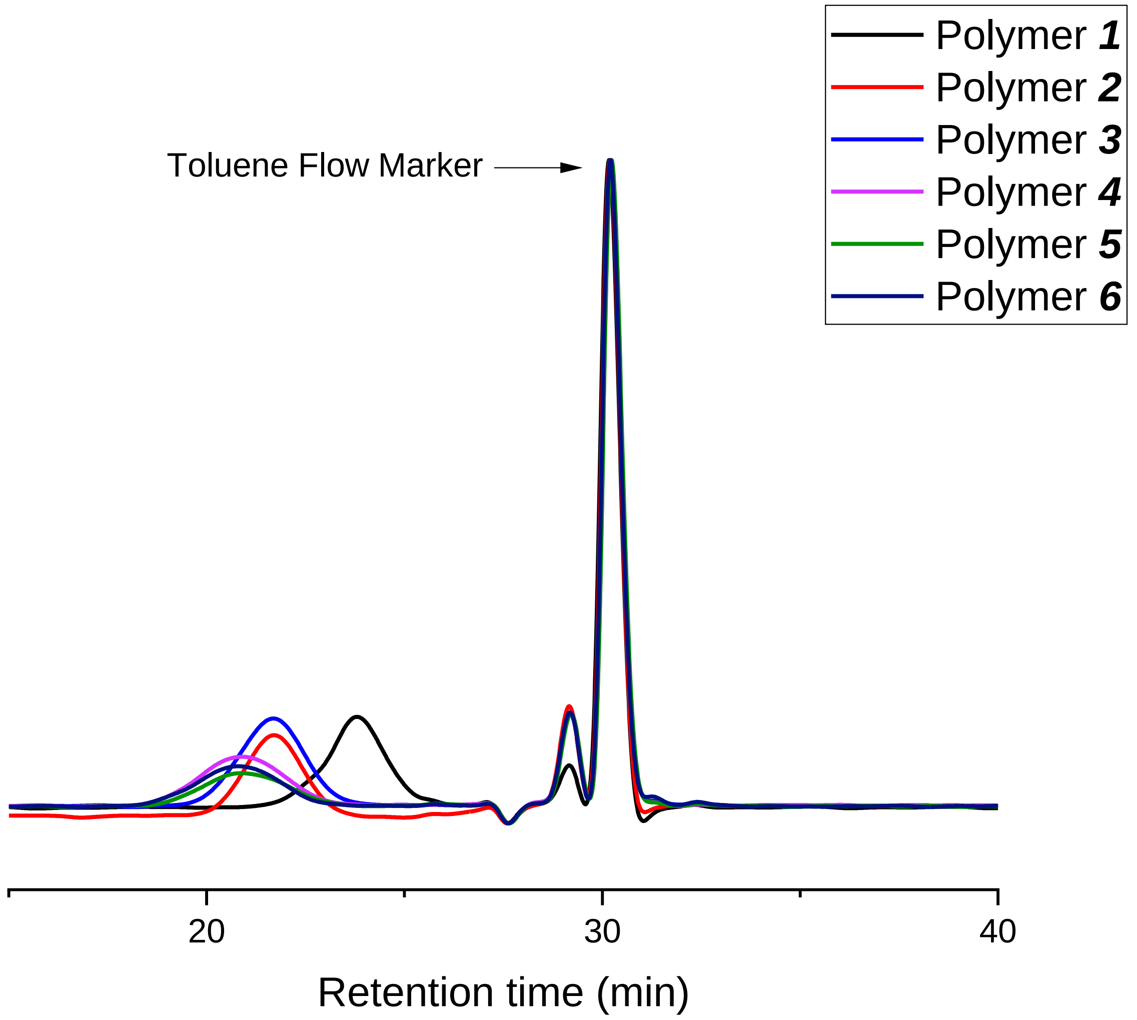

Figure S24: SEC traces of polymers *1* – *6*.

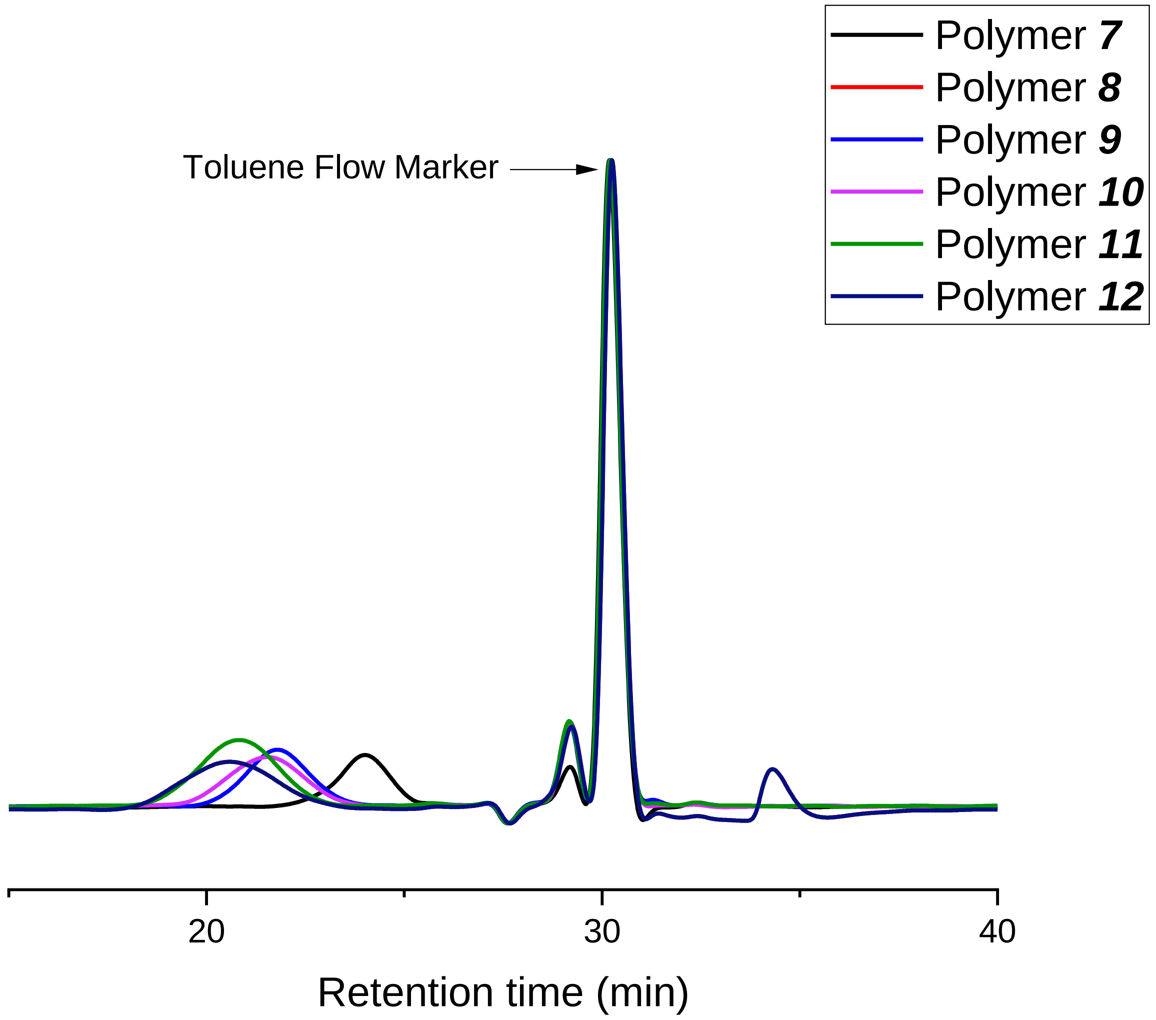

Figure S25: SEC traces of polymers *7* – *12*.

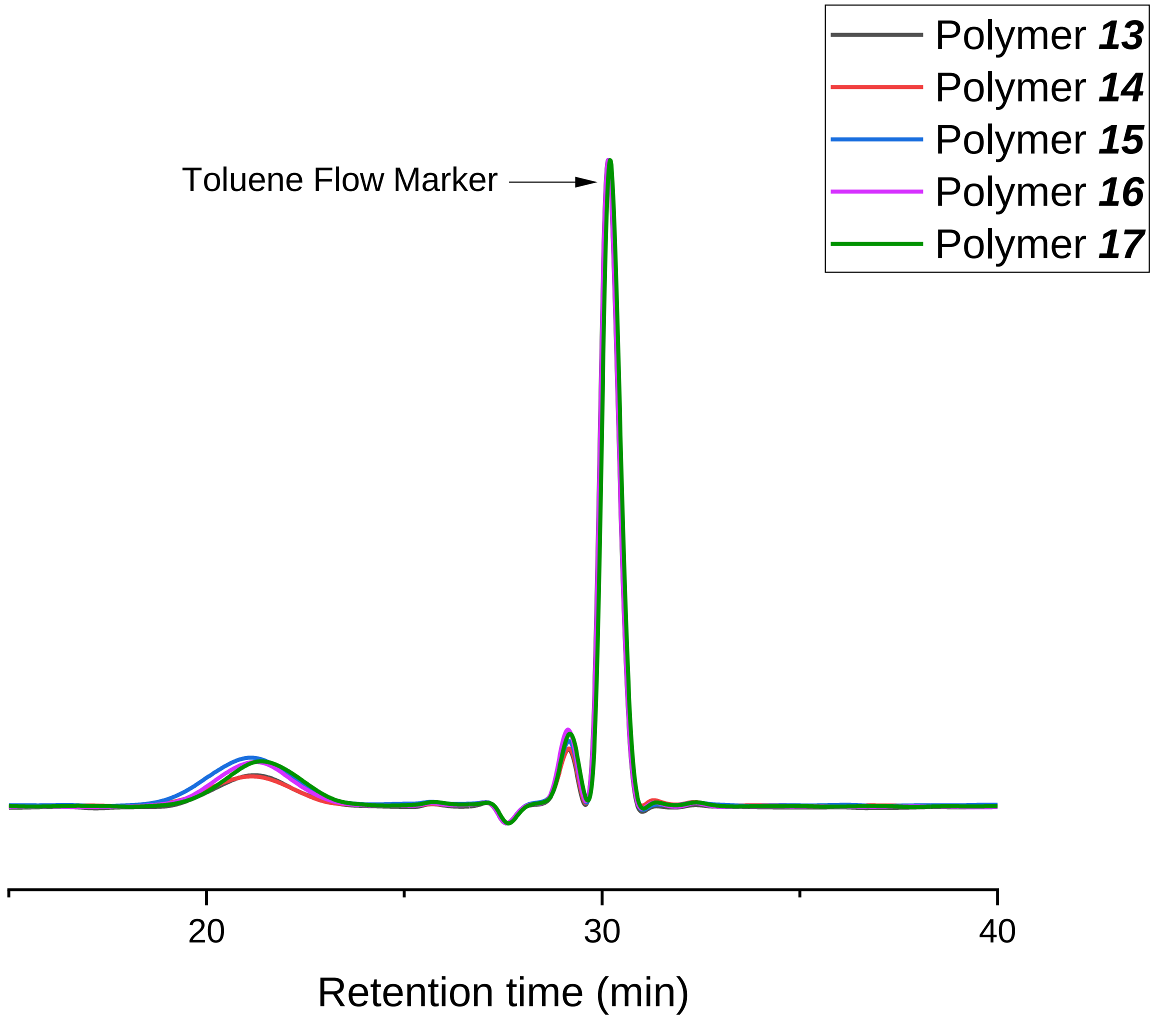

Figure S26: SEC traces of polymers *13 – 17*.

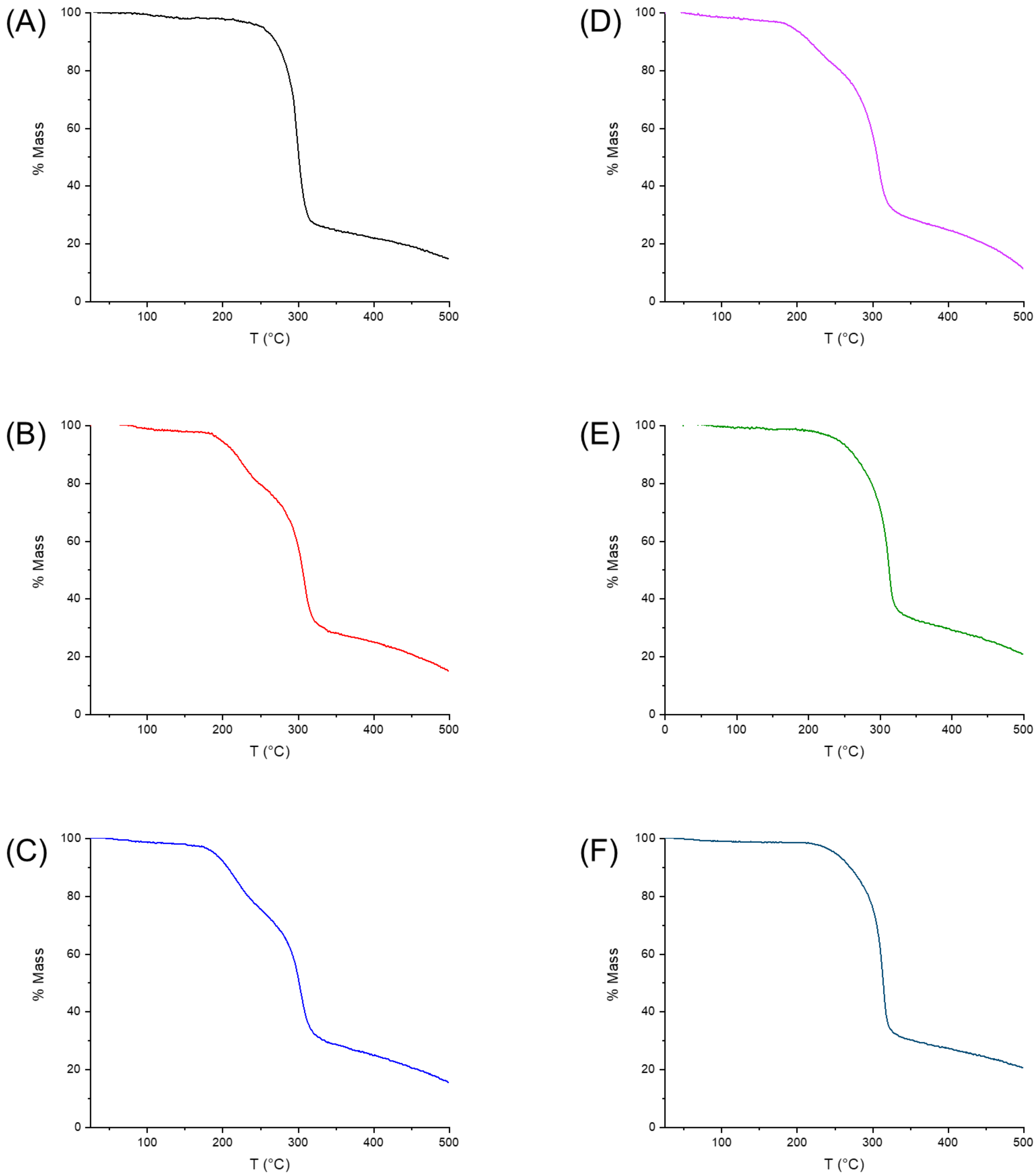

Figure S27: TGA traces of polymers (A) ***1*** (black trace), (B) ***2*** (red trace), (C) ***3*** (blue trace), (D) ***4*** (purple trace), (E) ***5*** (green trace), and (F) ***6*** (dark blue trace).

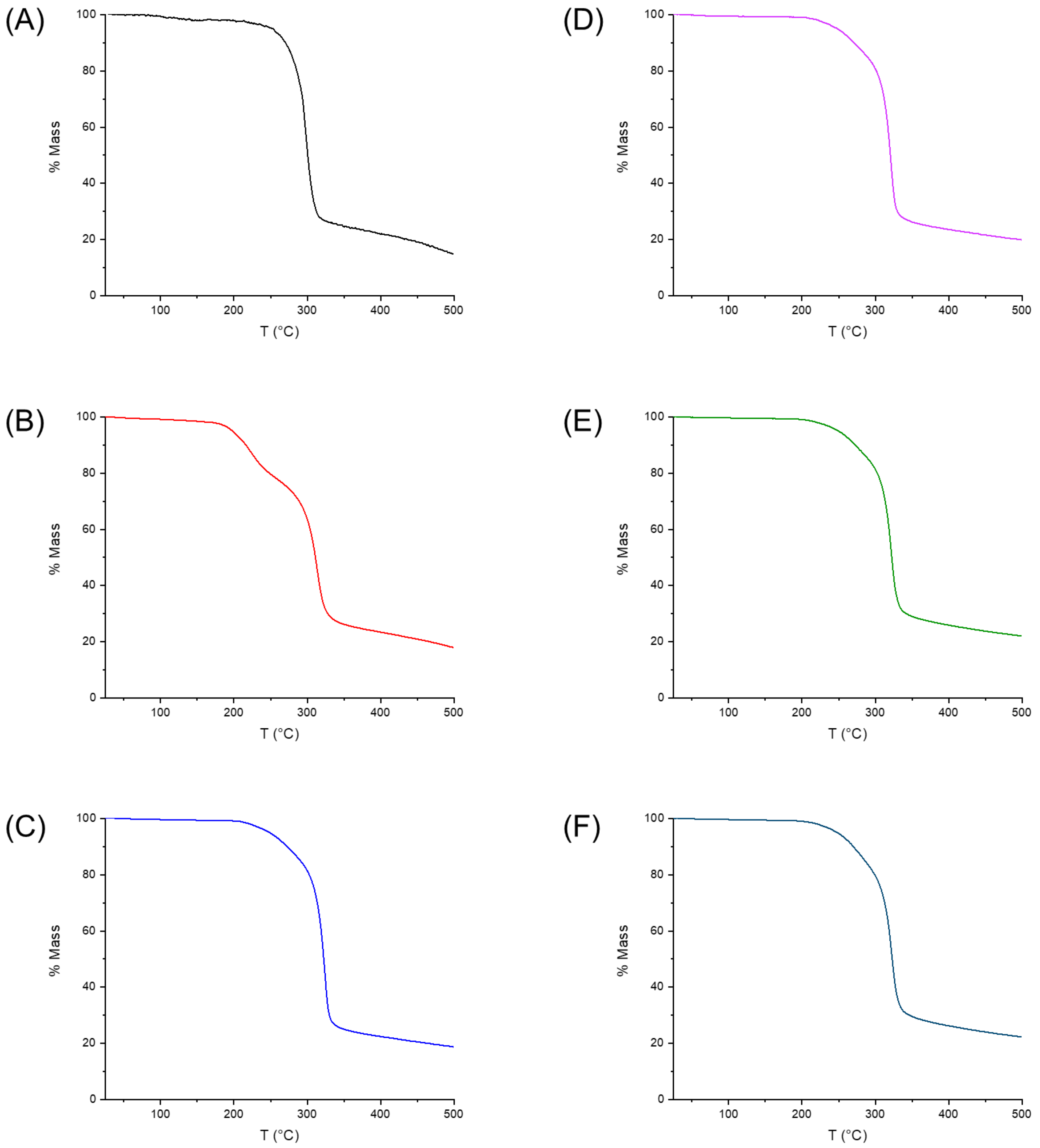

Figure S28: TGA traces of polymers (A) ***7*** (black trace), (B) ***8*** (red trace), (C) ***9*** (blue trace), (D) ***10*** (purple trace), (E) ***11*** (green trace), and (F) ***12*** (dark blue trace).

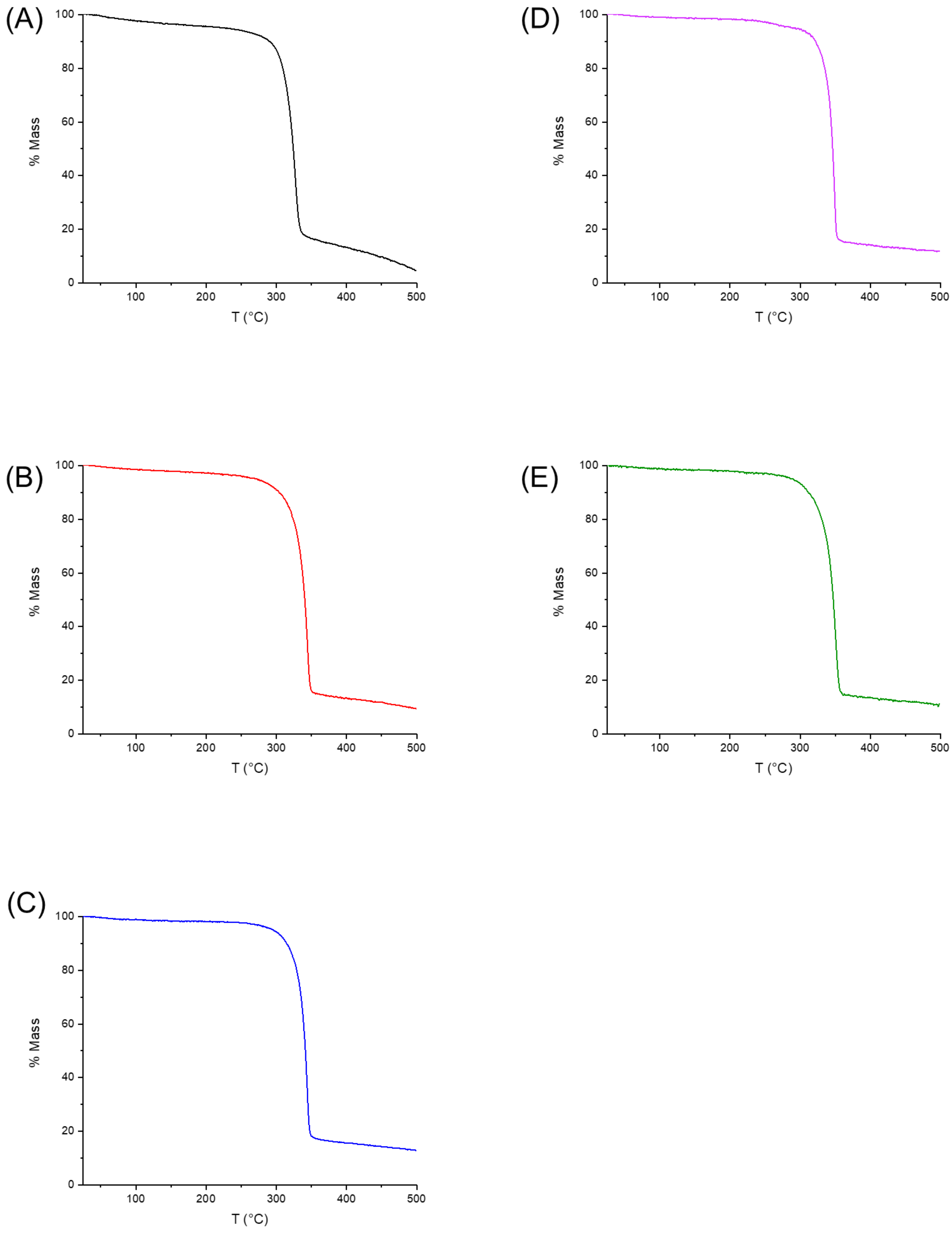

Figure S29: TGA traces of polymers (A) ***13*** (black trace), (B) ***14*** (red trace), (C) ***15*** (blue trace), (D) ***16*** (purple trace), and (E) ***17*** (green trace).

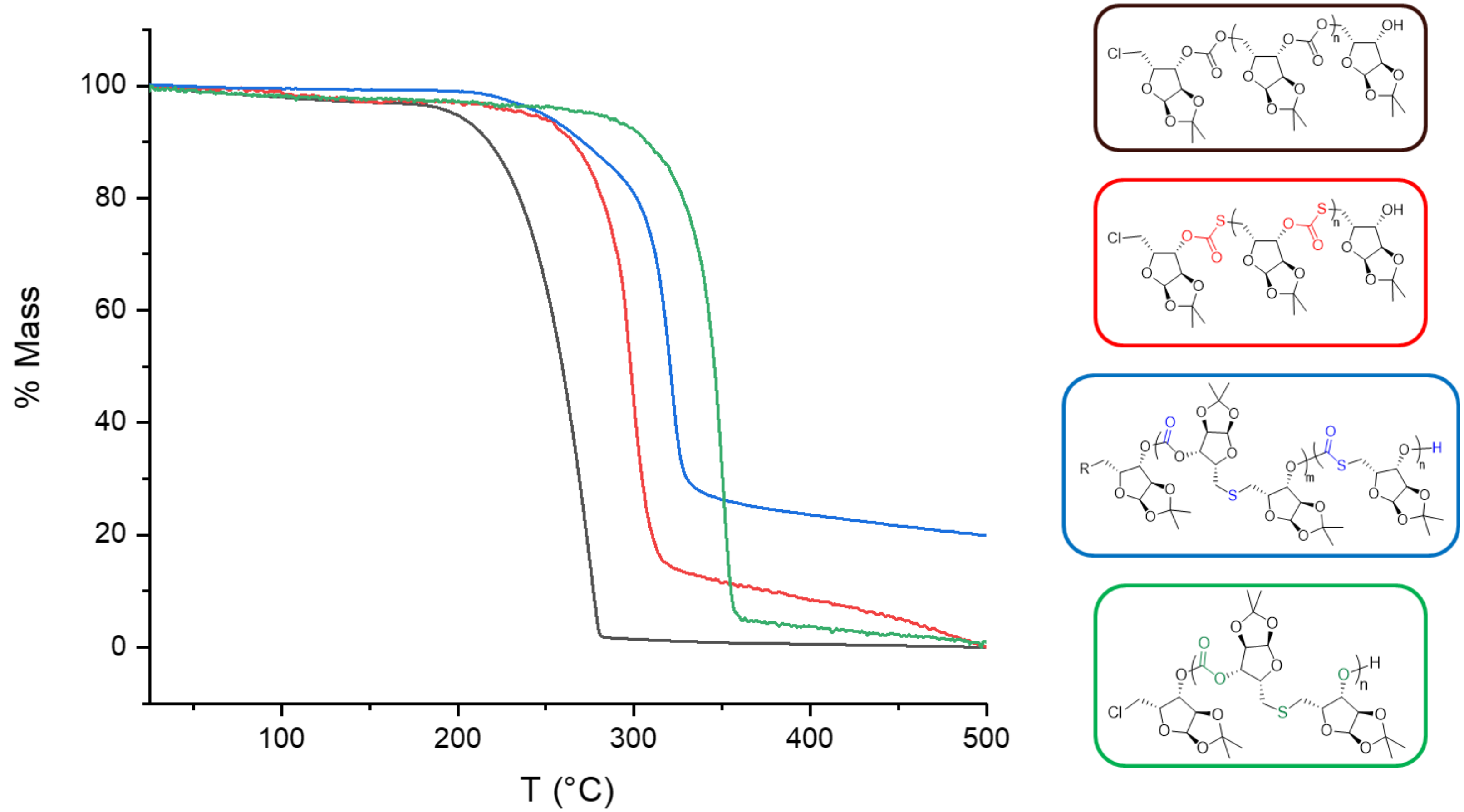

Figure S30: Overlay TGA curves of poly(1,2-*O*-isopropylidene-D-xylofuranose carbonate)[2] (black), poly(MTC) ***7*** (red), poly[(MTC)-*co*-(CTE)] ***9*** (blue), and poly(CTE) ***13*** (green)

---

[2] Tran, D. K.; Rashad, A. Z.; Darensbourg, D. J.; Wooley, K. L. "Sustainable Synthesis of CO_2-derived Polycarbonates from D-Xylose", *Polym. Chem.*, **2021**, *12,* 5271-5278, DOI: 10.1039/D1PY00784J.

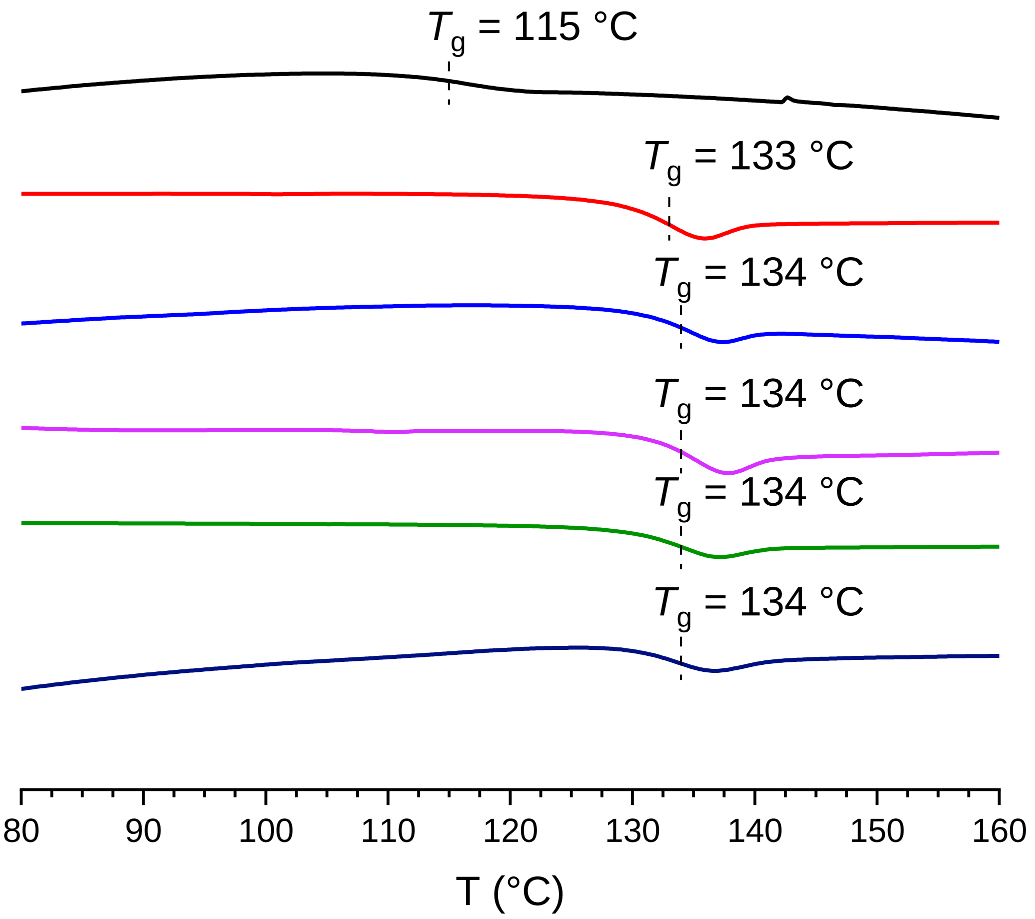

Figure S31: DSC traces of ***1*** (black trace), ***2*** (red trace), ***3*** (blue trace), ***4*** (purple trace), ***5*** (green trace), and ***6*** (dark blue trace). The glass transition temperature (T_g) is labeled on the thermograms.

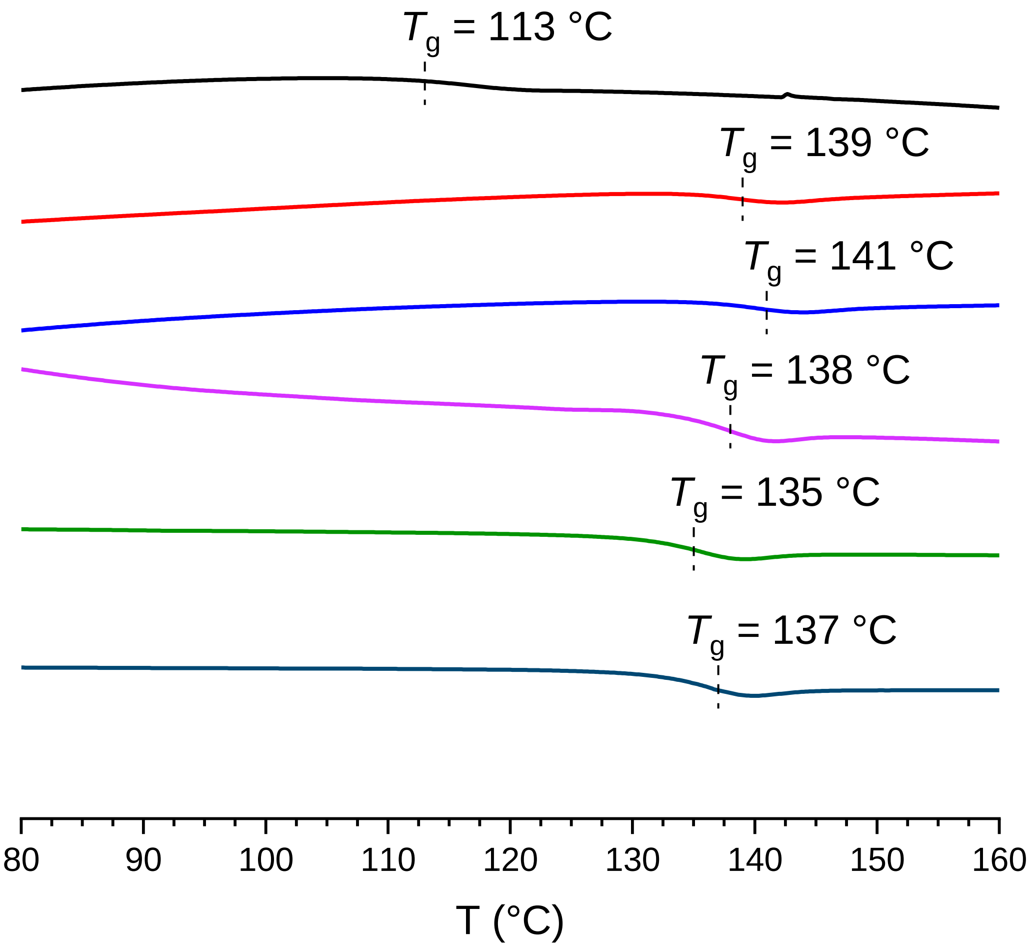

Figure S32: DSC traces of polymers ***7*** (black trace), ***8*** (red trace), ***9*** (blue trace), ***10*** (purple trace), ***11*** (green trace), and ***12*** (dark blue trace). The glass transition temperature (T_g) is labeled on the thermograms.

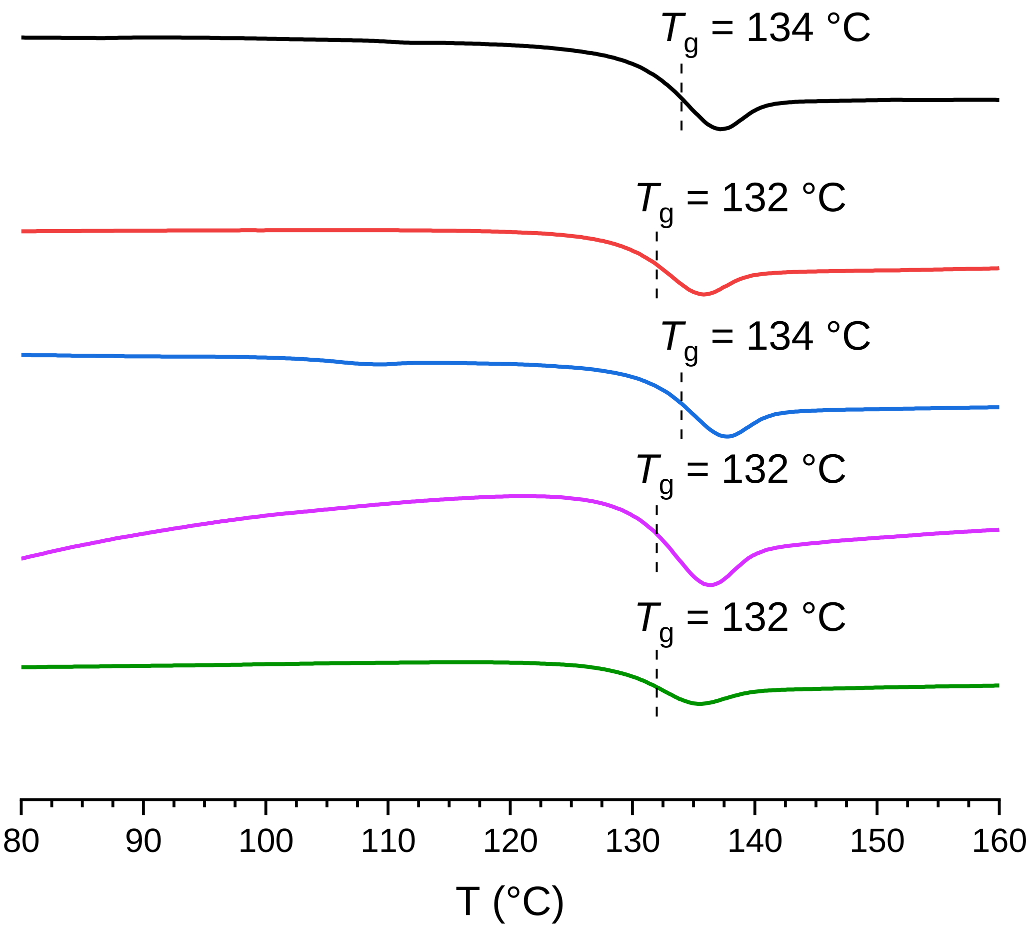

Figure S33: DSC traces of polymers ***13*** (black trace), ***14*** (red trace), ***15*** (blue trace), ***16*** (purple trace), and ***17*** (green trace). The glass transition temperature (T_g) is labeled on the thermograms.

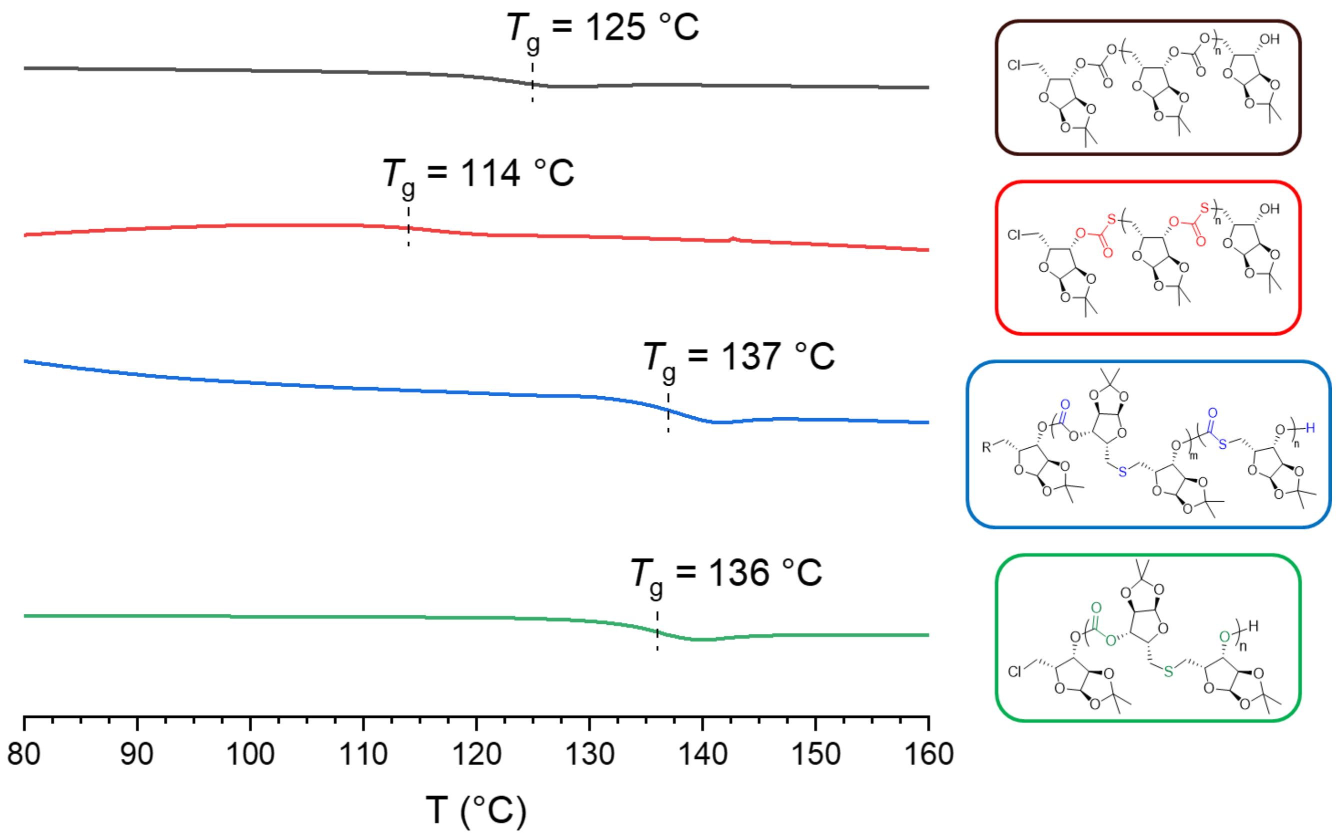

Figure S34: Overlay DSC curves of poly(1,2-*O*-isopropylidene-D-xylofuranose)[3] (black), poly(MTC) ***7*** (red), poly[(MTC)-*co*-(CTE)] ***11*** (blue), and poly(CTE) ***12*** (green).

[3] Tran, D. K.; Rashad, A. Z.; Darensbourg, D. J.; Wooley, K. L. "Sustainable Synthesis of CO_2-derived Polycarbonates from D-Xylose", *Polym. Chem.*, **2021**, *12,* 5271-5278, DOI: 10.1039/D1PY00784J.

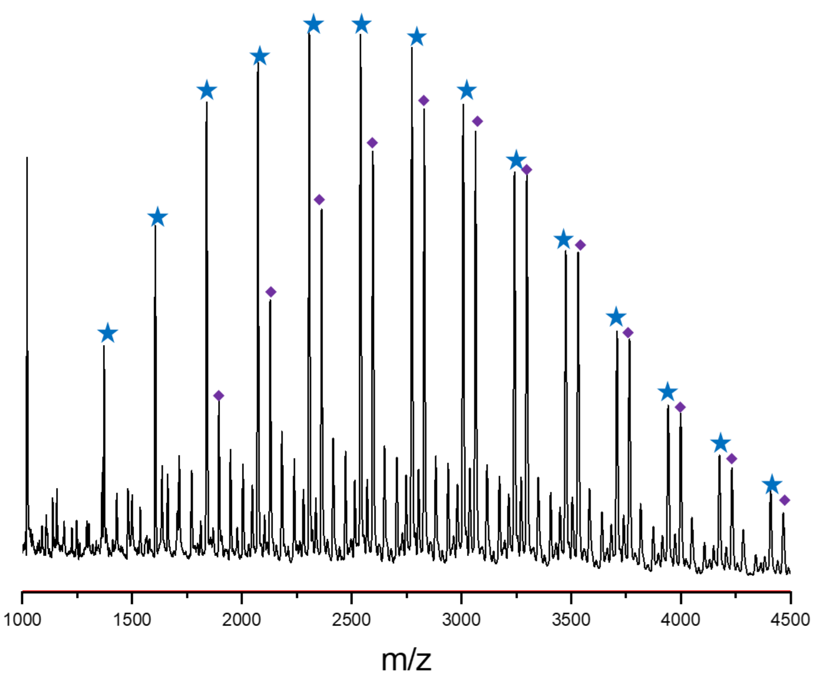

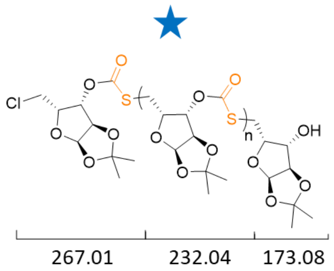

$[M+H^+]_{calc}$	$[M+H^+]_{exp}$
1601.29	1605.02
1833.33	1839.00
2065.37	2072.76
2297.41	2306.42
2529.45	2540.34
2761.49	2773.94
2993.53	3007.70
3225.57	3241.44
3457.61	3473.98
3689.65	3708.50
3921.69	3941.68
4153.73	4175.18

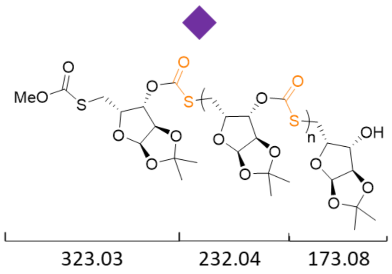

$[M+H^+]_{calc}$	$[M+H^+]_{exp}$
1889.35	1895.04
2121.39	2128.88
2353.43	2362.62
2585.47	2596.36
2817.51	2830.15
3049.55	3064.02
3281.59	3297.62
3513.63	3531.21
3745.67	3764.81
3977.71	3998.35
4209.75	4231.86

Figure S35: MALDI-ToF spectrum of polymer ***7*** produced from ROCOP of COS and xylose oxetane.
